# Short autoinhibitory sequences control phase separation of an essential bacterial transcription termination factor

**DOI:** 10.1038/s44318-026-00793-1

**Published:** 2026-05-11

**Authors:** Emilia Krypotou, Kiersten M Ruff, Leah K McKinney, Guy E Townsend, Jue D Wang, Rohit V Pappu, Eduardo A Groisman

**Affiliations:** 1 Department of Microbial Pathogenesis, Yale School of Medicine, 295 Congress Avenue, New Haven, CT 06536 USA; 2 Department of Biomedical Engineering and Center for Biomolecular Condensates, James F. McKelvey School of Engineering, Washington University, St. Louis, MO 63130 USA; 3 Department of Bacteriology, University of Wisconsin-Madison, Madison, WI 53706 USA; 4 Department of Biochemistry and Molecular Biology, Penn State College of Medicine, 700 HMC Crescent Road, Hershey, PA 17033 USA

**Keywords:** Chromatin, Transcription & Genomics, Microbiology, Virology & Host Pathogen Interaction

## Abstract

Within cells, across diverse organisms, macromolecular condensation enables spatial and temporal organization of biochemical reactions by organizing proteins and nucleic acids into compositionally distinct membraneless biomolecular condensates. In the gut bacterium *Bacteroides thetaiotaomicron*, condensate formation by the transcription termination factor Rho (*Bt*Rho) increases its termination activity and promotes *B. thetaiotaomicron* fitness in the mammalian gut. Here, we elucidate the molecular mechanism governing carbon starvation-induced *Bt*Rho phase separation. We establish that short, specific amino acid sequences within *Bt*Rho’s intrinsically disordered region (IDR) control *Bt*Rho condensation via complex coacervation. The identified sequences participate in RNA and intra-IDR regulatory interactions that drive condensate formation in vitro and in vivo. We also report that the signaling molecule ppGpp is essential for *Bt*Rho phase separation in vivo, binds to purified *Bt*Rho in an IDR-dependent manner, and promotes RNA-dependent *Bt*Rho condensation in vitro. Our findings demonstrate how specific short sequences within an IDR dictate phase separation in response to nutritional cues.

## Synopsis

Phase separation of the bacterial transcription termination factor *Bt*Rho increases its termination activity. This study shows that the alarmone ppGpp and conserved short sequences within *Bt*Rho’s intrinsically disordered region control *Bt*Rho phase separation.

- 9–30 amino acid long conserved sequences in *Bt*Rho IDR mediate interactions with RNA and intra-IDR to drive *Bt*Rho condensation.
- Both sequence and charge of the conserved sequences are critical for interactions with RNA and intra-IDR.
- ppGpp binds to *Bt*Rho in an IDR-dependent manner and is essential for *Bt*Rho condensation in vivo.

## Abstract (web summary)

Conserved short motifs within the intrinsically disordered region of BtRho and binding of ppGpp regulate RNA-driven phase separation in response to nutrient stress.

## Graphical abstract

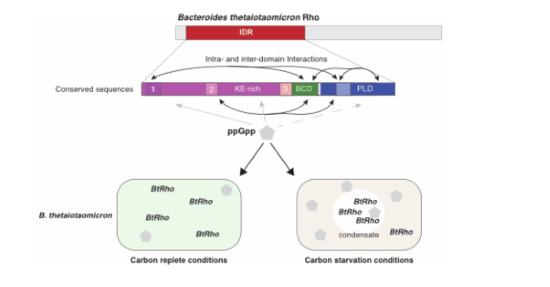

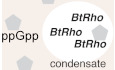

## Introduction

Macromolecular condensation combines reversible binding, percolation, and phase separation that are jointly driven by solubility considerations and networks of synergistic homotypic and heterotypic interactions among proteins, nucleic acids, and small molecules (Farag et al, 2023; Guillen-Boixet et al, 2020; King et al, 2024b; Lin et al, 2023; Mittag and Pappu, 2022; Pappu et al, 2023; Riback et al, 2020; Sanders et al, 2020; Yang et al, 2020b). Condensation organizes biochemical reactions in space and time within cells (Banani et al, 2017) by enabling the formation of compositionally distinct biomolecular condensates with distinct physicochemical properties (Dai et al, 2024b; King et al, 2024b; King et al, 2024c; Lin et al, 2023; Posey et al, 2024; Riback et al, 2020; Sanders et al, 2020; Watson et al, 2023). Responsive condensation enables organisms to react to stresses and other stimuli by generating biomolecular condensates within which distinct environments are created, such that a combination of compositional specificity and distinct solvent properties potentiate spatial and temporal control over biochemical reactions (Dai et al, 2024a; Dai et al, 2024b). Condensates have been proposed to regulate key cellular processes, including gene transcription (Pei et al, 2025), mRNA translation (Franzmann et al, 2018; Kang et al, 2022), RNA storage and metabolism (Al-Husini et al, 2018; Protter and Parker, 2016), and enzyme activity (Jang et al, 2016; Jin et al, 2017; Miura et al, 2013). Here, we show that specific amino acid sequences, RNA, and nutritional signals govern condensation of the essential bacterial transcription termination factor *Bt*Rho, which is required for fitness of the commensal bacterium *Bacteroides thetaiotaomicron* in the murine gut (Krypotou et al, 2023).

Multivalence of specific interaction motifs within folded domains or intrinsically disordered regions (IDRs) is a hallmark of proteins that drive condensation (Banani et al, 2017; Choi et al, 2020; Li et al, 2012; Mittag and Pappu, 2022; Pappu et al, 2023; Wang et al, 2018). IDRs can drive or modulate condensation when harboring sequences with specific molecular grammars, such as non-random compositional biases, sequence patterns, and specific short linear motifs (Cohan et al, 2022; Greig et al, 2020; Martin et al, 2020; Ruff et al, 2022; Sabari et al, 2025; Wang et al, 2018). IDRs featuring blocks of oppositely charged residues can drive condensation via homotypic interactions (King et al, 2024b; Lyons et al, 2023; Zhang et al, 2025). Conversely, polyanionic or polycationic IDRs can drive condensation via complex coacervation, whereby complexation with oppositely charged molecules drives phase separation through heterotypic interactions (Farag et al, 2023; Galvanetto et al, 2023; King et al, 2024b; Pak et al, 2016; Veis, 2011). Arginine- or lysine-rich IDRs as well as RNA recognition motifs in RNA-binding proteins can contribute multivalency for driving complex coacervation with RNA (Seim et al, 2022; Zeke et al, 2022). Many of the relevant IDRs that contribute to condensation with RNA often involve blocks of arginine/lysine residues interspersed with glutamate-rich regions or glycine-rich patches (Chong et al, 2018; Jarvelin et al, 2016). Condensation via complex coacervation involves polyelectrolyte complexation, which is accompanied by the release of atomic or macromolecular counterions and the networking of oppositely charged macromolecules within dense phases (Galvanetto et al, 2023; Neitzel et al, 2021; Priftis and Tirrell, 2012; Rumyantsev et al, 2018; Shinn et al, 2026; Yu et al, 2021). Networks of short-range interactions among oppositely charged macromolecules that involve aromatic residues and/or hydrophobic motifs can enhance the driving forces for complex coacervation (Pak et al, 2016).

**Figure 1:**
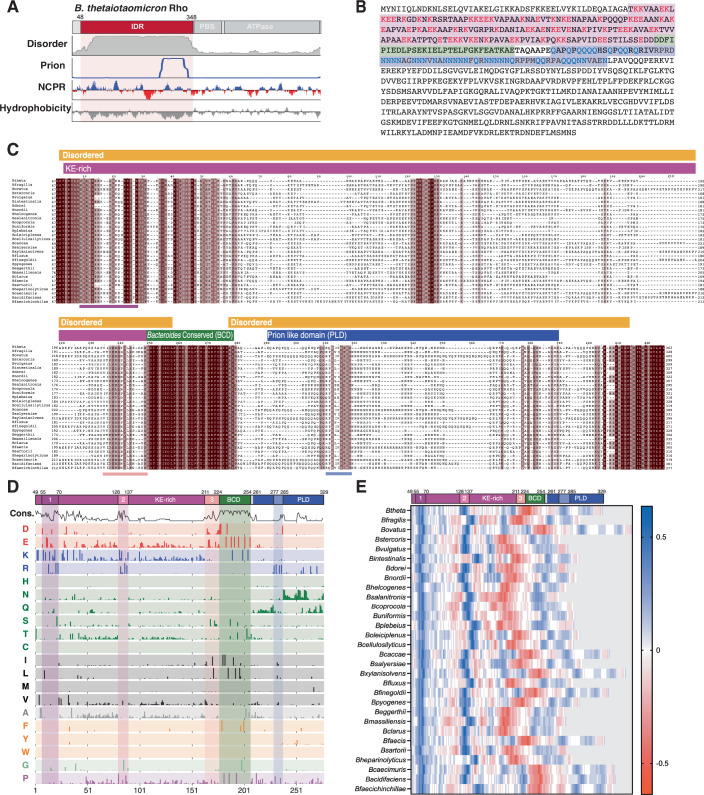
The IDRs of *Bacteroides* Rho sequelogs harbor amino acid stretches conserved in sequence and/or physicochemical properties. (**A**) In silico analysis of the deduced amino acid sequence of *B. thetaiotaomicron* Rho (*Bt*Rho). Disorder was predicted using AIUPRED (Erdos and Dosztanyi, 2024), prion-like domains were predicted using PLAAC (Lancaster et al, 2014), the net charge per residue (NCPR) was predicted using EMBOSS (Rice et al, 2000) with a sliding window of 11 amino acids, and hydrophobicity was calculated based on Kyte & Doolittle and a sliding window of 11 amino acids. PBS, primary RNA-binding site; ATPase, ATP-hydrolyzing domain of Rho. The residue numbers of the IDR are also indicated. Note that disorder prediction can differ slightly based on the software used. (**B**) The deduced amino acid sequence of *Bt*Rho with the KE-rich subdomain highlighted with pink, the *Bacteroides* Conserved domain (BCD) with green, and the prion-like domain (PLD) with blue. Charged residues (Lys and Glu) in the KE-rich subdomain are in red; Asn and Gln are in blue. (**C**) Multiple sequence alignment of Rho IDRs from the indicated *Bacteroides* species. The dark shaded boxes correspond to different degrees of sequence conservation. The predicted protein disorder is indicated with an orange box, the KE-rich subdomain with pink, the BCD with green and the PLD with blue (note that the PLD subdomain ends at a different residue than the predicted disordered region). Colored boxes below the alignment indicate short, conserved motifs. (**D**) For the multiple sequence alignment shown in (**C**), the conservation score (0–11) for each non-gap position based on the deduced amino acid sequence of *Bt*Rho was extracted from Jalview and shown as a black curve. In addition, the fraction of each amino acid for each position was calculated and depicted as histograms. A value of 1 indicates that the same amino acid was present at the given aligned position across the 30 species. The regions corresponding to the KE-rich, BCD, and PLD and the identified conserved motifs are indicated using the color scheme used in (**C**). (**E**) Heatmap of amino acid charge per position (EMBOSS (Rice et al, 2000), sliding window 11 amino acids) for the IDR sequences shown in the alignment. The regions corresponding to the KE-rich, BCD, and PLD, and the identified conserved motifs are also indicated. The residue numbers of the subdomains and motifs are also indicated. The regions corresponding to the KE-rich, BCD, and PLD and the identified conserved motifs are indicated using the color scheme used in (**C**). Source data are available online for this figure.

Rho is an RNA helicase that mediates transcription termination at the end of genes or operons (Do et al, 2025; Mitra et al, 2017). Rho also mediates transcription termination within mRNA leaders where it implements regulatory decisions, and whenever transcription and translation become uncoupled in gram-negative species (Hao et al, 2021; Kriner et al, 2016). Rho is present in ~90% of bacteria and essential in gram-negative species (Moreira et al, 2024). Roughly 40% of Rho proteins harbor an extra domain near the N-terminus that is absent from the canonical Rho proteins of *Escherichia coli*, *Salmonella enterica* serovar Typhimurium, and *Bacillus subtilis*, and this region is predicted to be intrinsically disordered (Moreira et al, 2024). The 722 residue-long *Bt*Rho harbors a 301-amino acid long IDR (Fig. 1A) that is necessary for condensation in vivo and in vitro and for increased transcription termination activity under conditions that favor condensation (Krypotou et al, 2023).

Here, we report the identification of short, charge-rich, conserved motifs within the IDR from *Bt*Rho that govern condensation in response to carbon starvation, a condition experienced by *B. thetaiotaomicron* in the mammalian gut (Groisman et al, 2023) that elicits *Bt*Rho phase separation (Krypotou et al, 2023) and synthesis of proteins that promote *B. thetaiotaomicron* fitness in the mammalian gut (Han et al, 2023; Townsend et al, 2020). We identify interactions within the IDR and with RNA that are mediated by the charge-rich motifs. We also show that *Bt*Rho condensation induced by carbon starvation requires ppGpp, a signaling molecule synthesized upon carbon starvation and required for *B. thetaiotaomicron* fitness in the gut (Schofield et al, 2018), and that ppGpp binds to purified *Bt*Rho in an IDR-dependent manner and promotes *Bt*Rho condensation in the presence of RNA in vitro. Our findings uncover the mechanism by which a nutritional stress governs phase separation of an essential transcription factor.

## Results

### The *Bt*Rho IDR is composed of three distinct subdomains

To understand how the IDR from *Bt*Rho promotes condensation of *Bt*Rho, we analyzed its amino acid sequence and composition to uncover molecular grammars that may contribute to the driving forces for condensation. We identified two distinct disordered regions that differ in amino acid composition: (i) a lysine- and glutamate-rich 176 residue-long region (Fig. 1B–D), designated the KE-rich subdomain, and (ii) an asparagine- and glutamine-rich 68-residue-long region predicted to be a prion-like domain (Fig. 1A–D), designated the PLD subdomain. Sequence alignment of the IDRs from Rho homologs in 30 *Bacteroides* species revealed conservation in amino acid content among the KE-rich and PLD subdomains (Fig. 1C). However, neither the length nor the overall sequence was conserved (Fig. 1C). The only exceptions showing high sequence conservation were a few short stretches that are akin to short linear motifs (SLiMs) that are often found within IDRs (highlighted with colored boxes in Fig. 1C,D) (Davey et al, 2012). The KE-rich and PLD subdomains flank a 30 amino acid-long sequence that is highly conserved among *Bacteroides* species, in contrast to either subdomain (Fig. 1B–D). We refer to this region as the *Bacteroides* conserved domain (BCD).

Next, we examined the amino acid charge distribution in the *Bacteroides* IDRs using EMBOSS (Rice et al, 2000) to calculate the net charge per position. Although ionizable residues are present across the length of the IDRs, we identified five short stretches with higher charge density than the rest of the sequence (Fig. 1E). These charge-dense stretches, three of which are positively charged and two of which are negatively charged, are at similar locations in all examined IDRs from *Bacteroides* Rho proteins and conserved at the sequence level (colored boxes in Fig. 1C–E). Overall, our analysis of the *Bacteroides* Rho IDRs revealed similar amino acid compositions and the presence of five conserved 9–30 amino acid-long sequence stretches of high charge density.

### The KE-rich subdomain drives RNA-dependent condensation, whereas the BCD and PLD subdomains modulate this condensation

**Figure 2:**
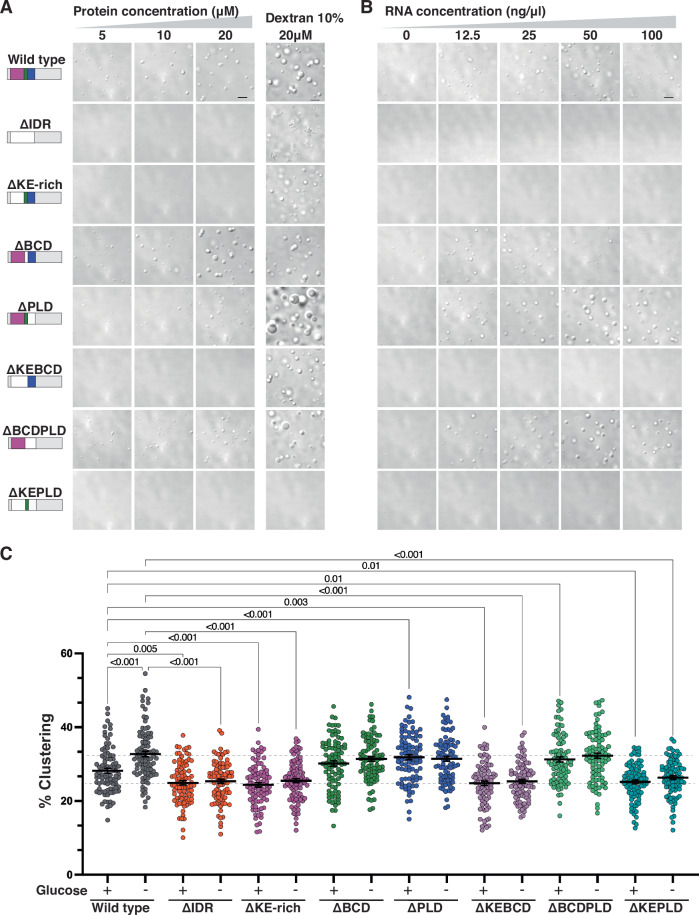
*Bt*Rho condensation is driven by interactions between the KE-rich subdomain and RNA and controlled by the *Bacteroides* conserved domain and prion-like domain subdomains. (**A**) Differential interference contrast (DIC) microscopy of in vitro condensation assays of wild-type *Bt*Rho and variants at the indicated protein concentration with or without 10% w/v dextran. The results corresponding to the whole range of concentrations tested (i.e., 0.625 μM to 20 μM) are shown in Figs. EV1 and EV2. Three independent experiments were performed, and a representative experiment is shown. Scale bar: 5 μm. (**B**) DIC microscopy of condensation assays of wild-type *Bt*Rho protein and variants (2.5 μM) in the presence of increasing RNA amounts corresponding to total RNA extract from *B. thetaiotaomicron* (see also Fig. EV3). Three independent experiments were performed, and a representative experiment is shown. Scale bar: 5 μm. (**C**) In vivo *Bt*Rho condensation calculated as % Clustering in *B. thetaiotaomicron* strains expressing HA-tagged versions of the indicated proteins (wild type: AK82, ΔIDR: AK86, ΔKE-rich: AK408, ΔBCD: AK614, ΔPLD: AK427, ΔKEBCD: AK527, ΔBCDPLD: AK525, and ΔKEPLD: AK616). Bacteria were grown in glucose (+) until mid-exponential phase and then shifted to media without any carbon source for 30 min (−). Data points represent clustering values of individual cells from three independent experiments (*n* = 90), black bars are mean values and error bars represent SEM. Dashed lines indicate levels of clustering for wild-type *Bt*Rho in carbon starvation and for the ΔIDR *Bt*Rho under both conditions. One-way ANOVA was performed, followed by pairwise comparisons between the wild-type protein and the variants for the same growth condition or between the two conditions for the same strain. *P* values < 0.05 are shown. Šidak’s test was used to correct for multiple comparisons. Source data are available online for this figure.

**Figure EV1:**
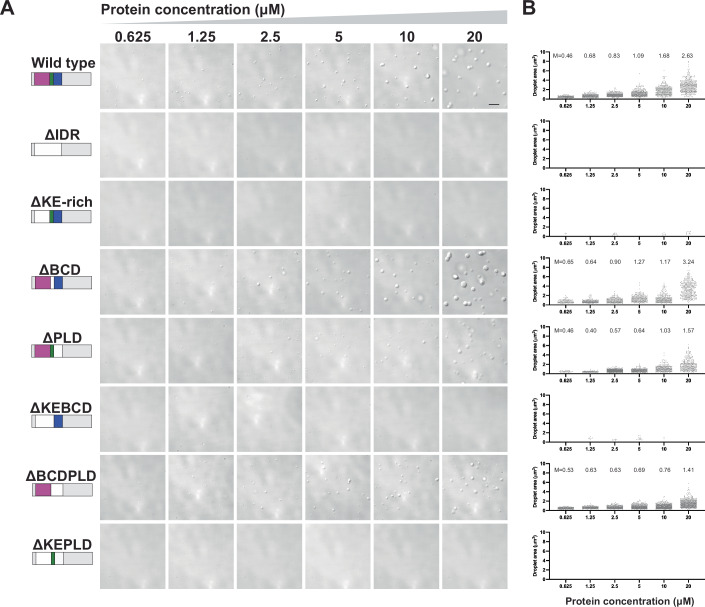
*Bt*Rho variants harboring the KE-rich or PLD subdomains drive condensation. (**A**) DIC microscopy of in vitro condensation assays of wild-type *Bt*Rho and variants at the indicated protein concentrations. The results corresponding to protein concentrations of 5, 10, and 20 μΜ are also shown in Fig. 2A. Three independent experiments were performed, and a representative experiment is shown. Scale bar: 5 μm. (**B**) Quantification of droplet size formed in (**A**) for each *Bt*Rho variant. For each condition, the counted droplets were from three different fields of view of the same sample. The median (M) value is also indicated for each condition.

**Figure EV2:**
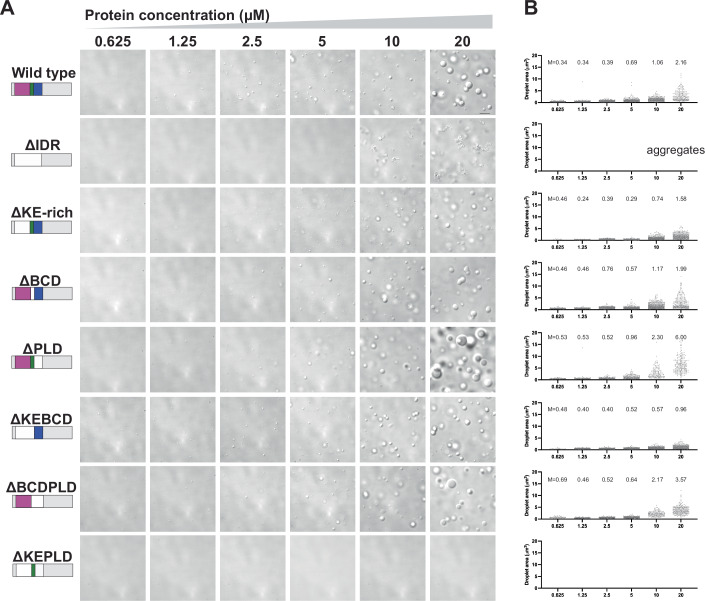
*Bt*Rho variants harboring the KE-rich or PLD subdomains form large condensates in the presence of the crowding factor dextran. (**A**) DIC microscopy of in vitro condensation assays of wild-type *Bt*Rho and variants at the indicated protein concentration and in the presence of 10% w/v dextran. The results corresponding to protein concentration 20 μΜ are also shown in Fig. 2A. Three independent experiments were performed, and a representative experiment is shown. Scale bar: 5μm. (**B**) Size quantification of droplets formed in (**A**) for each protein variant. For each condition the droplets counted were from 3 different fields of view of the same sample. The median (M) value is also indicated for each condition.

To determine the specific roles of the different subdomains from the *Bt*Rho IDR (Fig. 1B), we investigated phase separation of *Bt*Rho variants lacking one or more subdomains as purified proteins. Wild-type *Bt*Rho formed spherical condensates that increased in size and abundance as the protein concentration increased (Figs. 2A and EV1). By contrast, a *Bt*Rho derivative lacking the IDR did not form condensates (Figs. 2A and EV1). These results are in accord with previous findings (Krypotou et al, 2023). In the presence of the crowding agent dextran (10% w/v), the size of the condensates formed by the wild-type *Bt*Rho increased, while the ΔIDR derivative formed irregularly shaped, dynamically arrested assemblies at high protein concentrations (Figs. 2A and EV2).

A variant lacking the KE-rich subdomain formed condensates only in the presence of dextran, and the condensates were smaller than those formed by the wild-type protein (Figs. 2A, EV1, and EV2). A variant lacking the PLD subdomain retained the ability to form spherical condensates that were smaller in size compared to the wild type in the absence of dextran, but larger in size compared to the wild type in the presence of dextran (Figs. 2A, EV1, and EV2). By contrast, a variant lacking both the KE-rich and PLD subdomains did not form condensates under the investigated conditions (Figs. 2A, EV1, and EV2). These results establish that *Bt*Rho condensation requires either the KE-rich or PLD subdomain (together with the BCD).

At equivalent protein concentrations and identical solution conditions, the sizes and abundance of condensates are useful proxies for comparative assessments of driving forces for condensation, with higher abundance reflecting stronger driving forces. Condensates can grow in size via different coarsening mechanisms, and this typically involves the depletion of smaller condensates in favor of larger ones (Berry et al, 2018). The ability to coarsen is a measure of the relative contributions of interfacial tension versus the driving forces for fusion. Together, high abundance and larger sizes imply strong driving forces for condensation.

The *Bt*Rho variant lacking the BCD formed condensates larger than the wild-type protein in the absence of dextran but similar to the wild-type protein in the presence of dextran (Figs. 2A, EV1, and EV2). The variant lacking both the BCD and PLD subdomains behaved similarly to the variant lacking only the PLD subdomain without dextran and showed a small decrease in the size of the droplets in the presence of dextran (Figs. 2A, EV1, and EV2). The *Bt*Rho variant that retained only the PLD subdomain of the IDR did not form condensates in the absence of dextran. However, in the presence of dextran, the variant that retained only the PLD subdomain formed condensates that were smaller than those formed by wild-type *Bt*Rho and similar to those formed by the variant retaining both the BCD and PLD subdomains (Figs. 2A, EV1, and EV2).

Inert crowders enhance the driving forces for condensation via depletion-mediated attractions (Chauhan et al, 2024). However, crowders can also engage in heterotypic interactions and modulate the driving forces for condensation (Andre et al, 2023; Qian et al, 2024). Our results suggest that removing the PLD subdomain unmasks heterotypic interactions that enhance driving forces for condensation in the presence of dextran and that are otherwise masked in the wild-type protein. Overall, the data presented above indicate that the presence of either the KE-rich or PLD subdomain can drive *Bt*Rho condensation, albeit only in the presence of dextran for the latter. Moreover, the different sensitivity to dextran suggests that each subdomain contributes differently to *Bt*Rho condensation: the KE-rich subdomain engages mainly in homotypic interactions, whereas the PLD contributes via a blend of homotypic and heterotypic interactions. In contrast to the KE-rich and PLD subdomains, the BCD suppresses condensation.

**Figure EV3:**
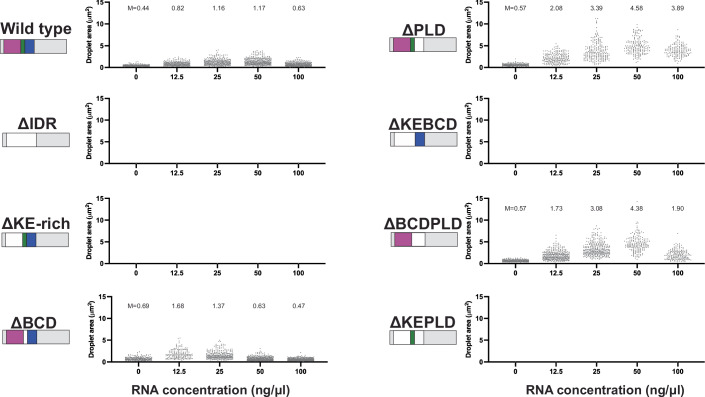
The KE-rich subdomain is required for RNA-dependent *Bt*Rho condensation in vitro. Size quantification of droplets formed in Fig. 2B for each investigated protein. For each condition the droplets counted were from three different fields of view of the same sample. The median (M) value is also indicated for each condition.

Heterotypic interactions with RNA modulate condensation of wild-type *Bt*Rho (Krypotou et al, 2023). Particularly, reentrant phase separation is observed such that the RNA:protein concentration ratio enables entry into and exit from the two-phase regime (Krypotou et al, 2023) (Fig. 2B). We next examined the behavior of *Bt*Rho variants in the presence of increasing amounts of total RNA extracted from *B. thetaiotaomicron* using protein (2.5 μM) and salt (150 mM) concentrations at near physiological levels (the intracellular *Bt*Rho level was previously determined to be ~1 μM (Krypotou et al, 2023)). The *Bt*Rho variants lacking the KE-rich subdomain did not form condensates in the presence of RNA (Figs. 2B and EV3). A variant lacking the BCD subdomain showed increased condensation at low RNA concentrations but less condensation at high RNA concentrations compared to the wild-type protein (Figs. 2B and EV3). By contrast, the variants lacking the PLD subdomain, or both the BCD and PLD, displayed greater extents of condensation than wild-type *Bt*Rho but diminished reentrant behaviors (Figs. 2B and EV3). These results indicate that the KE-rich subdomain is essential for RNA-dependent condensation and that the BCD and PLD modulate interactions between the KE-rich subdomain and RNA.

### The KE-rich subdomain is essential for *Bt*Rho condensation in vivo, and the PLD subdomain is required for control of *Bt*Rho condensation by nutritional signals

To determine the role of the *Bt*Rho subdomains on condensation in vivo, we used immunostaining and fluorescence microscopy to visualize isogenic *B. thetaiotaomicron* strains deleted for the wild-type *rho* gene and expressing the *Bt*Rho variants discussed above. Note that even though *Bt*Rho is essential, a mutant lacking the IDR of *Bt*Rho grows like wild-type *B. thetaiotaomicron* in laboratory media (Krypotou et al, 2023). We examined bacteria grown to mid-exponential phase in defined minimal media with glucose as the carbon source and 30 min after bacteria were transferred to media lacking a carbon source, a condition that promotes condensation of wild-type *Bt*Rho (Krypotou et al, 2023). As described in Materials and Methods and previously (Krypotou et al, 2023), we quantified the protein cellular dispersion as a % clustering index by measuring the fluorescence intensity per pixel and determining the % normalized intensity below a set threshold. Uniform signal distribution corresponds to less clustering, while presence of foci with high fluorescence intensity corresponds to higher clustering. Control experiments demonstrated significantly higher condensation for wild-type *Bt*Rho than for ΔIDR *Bt*Rho in both conditions (Fig. 2C), as previously reported (Krypotou et al, 2023).

Strains expressing variants lacking the KE-rich subdomain alone or in combination with any other subdomains displayed lower extents of condensation, thus phenocopying the effects of the ΔIDR *Bt*Rho variant (Fig. 2C). By contrast, strains expressing variants lacking the BCD or PLD exhibited similarly high levels of condensation during carbon starvation, equivalent to that of the strain expressing wild-type *Bt*Rho during carbon starvation (Fig. 2C). However, the PLD-lacking variants had high levels of condensation also during growth on glucose (Fig. 2C), whereas condensation of the variant lacking only the BCD was not significantly higher than condensation of wild-type *Bt*Rho in this condition (Fig. 2C).

Taken together, the results presented above suggest that RNA is a key driver of *Bt*Rho phase separation in vivo. This is because the KE-rich subdomain is essential for RNA-dependent condensation in vitro (Figs. 2B and EV3). In addition, these results indicate that the PLD operates as an inhibitory element that suppresses *Bt*Rho condensation when glucose is present.

### Short, conserved, charged motifs within the KE-rich subdomain contribute to RNA-mediated *Bt*Rho condensation

**Figure 3:**
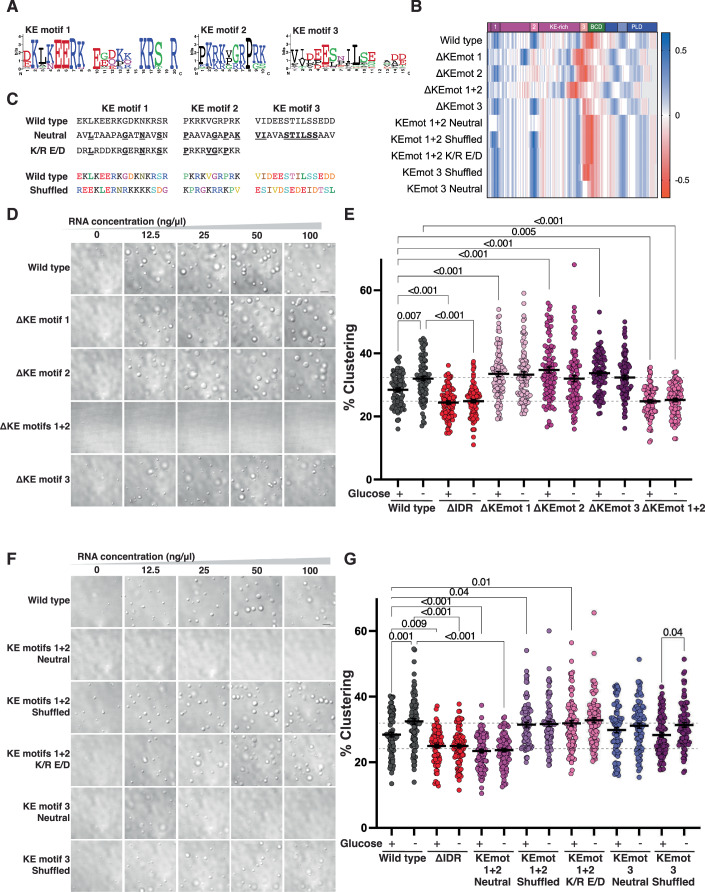
Short, conserved motifs within the KE-rich subdomain are required for RNA-dependent condensation of *Bt*Rho. (**A**) Sequence logo of the conserved motifs within the KE-rich subdomain highlighted in Fig. 1C–E. (**B**) Heatmap of the amino acid charge per residue (EMBOSS (Rice et al, 2000), sliding window 11 amino acids) for wild-type *Bt*Rho and variants of the *Bt*Rho IDR. The regions corresponding to the KE-rich, BCD, and PLD, and *Bt*Rho conserved motifs are also indicated. (**C**) Amino acid sequences of the wild-type KE motifs 1, 2 and 3 and mutants harboring amino acid substitutions in these motifs. (**D**) DIC microscopy of in vitro condensation assays of wild-type *Bt*Rho and variants (2.5 μM) in the presence of increasing RNA amounts corresponding to total RNA extract from *B. thetaiotaomicron* (see also Fig. EV4A). Three independent experiments were performed, and a representative experiment is shown. Scale bar: 5 μm. (**E**) In vivo *Bt*Rho condensation calculated as % Clustering in *B. thetaiotaomicron* strains expressing HA-tagged versions of the indicated proteins (wild type: AK82, ΔIDR: AK86, ΔΚΕ motif 1: AK519, ΔΚΕ motif 2: AK545, ΔΚΕ motif 3: AK582, ΔΚΕ motifs 1 + 2: AK542). Bacteria were grown in glucose (+) until mid-exponential phase and then shifted to media without any carbon source for 30 min (−). Data points represent clustering values of individual cells from three independent experiments (*n* = 90), black bars are mean values and error bars represent SEM. Dashed lines are used as visual aids to indicate the degrees of clustering for wild-type *Bt*Rho in carbon starvation and for the ΔIDR *Bt*Rho. One-way ANOVA was performed, followed by pairwise comparisons between the wild-type *Bt*Rho and the variants for the same growth condition or between the two conditions for the same strain. *P* values < 0.05 are shown. Šidak’s test was used to correct for multiple comparisons. (**F**) DIC microscopy of in vitro condensation assays of wild-type *Bt*Rho and variants (2.5 μM) in the presence of increasing RNA amounts corresponding to total RNA extract from *B. thetaiotaomicron* (see also Fig. EV4B). Three independent experiments were performed, and a representative experiment is shown. Scale bar: 5 μm. (**G**) In vivo *Bt*Rho condensation calculated as % Clustering in *B. thetaiotaomicron* strains expressing HA-tagged versions of the indicated proteins (wild type: AK600, ΔIDR: AK602, KEmotifs 1 + 2 Neutral: AK610, KEmotifs 1 + 2 Shuffled: AK633, KEmotifs 1 + 2 K/R E/D: AK612, KEmotif 3 Neutral: AK654, and KEmotif 3 Shuffled: AK656). Bacteria were grown in glucose (+) until mid-exponential phase and then shifted to media without any carbon source for 30 min (−). Data points represent clustering values of individual cells from three independent experiments (*n* = 90), black bars are mean values and error bars represent SEM. Dashed lines are used as visual aids to indicate the degrees of clustering for wild-type *Bt*Rho in carbon starvation and for the ΔIDR *Bt*Rho. One-way ANOVA was performed, followed by pairwise comparisons between the wild-type *Bt*Rho and the variants for the same growth condition or between the two conditions for the same strain. *P* values < 0.05 are shown. Šidak’s test was used to correct for multiple comparisons. Source data are available online for this figure.

We identified three highly charged motifs in the 176 residue-long KE-rich subdomain that are conserved among *Bacteroides* Rho proteins. KE motif 1 and KE motif 2 (16 and 10 residues long, respectively) share a dense positive charge due to conserved lysine and arginine residues, and KE motif 3 (14 residues long) is negatively charged due to conserved glutamate and aspartate residues (see Figs. 1 and 3A–C). To determine the roles of these three motifs in *Bt*Rho condensation in vitro, we first examined variants lacking these motifs (Fig. 3B).

**Figure EV4:**
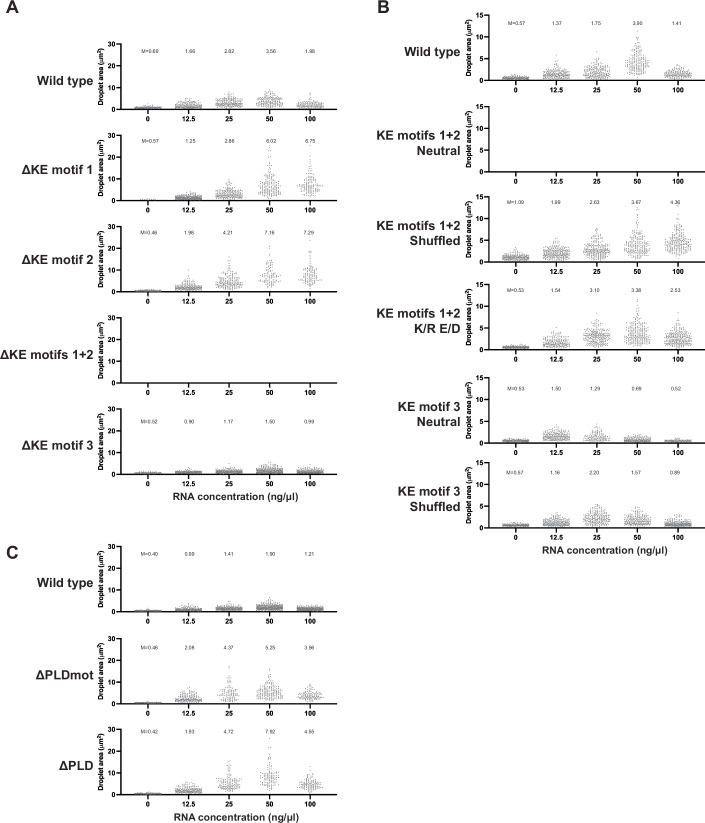
Sequence motifs within the KE-rich and PLD subdomains control RNA-dependent *Bt*Rho condensation in vitro. Size quantification of droplets formed in Fig. 3D (**A**), Fig. 3F (**B**) and Fig. 4C (**C**) for the indicated variants. For each condition, the droplets counted were from three different fields of view of the same sample. The median (M) value is also indicated for each condition.

Variants lacking KE motif 1 or KE motif 2 formed condensates that were larger in size when compared to those formed by wild-type *Bt*Rho (Figs. 3D and EV4A). The condensates formed by these two variants also differed from wild-type *Bt*Rho in their reentrant behavior: the number and size of condensates remained the same when the RNA concentration increased from 50 to 100 ng/µl, which is in contrast to the lower number of condensates exhibited by wild-type *Bt*Rho over the same RNA concentration range (Figs. 3D and EV4A).

The variant lacking ΚΕ motif 3 formed a high number of condensates that were smaller in size than those formed by wild-type *Bt*Rho and the condensates retained the reentrant behavior as the size of condensates depended on the RNA and protein concentrations (Figs. 3D and EV4A), thus contrasting the behaviors of the variants lacking KE motif 1 or KE motif 2.

Next, we investigated a variant lacking both KE motif 1 and KE motif 2. This variant behaved like mutants lacking the entire KE-rich subdomain or the IDR, showing an inability to form condensates in the presence of RNA (Figs. 3D and EV4A). Taken together with the results discussed above, these data suggest that the positively charged KE motif 1 and KE motif 2 drive co-condensation with RNA via complex coacervation (King et al, 2024a; Pak et al, 2016). Thus, when both of these motifs are absent, *Bt*Rho-RNA interactions are highly restricted, hindering condensation. By contrast, the negatively charged KE motif 3 regulates condensation.

*B. thetaiotaomicron* strains expressing *Bt*Rho variants lacking ΚΕ motif 1, ΚΕ motif 2, or ΚΕ motif 3 showed similarly high degrees of clustering during growth on glucose or carbon starvation, like those exhibited by the strain expressing wild-type *Bt*Rho during carbon starvation (Fig. 3E). In stark contrast, condensation was completely lost in the mutant lacking both KE motif 1 and KE motif 2 (Fig. 3E), which behaved similarly to the ΔIDR *Bt*Rho mutant. Given that the mutant lacking both KE motif 1 and KE motif 2 failed to form condensates in the presence of RNA in vitro (Fig. 3D), we infer that RNA interactions with KE motif 1 and KE motif 2 are the main drivers of *Bt*Rho condensation in vivo.

### The charge and sequence of the KE-rich motifs govern *Bt*Rho condensation

**Figure EV5:**
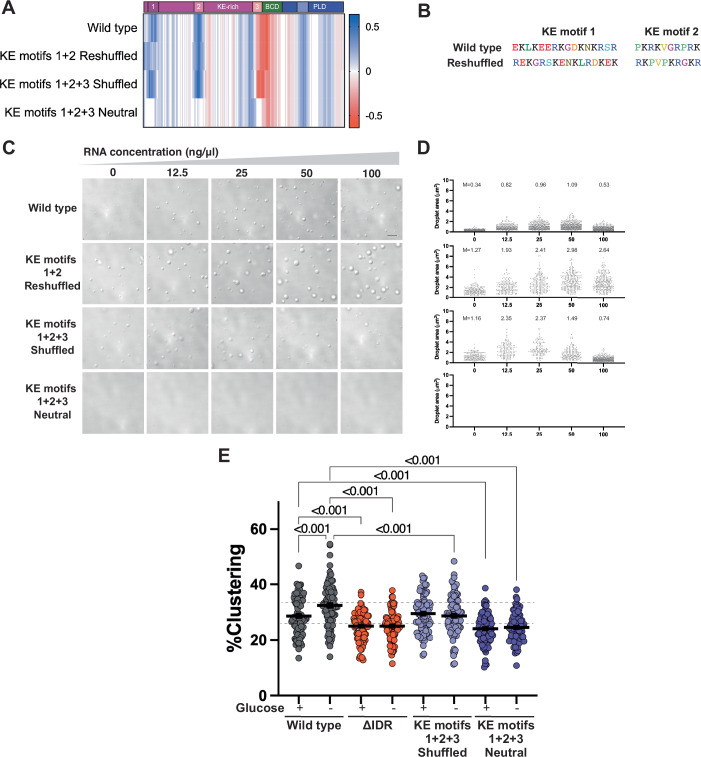
KE motifs 1, 2, and 3 control *Bt*Rho condensation. (**A**) Heatmap of the amino acid charge per residue (EMBOSS (Rice et al, 2000), sliding window 11 amino acids) for wild-type *Bt*Rho and the indicated variants. (**B**) Amino acid sequence of wild-type KE motifs 1 and 2 and of the indicated variants. (**C**) DIC microscopy of in vitro condensation assays of wild-type *Bt*Rho and variants (2.5 μM) in the presence of increasing RNA amounts corresponding to total RNA extract from *B. thetaiotaomicron*. Three independent experiments were performed, and a representative experiment is shown. Scale bar: 5 μm. (**D**) Size quantification of droplets formed in (**C**) for each variant. For each condition, the droplets counted were from three different fields of view of the same sample. The median (M) value is also indicated for each condition. (**E**) In vivo *Bt*Rho condensation calculated as % Clustering in *B. thetaiotaomicron* strains expressing HA-tagged versions of the indicated proteins (wild type: AK600, ΔIDR: AK602, ΚΕmotifs 1 + 2 + 3 Shuffled: AK660, and KEmotifs 1 + 2 + 3 Neutral: AK658). Bacteria were grown in glucose (+) until mid-exponential phase and then shifted to a media without any carbon source for 30 min (−). Data points represent clustering values of individual cells from three independent experiments (*n* = 90), black bars are mean values and error bars represent SEM. Dashed lines are used as visual aids to indicate the levels of clustering for wild-type *Bt*Rho in carbon starvation and for ΔIDR *Bt*Rho. One-way ANOVA was performed, followed by pairwise comparisons between wild-type and the mutant variants for the same growth condition or between the two conditions for the same strain. *P* values < 0.05 are shown. Šidak’s test was used to correct for multiple comparisons.

To determine whether changes in high charge density versus amino acid sequence are responsible for the phenotypes of the *Bt*Rho variants lacking one or more KE-rich motifs, we engineered a set of *Bt*Rho variants with amino acid substitutions that: (i) neutralized the charge (KE motifs 1 + 2 Neutral, KE motif 3 Neutral, and KE motifs 1 + 2 + 3 Neutral); (ii) retained the charge and amino acid composition but not the amino acid sequence (KE motifs 1 + 2 Shuffled, KE motifs 1 + 2 Reshuffled, KE motif 3 Shuffled, KE motifs 1 + 2 + 3 Shuffled); and (iii) retained the total charge and location of the charge but with different amino acids (i.e., replacing lysine with arginine and glutamate with aspartate, and vice versa (K/R E/D) (Figs. 3B,C and EV5A,B).

Neutralizing the charges of KE motif 1 and KE motif 2 led to a complete loss of condensation in the presence of RNA in vitro (Fig. 3F), resembling the behavior of the variant lacking KE motif 1 and KE motif 2 (Fig. 3D) and affirming that complex coacervation is the mode of condensation. By contrast, the variants that retained the positive charge but not the sequence of KE motif 1 and KE motif 2 (Shuffled, Reshuffled, K/R E/D) formed more condensates that were larger in size than those formed by wild-type *Bt*Rho (Figs. 3F, EV4B, and EV5C,D). The reentrant behavior of these mutants also differed from that of wild-type *Bt*Rho because they formed large condensates in the presence of high RNA concentrations (Figs. 3F, EV4B, and EV5C,D). These results, which highlight the impact of charge patterning and linear charge density on complex coacervation (King et al, 2024a; Pak et al, 2016), indicate that the positive charge in KE motifs 1 and 2 is essential for RNA-dependent condensation of wild-type *Bt*Rho and that the specific amino acid sequences of KE motifs 1 and 2 play a critical role in *Bt*Rho co-condensation with RNA.

The Neutral variant of KE motif 3 formed fewer condensates than wild-type *Bt*Rho, and the condensates were dissolved as the RNA concentration increased (Figs. 3F and EV4B). By contrast, the shuffled variant behaved like wild-type *Bt*Rho, showing only a small decrease in the size of the droplets and retaining reentrant behavior (Figs. 3F and EV4B). A variant in which all three charged KE motifs were rendered neutral (KE motifs 1 + 2 + 3 Neutral) did not form condensates (Fig. EV5C), behaving like the variant with KE motifs 1 and 2 neutralized (Fig. 3F). Simultaneous shuffling of the sequences of the three KE motifs resulted in a moderate increase in the size of condensates (Fig. EV5C,D). Thus, the charge of KE motif 3 appears to dictate the abilities of KE motif 1 and KE motif 2 to interact with RNA, thereby regulating condensation of *Bt*Rho.

In vivo, the interactions of RNA with KE motif 1 and KE motif 2 appear to drive *Bt*Rho condensation because neutralizing the charges of these two motifs hindered condensate formation. This behavior resembled that of the ΔIDR *Bt*Rho-expressing strain (Fig. 3G), which is defective in condensation in vitro even in the presence of RNA (Fig. 2B) (Krypotou et al, 2023). Unlike the strain expressing wild-type *Bt*Rho, strains expressing *Bt*Rho variants that retained the positive charge of KE motif 1 and KE motif 2 (KE motifs 1 + 2 Shuffled, K/R E/D) displayed increased condensation even in the presence of glucose (Fig. 3G). These results, which agree with the results of in vitro condensation (Fig. 3F), indicate that the positive charge and the charge densities in KE motif 1 and KE motif 2 are essential for *Bt*Rho condensation, and that the specific amino acid sequences of these motifs modulate *Bt*Rho phase behavior in response to nutritional signals.

The amino acid sequence of KE motif 3 is not as highly conserved as the sequences of KE motif 1 and KE motif 2 (see Fig. 1C,D). This raised the possibility of the charge within KE motif 3 being more important than its specific amino acid sequence for the regulation of *Bt*Rho condensation. Consistent with this hypothesis, a strain expressing a *Bt*Rho variant with a shuffled KE motif 3 exhibited wild-type *Bt*Rho-like condensation (Fig. 3G). Additionally, a strain expressing the variant with the charged residues of KE motif 3 neutralized displayed high levels of condensation both in glucose and in carbon starvation conditions (Fig. 3G), and these levels were similar to those of the strain expressing wild-type *Bt*Rho during carbon starvation (Fig. 3G). Finally, a strain in which all three KE motifs were shuffled displayed a small decrease in condensation compared to wild-type *Bt*Rho during carbon starvation (Fig. EV5E). Taken together, the results suggest that the charge and sequence of KE motifs 1 and 2 are essential for RNA-dependent *Bt*Rho condensation, whereas the charge of KE motif 3 controls *Bt*Rho condensation.

### A positively charged, conserved motif in the PLD subdomain governs *Bt*Rho condensation in response to carbon starvation

**Figure 4:**
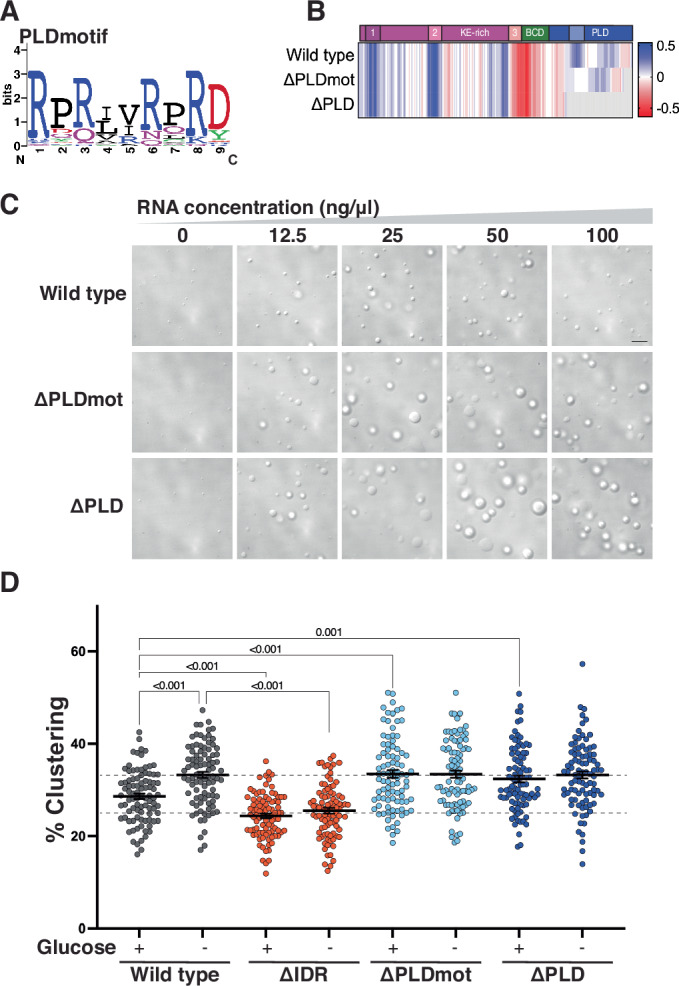
Conserved short motifs within the PLD control *Bt*Rho condensation. (**A**) Sequence logo of the conserved motif within the PLD subdomain indicated in Fig. 1C–E. (**B**) Heatmap of the amino acid charge per residue (EMBOSS (Rice et al, 2000), sliding window 11 amino acids) for the wild-type protein and indicated mutants. The regions corresponding to the KE-rich, BCD, and PLD and the identified conserved motifs are also indicated. (**C**) DIC microscopy of in vitro condensation assays of wild-type *Bt*Rho and indicated variants (2.5 μM) in the presence of increasing RNA amounts corresponding to total RNA extract from *B. thetaiotaomicron* (see also Fig. EV4C). Three independent experiments were performed, and a representative experiment is shown. Scale bar: 5 μm. (**D**) In vivo *Bt*Rho condensation calculated as % clustering in *B. thetaiotaomicron* strains expressing HA-tagged versions of the indicated proteins (wild type: AK82, ΔIDR: AK86, ΔPLDmotif: AK544, and ΔPLD: AK427). Bacteria were grown in glucose (+) until mid-exponential phase and then shifted to a media without any carbon source for 30 min (−). Data points represent clustering values of individual cells from three independent experiments (*n* = 90), black bars are mean values and error bars represent SEM. Dashed lines are used as visual aids to indicate the levels of clustering for wild-type *Bt*Rho in carbon starvation and for the ΔIDR *Bt*Rho. One-way ANOVA was performed, followed by pairwise comparisons between wild-type and mutant variants for the same growth condition or between the two conditions for the same strain. *P* values < 0.05 are shown. Šidak’s test was used to correct for multiple comparisons. Source data are available online for this figure.

We identified a nine residue-long sequence within the PLD subdomain—termed the PLD motif—whose composition and charge are conserved within the *Bacteroidetes* (see Figs. 1C–E and 4A). To explore whether the PLD motif is responsible for the phenotypes exhibited by the variant lacking the entire PLD, we engineered a *Bt*Rho variant lacking the PLD motif (Fig. 4B). Like the *Bt*Rho variant lacking the PLD, the engineered variant formed larger condensates than wild-type *Bt*Rho (Figs. 4C and EV4C). In vivo, the protein lacking the PLD motif showed similar extents of condensation in glucose and carbon starvation, phenocopying the behavior of the protein lacking the entire PLD (Fig. 4D). Thus, the charged PLD motif appears to be the main contributor of the ability of the PLD to modulate *Bt*Rho condensation.

### Computations reveal intradomain interactions among IDR motifs

**Figure 5:**
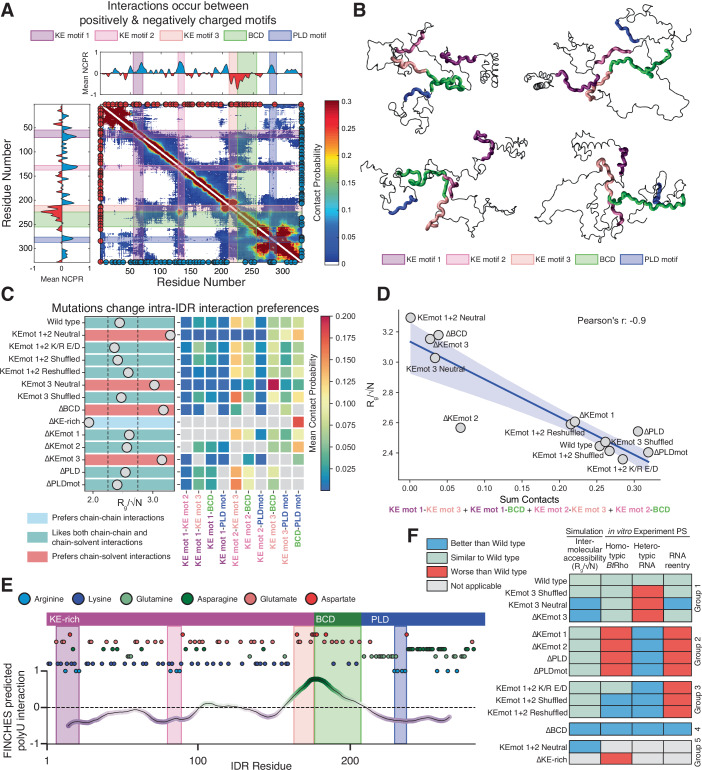
Computational simulations reveal the network of interactions mediated by the IDR of *Bt*Rho. (**A**) Contact map from all atom simulations for wild-type *Bt*Rho N-terminus harboring the IDR. Here, a contact is defined as closest heavy atoms within 15 Å. Plots along the top and left show the mean net charge per residue (NCPR) and highlight regions of enriched charge density. *Bt*Rho IDR motifs are highlighted. Interactions occur between positively- and negatively-charged motifs. (**B**) Representative snapshots extracted from all atom simulations for the wild-type *Bt*Rho N-terminus using VMD (Humphrey et al, 1996). *Bt*Rho IDR motifs are highlighted as indicated at the bottom of the panel. (**C**) Normalized radius of gyration (left) and mean contact probabilities between different IDR motifs (right) for *Bt*Rho N-terminal variants. Please note how mutations in different IDR motifs impact intra-IDR interaction preferences. (**D**) Correlation between the sum of the mean contact probabilities of the high charge density domains and the normalized radius of gyration. Shaded region represents 90% confidence interval. (**E**) Predicted polyU interaction strengths along the wild-type *Bt*Rho IDR-containing N-terminal region using FINCHES (Ginell et al, 2025) and a window size of 31. Here, negative values denote attractive interactions, and positive values denote repulsive interactions. Circles along the top of the panel indicate the positions of various residue types. (**F**) Summary of the relative change for the type of interactions that modulate condensation for all variants examined both experimentally and computationally compared to wild-type *Bt*Rho. Source data are available online for this figure.

To gain molecular-level insights into the homotypic interactions that modulate *Bt*Rho condensation, we performed all atom simulations of monomeric forms of the *Bt*Rho N-terminus harboring the IDR (Vitalis and Pappu, 2009). For the wild type, weak long-range intramolecular interactions were observed between the identified *Bt*Rho IDR motifs. Specifically, positively charged KE motifs 1 and 2 interact with the negatively charged KE motif 3 and the BCD (Fig. 5A,B). The PLD motif also engages in weak interactions with the negatively charged KE motif 3 and the BCD (Fig. 5A,B).

**Figure EV6:**
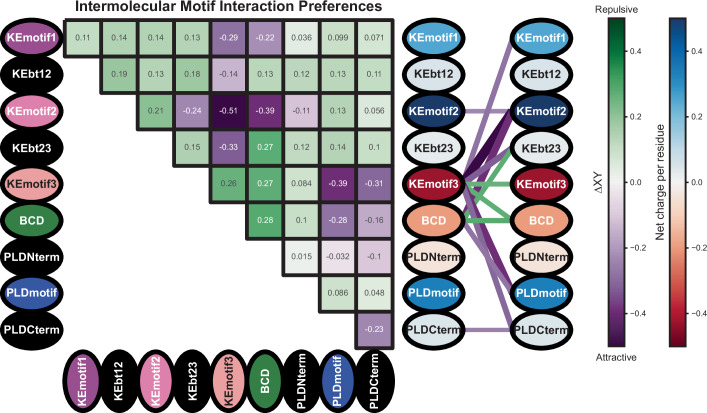
Two molecule simulations reveal intermolecular interactions between the identified motifs in *Bt*Rho. Two molecule simulations were run by splitting up the *Bt*Rho IDR into nine pieces (KE motif 1, KEbt12 (region between motifs 1 + 2), KE motif 2, KEbt23 (region between motifs 2 and 3), KE motif 3, BCD, PLD Nterm, PLDmotif, PLD Cterm) and then determining the effective interaction between each pair of pieces (ΔXY). Negative values imply attraction and positive values imply repulsion. Lines are drawn between two pieces if |ΔXY| ≥ 0.23 and weighted by their interaction strength.

To identify potential intermolecular homotypic *Bt*Rho IDR interactions, we performed simulations for pairs of molecules using two fractions of the sequence at a time. This analysis predicted that the IDR regions that interact intramolecularly can also interact intermolecularly (Fig. EV6).

The interplay among intramolecular *Bt*Rho interactions, intermolecular homotypic *Bt*Rho interactions, and intermolecular heterotypic *Bt*Rho-RNA interactions should contribute to *Bt*Rho condensation (Bremer et al, 2022; Farag et al, 2023; Martin et al, 2020). The observed preferences for intramolecular interactions are likely to be inhibitory of heterotypic intermolecular interactions and contributors to homotypic intermolecular associations (Guillen-Boixet et al, 2020).

We examined the change in intramolecular interactions for different *Bt*Rho IDR variants using two methods (Fig. 5C). In the first method, we extracted global dimensions of the *Bt*Rho N-terminus by calculating the normalized radius of gyration, R_g_/√*N*, where *N* denotes the number of residues in the variant sequence. In the second method, we calculated the mean contact probability between *Bt*Rho IDR motifs. On average, the IDR of wild-type *Bt*Rho adopts conformations whose global dimensions are consistent with preferring both chain-chain and chain-solvent interactions. By contrast, neutralizing KE motifs 1 and 2 or KE motif 3 increased the global dimensions of the *Bt*Rho N-terminus. Similarly, deleting the negatively charged KE motif 3 or the BCD also led to a preference for expanded conformations. The increased global dimensions corresponded with a decrease in mean contact probability between the positively charged KE motifs 1 and 2 and the negatively charged KE motif 3 and the BCD. Additionally, when considering all *Bt*Rho N-terminus variants, we found a strong negative correlation between the sum of the contact probabilities and the global dimensions (Fig. 5D, Pearson’s r = −0.9). These results suggest that interactions between the high charge density motifs drive compaction of the *Bt*Rho N-terminus, which is consistent with well-established findings with other proteins (Das and Pappu, 2013; Shinn et al, 2022). Interactions that drive intrachain compaction are useful proxies for the homotypic interactions that drive condensation (Lin and Chan, 2017; Martin et al, 2020; Pappu et al, 2008).

Next, we asked if the *Bt*Rho motifs are likely to engage in intermolecular heterotypic *Bt*Rho-RNA interactions. To assess this possibility, we plotted the predicted poly-rU interaction score as determined from FINCHES (Ginell et al, 2025) along the *Bt*Rho IDR sequence. Here, negative values imply attractions, whereas positive values imply repulsions. The magnitudes refer to the strengths of the attractions/repulsions. We find that interaction minima correspond to the positively charged KE motif 1, KE motif 2, and the PLD motif (Fig. 5E). By contrast, the negatively charged KE motif 3 and BCD are predicted to have repulsive interactions with RNA (Fig. 5E). These results suggest that some of the same regions that drive *Bt*Rho intramolecular interactions have the highest likelihoods of interacting with RNA.

The relative effects of intramolecular and intermolecular interactions in the wild type and variant proteins examined both computationally and experimentally are summarized in Fig. 5F. We compared the behavior of each variant to the wild type based on: (i) the predictions that the variants would engage in heterotypic interactions (intermolecular accessibility), (ii) the formation of condensates under increasing protein concentration (homotypic *Bt*Rho), (iii) the formation of condensates in the presence of RNA (heterotypic RNA), and (iv) the RNA-dependent reentrant behavior (reentrant phase behavior as RNA concentrations increase). Variants can be categorized into five groups based on their interaction preferences. In group 1, all KE motif 3 variants showed a decrease in RNA-dependent heterotypic interactions compared to the wild type but reentrant behaviors that are similar to or better than the wild type (i.e., the variants form smaller droplets as the RNA concentration increases). Group 2 consists of variants in which a single positively charged motif or the PLD subdomain has been removed. These variants show limited changes in intramolecular interactions but decreased homotypic interactions and enhanced heterotypic interactions with RNA compared to the wild type. Group 3, which comprises KE motif 1 and 2 sequence variants, is similar to Group 2 except that these variants do not diminish the driving forces for *Bt*Rho condensation via homotypic interactions. The variant lacking the BCD constitutes its own group (Group 4) as it leads to expanded conformations as well as increased homotypic and heterotypic intermolecular interactions when compared to the wild type. Finally, a group (Group 5) comprising the ∆KE-rich and KE motif 1 + 2 Neutral variants failed to form condensates either via homotypic interactions or via heterotypic interactions with RNA.

### The alarmone ppGpp is required for *Bt*Rho condensation

When experiencing specific nutritional stresses, bacteria produce the nucleotide alarmones pppGpp and ppGpp, referred to as (p)ppGpp. These molecules regulate fundamental cellular processes, including transcription, protein synthesis, replication, and metabolism (Bange et al, 2021; Hauryliuk et al, 2015). In *B. thetaiotaomicron*, carbon starvation triggers the production of these alarmones (Schofield et al, 2018), and the ability to make and to break down (p)ppGpp is necessary for *B. thetaiotaomicron* fitness in the murine gut (Schofield et al, 2018). We hypothesized that (p)ppGpp is required for *Bt*Rho condensation because *Bt*Rho condensation increases during carbon starvation (Fig. 2) and in the murine gut (Krypotou et al, 2023). To test this hypothesis, we examined *Bt*Rho condensation in wild-type *B. thetaiotaomicron* and an isogenic (p)ppGpp^0^ mutant that lacks the ability to make and to break down (p)ppGpp because it lacks the *BT0700* and *BT3998* genes, which specify sequelogs of the (p)ppGpp synthetase RelA and (p)ppGpp synthetase/hydrolase SpoT (Schofield et al, 2018), respectively.

**Figure 6:**
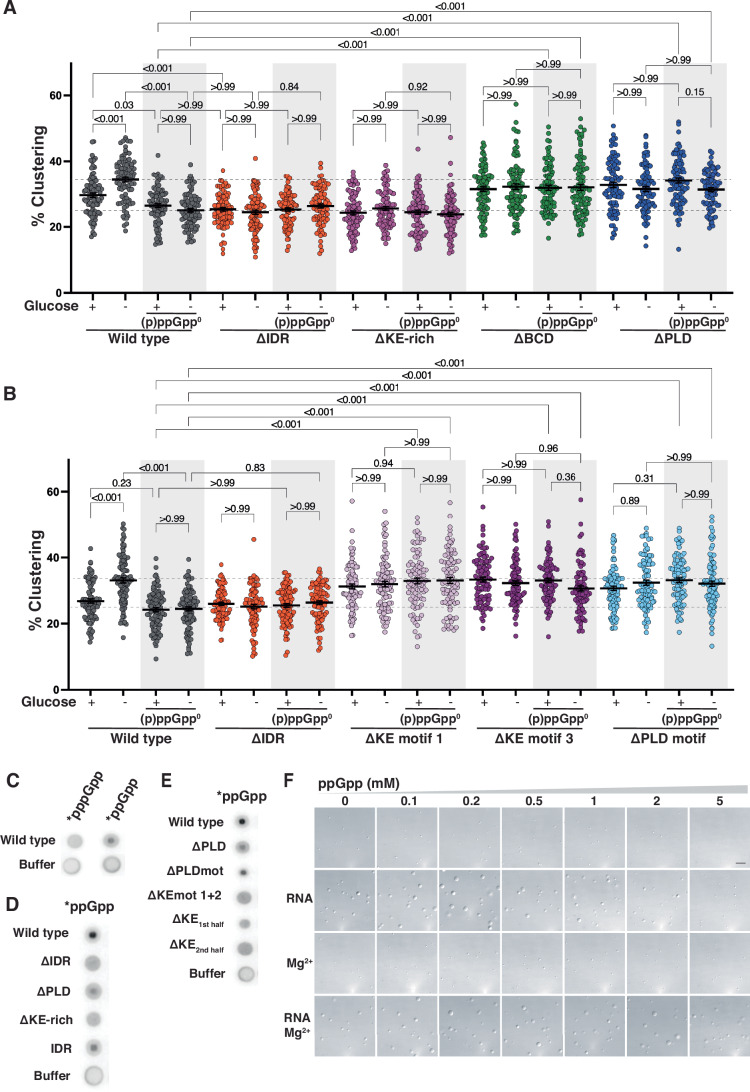
ppGpp controls *Bt*Rho condensation by directly binding to the IDR. (**A**,** B**) In vivo *Bt*Rho condensation calculated as % Clustering in *B. thetaiotaomicron* strains expressing versions of the indicated proteins in isogenic wild-type (wild type: AK82, ΔIDR: AK86, ΔKE-rich: AK408, ΔBCD: AK614, ΔPLD: AK427, ΔKEmotif 1: AK519, ΔΚΕmotif 3: AK582, and ΔPLDmotif: AK544) or (p)ppGpp^0^ (wild type: AK475, ΔIDR: AK384, ΔKE-rich: AK476, ΔBCD: AK684, ΔPLD: AK477, ΔKEmotif 1: AK673, ΔΚΕmotif 3: AK635, and ΔPLDmotif: AK594) backgrounds (gray shaded boxes). Bacteria were grown in glucose (+) until mid-exponential phase and then shifted to a media without any carbon source for 30 min (−). Data points represent clustering values of individual cells from three independent experiments (*n* = 90), black bars are mean values and error bars represent SEM. Dashed lines indicate the levels of clustering for wild-type *Bt*Rho in carbon starvation and for the *ΔIDR Bt*Rho under both conditions. One-way ANOVA was performed, followed by pairwise comparisons. All *P* values are shown for the comparisons performed. Šidak’s test was used to correct for multiple comparisons. (**C**–**E**) DRaCALA assays for wild-type *Bt*Rho and the indicated variants (protein concentration: 10 μM, except IDR 1.5 μM). 0.025 nM *ppGpp and 0.05 *pppGpp were used. Three independent experiments were performed, and representative experiments are shown. All three experiments are shown in Appendix Fig. S1. (**F**) DIC microscopy of in vitro condensation assays of wild-type *Bt*Rho (2.5 μM) in the presence of increasing ppGpp amounts, total RNA extract from *B. thetaiotaomicron* (12.5 ng/μl), and/or MgCl_2_ (2.5 mM) (see also Fig. EV7B). Three independent experiments were performed, and a representative experiment is shown. Scale bar: 5 μm. Source data are available online for this figure.

*Bt*Rho condensation requires (p)ppGpp because the extent of *Bt*Rho condensation in the (p)ppGpp^0^ mutant was similar to that in the Δ*IDR Bt*Rho-expressing strain during both growth on glucose and carbon starvation (Fig. 6A). The extent of condensation remained similarly low in the (p)ppGpp^0^ mutant expressing the variants lacking the IDR or KE-rich subdomain in lieu of wild-type *Bt*Rho (Fig. 6A). By contrast, condensation was high in the (p)ppGpp^0^ mutant expressing variants lacking the BCD or PLD in lieu of the wild-type *Bt*Rho even during growth on glucose (Fig. 6A). Thus, deletion of the BCD or PLD overcomes the condensation defect of the (p)ppGpp^0^ mutant.

Likewise, strains expressing the *Bt*Rho variants with deletions of the KE motif 1, KE motif 3, or the PLD motif exhibited identical behavior in wild-type and (p)ppGpp^0^ backgrounds with similar high extents of condensation in bacteria grown on glucose or facing carbon starvation (Fig. 6B). Again, deletion of highly charged motifs, which are located across the length of the IDR, overcame the condensation defect of the (p)ppGpp^0^ mutant.

### ppGpp binds *Bt*Rho in an IDR-dependent manner

How does (p)ppGpp promote *Bt*Rho condensation? pppGpp and/or ppGpp may function as ligands that bind preferentially to dense phases of *Bt*Rho, promoting condensation via polyphasic linkage (Ruff et al, 2021a, b). This refers to the differential binding of ligands to macromolecules in dense versus dilute phases, where site-specific binding can tilt the balance in favor of or against condensation (Wyman and Gill, 1980). Alternatively, or in addition, (p)ppGpp may promote *Bt*Rho condensation indirectly, by altering the amounts of one or more of the hundreds of mRNAs, the corresponding encoded proteins, and/or various metabolites whose abundance is dramatically altered in a (p)ppGpp^0^ mutant (Schofield et al, 2018), and/or by binding to a target(s) other than  *Bt*Rho. To explore the former possibility, we performed binding assays with purified wild-type *Bt*Rho or variants and radiolabeled pppGpp and ppGpp.

Wild-type *Bt*Rho bound strongly to ppGpp but not to pppGpp (Fig. 6C; Appendix Fig. S1A). Binding was Mg^2+^-dependent because it was detected only in a Mg^2+^-containing buffer and was abolished when EDTA was added to chelate Mg^2+^ (Appendix Fig. S1B,C). Guanosine triphosphate (GTP) outcompeted ppGpp for binding when present in excess, suggesting that GTP can also bind to *Bt*Rho (Appendix Fig. S1C).

ppGpp appears to bind specifically to the IDR of *Bt*Rho because the ∆IDR *Bt*Rho protein failed to bind ppGpp and also because the IDR alone showed strong binding (Fig. 6D; Appendix Fig. S1D). The variant lacking the KE-rich subdomain did not bind ppGpp, behaving like the ∆IDR *Bt*Rho protein (Fig. 6D; Appendix Fig. S1D). By contrast, the variant lacking the PLD subdomain retained strong ppGpp binding, albeit not as strong as the full-length *Bt*Rho or IDR proteins (Fig. 6D; Appendix Fig. S1D). These results suggest that ppGpp binds primarily to the KE-rich subdomain, and to a lesser extent to the PLD subdomain. Preferential binding to the KE-rich subdomain and the ability to bind, albeit differently, to multiple sites, are anticipated to contribute to ligand-mediated enhancement of the driving forces for condensation via polyphasic linkage (Ruff et al, 2021a, b).

That KE motif 1 and KE motif 2 harbor the most charge-dense elements within the KE-rich subdomain (Fig. 1E) suggested their possible involvement in ppGpp binding. As hypothesized, the *Bt*Rho variant lacking KE motif 1 and KE motif 2 failed to bind ppGpp (Fig. 6E; Appendix Fig. S1E), and the same was true for a variant lacking the first 89 residues of the KE-rich subdomain (Fig. 6E; Appendix Fig. S1E), which harbors KE motif 1 and KE motif 2 (Fig. 1D,E). Surprisingly, a variant lacking the last 89 residues of the KE-rich subdomain also failed to bind ppGpp (Fig. 6E; Appendix Fig. S1E), indicating that ppGpp binding requires regions in addition to KE motif 1 and KE motif 2. By contrast, the variant lacking the PLD motif displayed stronger binding than wild-type *Bt*Rho or the variant lacking the PLD subdomain (Fig. 6E; Appendix Fig. S1E), suggesting that the PLD motif interferes with ppGpp binding.

Overall, our results establish that specific regions of the *Bt*Rho IDR bind to ppGpp and that other regions hinder binding, suggesting that ppGpp directly controls *Bt*Rho condensation.

### ppGpp promotes *Bt*Rho condensation in the presence of RNA

**Figure EV7:**
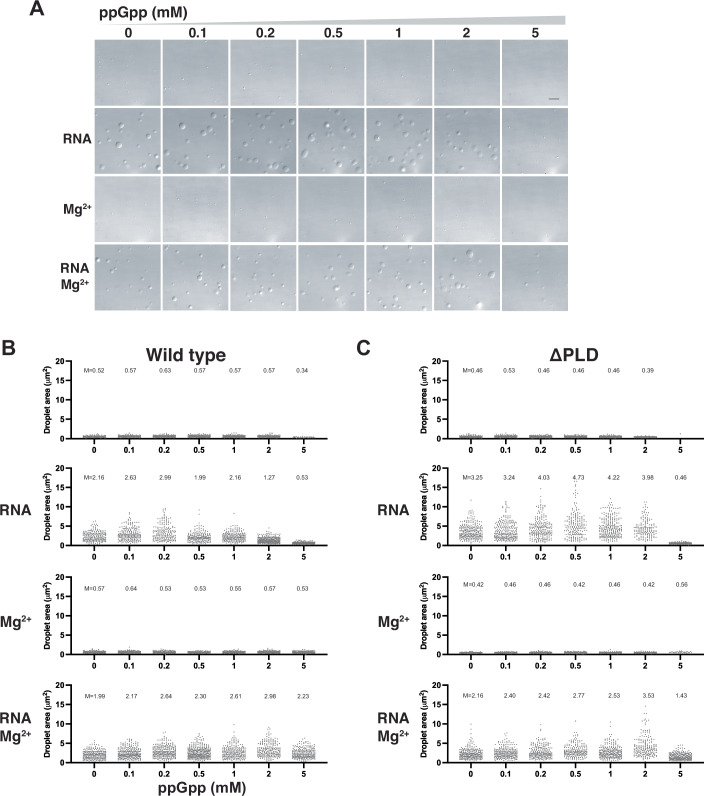
ppGpp promotes RNA-dependent *Bt*Rho phase separation in vitro. (**A**) DIC microscopy of in vitro condensation of the *Bt*Rho ΔPLD protein (2.5 μΜ) in the presence of increasing ppGpp concentration, RNA (12.5 ng/μl), and/or MgCl_2_ (2.5 mM). Three independent experiments were performed, and a representative experiment is shown. Scale bar: 5 μm. (**B**) Size quantification of droplets formed in Fig. 6F for each condition. (**C**) Size quantification of droplets formed in (**A**) for each condition. For each graph, the droplets counted were from three different fields of view of the same sample. The median (M) value is also indicated for each condition.

Having established that (p)ppGpp is required for *Bt*Rho condensation in vivo (Fig. 6A) and that ppGpp binds to the *Bt*Rho IDR in vitro (Fig. 6C,D; Appendix Fig S1D), we investigated whether ppGpp impacts *Bt*Rho condensation in vitro in the presence/absence of RNA and with/without Mg^2+^. When RNA was absent, increasing concentrations of ppGpp had no significant effect on *Bt*Rho condensate formation regardless of the presence of Mg^2+^ (Figs. 6F and EV7B). However, at the highest concentration used (5 mM), ppGpp dissolved the droplets when Mg^2+^ was absent (Figs. 6F and EV7B).

By contrast, when RNA was present, low and intermediate ppGpp concentrations increased the size of the droplets, whereas high ppGpp concentrations decreased the size of the droplets (Figs. 6F and EV7B). However, when Mg^2+^ was also included in the assays, there was a similar increase in the size of the droplets for all tested ppGpp concentrations (Figs. 6F and EV7B).

We explored whether ppGpp impacted condensate formation by the *Bt*Rho variant lacking the PLD subdomain because this variant can form condensates even in a ppGpp^0^ mutant (Fig. 6A). Similarly to wild-type *Bt*Rho, the variant lacking the PLD subdomain showed larger droplets in the presence of ppGpp and RNA, with or without Mg^2+^ (Fig. EV7). The droplets formed by this variant in the presence of RNA differed from those formed by the wild-type *Bt*Rho in that the size of the wild-type droplets decreased starting at 1 mM ppGpp, whereas the size of droplets formed by the variant decreased as the ppGpp concentration increased from 2 mM to 5 mM (Fig. EV7).

A ligand such as ppGpp can bind site-specifically to the macromolecule *Bt*Rho in either the dilute or dense phase. If binding to to these two phases is equivalent, then the ligand will not have any effect on the driving forces for condensation. If the ligand binds preferentially to *Bt*Rho in the dense phase, then condensation is enhanced in the presence of the ligand. The converse would be true if the ligand bound *Bt*Rho preferentially in the dilute phase. Overall, the data in this section suggest that ppGpp functions as a ligand that binds specifically to *Bt*Rho and enhances the driving forces for condensation in the presence of RNA, doing so via preferential binding and concordant with the mechanism of polyphasic linkage (Ruff et al, 2021a, b).

## Discussion

**Figure 7:**
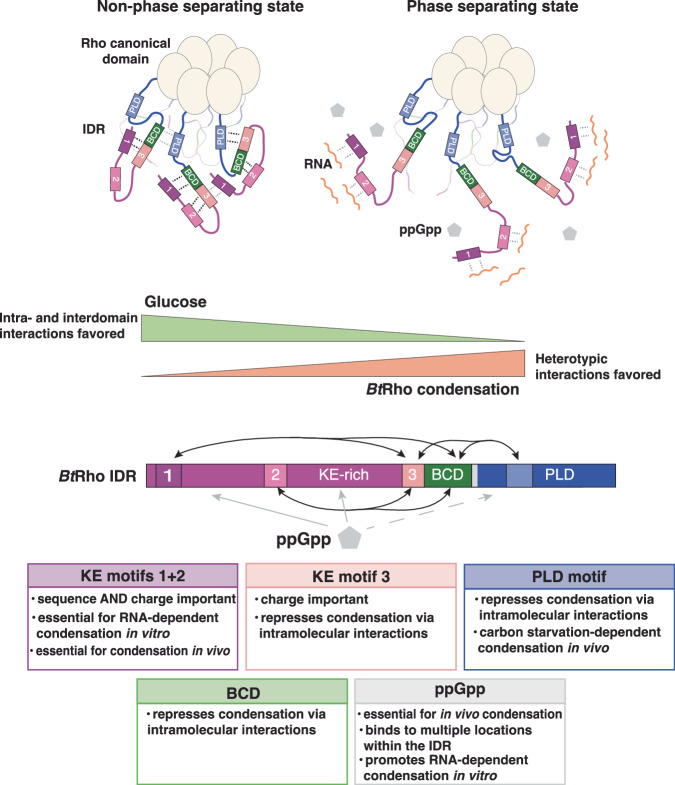
Conserved short motifs in the *Bt*Rho IDR control RNA- and ppGpp-dependent phase separation of *Bt*Rho. Top: Proposed intramolecular (black dashed lines) and intermolecular (gray dashed lines) interactions mediated by the indicated motifs of the *Bt*Rho IDR, as well as their interactions with RNA and/or ppGpp, taking place under the non-phase-separating and phase-separating states. The portion of *Bt*Rho conserved with canonical Rho proteins is depicted as ovals. Six ovals are depicted to indicate that the canonical Rho proteins are hexamers. Middle: There is an inverse correlation between glucose availability and *Bt*Rho condensation. When glucose is abundant intra- and inter-domain interactions among the motifs of the IDR are favored. During carbon starvation, heterotypic interactions with RNA and ppGpp are favored. Bottom: Schematic of the *Bt*Rho IDR. The figure illustrates the various domains and short conserved motifs of the IDR, the homotypic interactions, and the heterotypic interactions with RNA and ppGpp. Strong interactions are in black. Weaker interactions are in gray. Roles of the conserved motifs, *Bacteroides* conserved domain (BCD), and ppGpp in the control of *Bt*Rho condensation.

We established that highly conserved sequence motifs, each 9–30 amino acids long, in the IDR of the essential transcription termination factor *Bt*Rho control its ability to form condensates. The identified motifs are highly charged (Fig. 1), exhibit different physicochemical properties, mediate *Bt*Rho interaction with total RNA extracted from *B. thetaiotaomicron* (Figs. 2–4), and are essential for *Bt*Rho condensation induced by carbon starvation (Figs. 2–4). Computational analysis revealed interactions between different motifs in the *Bt*Rho IDR (Fig. 5). We also determined that the alarmone ppGpp, the amounts of which increase upon carbon starvation (Schofield et al, 2018), a stress condition experienced by the *B. thetaiotaomicron* in the mammalian gut (Groisman et al, 2023), is necessary for condensation in vivo (Fig. 6). ppGpp binds to multiple locations along the IDR of *Bt*Rho (Fig. 6) and promotes *Bt*Rho condensation in the presence of total RNA extracted from *B. thetaiotaomicron* in vitro (Fig. 6). Our findings uncover a conserved molecular grammar whereby short sequences within the IDR mediate interactions with RNA and ppGpp, thus promoting or restricting condensate formation (Fig. 7).

### Highly conserved charged sequences play distinct roles in the phase separation of *Bt*Rho

Protein condensation is often regulated by the concentration of RNA (Maharana et al, 2018). The positively charged KE motif 1 and KE motif 2 are essential for *Bt*Rho interacting with RNA and drive *Bt*Rho condensation in vitro (Fig. 3D) and in vivo (Fig. 3E). KE motif 2 has the highest positive charge density within the KE-rich subdomain (Fig. 1E). Both the charge and sequence of KE motif 1 and KE motif 2 are necessary for *Bt*Rho condensation (Fig. 3F,G). By contrast, the KE motif 3, the BCD, and the PLD motif adjust *Bt*Rho condensation during growth on glucose (i.e., in the absence of the carbon starvation stress signal) (Figs. 2–4). The PLD motif functions as a negative regulator of *Bt*Rho condensation because a variant lacking the PLD motif formed more condensates than the wild-type protein (Fig. 4).

The positively charged KE motif 1, KE motif 2, and PLD subdomain exhibit a high RNA-binding propensity, in contrast to the low propensity of the negatively charged KE motif 3 and BCD. Computations predict long-range interactions between the KE motifs 1 and 2 with KE motif 3 and BCD (Fig. 5), which may hinder the ability of KE motifs 1 and 2 to interact with RNA during growth on glucose. We did not detect RNA-mediated phase separation with the ∆KEBCD variant (Fig. 2B), implying that the PLD by itself is not sufficient for *Bt*Rho phase separation elicited by RNA.

The KE motif 3 and BCD exhibit a high probability for interactions with both KE motif 2 and PLD motif (Fig. 5). These interactions are likely to inhibit condensate formation involving heterotypic interactions between KE motif 2 and RNA. The ΔPLD motif variant displays a stronger drive for condensation than wild-type *Bt*Rho in vivo and in vitro (Fig. 4). The interaction probability between KE motif 2 and KE motif 3 is higher in the ΔPLD motif variant than in the wild-type IDR (Fig. 5). These intramolecular interactions are proxies for the types of homotypic intermolecular interactions that can contribute to condensation in the absence of the competing, modulatory effects of the PLD motif.

### Motif interactions in the IDR drive phase separation

The subdomains of the *Bt*Rho IDR appear to play different roles, some of which are autoinhibitory, and must be overcome for *Bt*Rho condensation to take place. The autoinhibitory interactions are overcome only under certain environmental conditions (i.e., carbon starvation) that trigger the accumulation of specific molecules (i.e., ppGpp). A plausible scenario is that ppGpp binding may promote an open conformation in the *Bt*Rho IDR by disrupting the autoinhibitory interactions between KE motifs 1 and 2 with KE motif 3 and BCD, thereby allowing KE motifs 1 and 2 to interact with RNA and enable condensation (Fig. 7). A similar behavior has been reported for the Ras GTPase-activating protein-binding protein 1 (G3BP1) (Guillen-Boixet et al, 2020; Yang et al, 2020b). This protein harbors three IDRs (one proline-rich region, one positively charged region, and one negatively charged region) that interact with one another to form a compact auto-inhibited structure under non-stress conditions. An increase in the cellular RNA concentration and phosphorylation within the IDR disrupt intradomain interactions, which leads to conformational rearrangements, and allows interactions with RNA and other proteins, resulting in the formation of condensates that mature into stress granules (Guillen-Boixet et al, 2020; Sanders et al, 2020; Yang et al, 2020b).

### Nucleotides and other small molecules control condensates

RNA (Banerjee et al, 2017; Maharana et al, 2018), single-stranded DNA (Kang et al, 2019), ATP (Begovich and Wilhelm, 2020; Kang et al, 2019; Patel et al, 2017; Song, 2021) and other small molecules harboring negatively charged moieties (Babinchak et al, 2020) are known to regulate condensate formation. We have now established that (p)ppGpp is necessary for *Bt*Rho to form condensates in vivo (Fig. 6) and that ppGpp binds to multiple locations within the purified IDR (Fig. 6), with binding to the KE-rich region being the strongest (Fig. 6). Although these results indicate that ppGpp promotes *Bt*Rho condensation directly, likely operating as a multi-site binding ligand, ppGpp (and/or pppGpp) may also impact *Bt*Rho condensation indirectly, via the ability of (p)ppGpp to alter the abundance of RNAs, proteins, and/or metabolites (Schofield et al, 2018) that could bind to *Bt*Rho and/or to a *Bt*Rho-interacting partner participating in condensate formation.

ppGpp increases *Bt*Rho condensate formation in the presence of RNA (Fig. 6) and *Bt*Rho condensate formation increases *Bt*Rho’s transcription termination activity (Krypotou et al, 2023). Therefore, the identified mechanism and physiological outcome of ppGpp binding to the *Bt*Rho protein here described are in stark contrast with those reported for the *E. coli* Rho protein, which was reported to form (p)ppGpp- and ADP-dependent dodecamers and filaments (Wang et al, 2025). This is because (i) filament formation renders the *E. coli* Rho protein inactive (Wang et al, 2025); (ii) pppGpp was found to bind within the canonical ATPase domain of *E. coli* Rho (Wang et al, 2025), but does not bind to the *Bt*Rho protein (Fig. 6); and (iii) ppGpp binding to *Bt*Rho is strictly dependent on the IDR (Fig. 6) and the *E. coli* Rho protein lacks an IDR.

Finally, that 10 of the 56 *E. coli* proteins that bind ppGpp (Wang et al, 2019) harbor predicted IDRs raises the possibility of ppGpp controlling the activity of one or more of these proteins via condensation and for other small molecules abundant in the mammalian gut regulating the formation of condensates in other gut microbes.

### Concluding remarks

Condensation has the potential to alter protein activity rapidly and drastically by modifying the subcellular localization of a protein(s) or compartmentalizing protein(s) in unique environments. This is in contrast to cells responding to internal and/or environmental cues by modifying the synthesis or degradation of a protein(s), the consequences of which take time to manifest. In the case of *Bt*Rho, a combination of short, highly conserved sequences within the IDR dictates how and when condensation takes place. By mediating interactions with RNA, between domains, and with signaling molecules, short amino acid sequences within the essential *Bt*Rho establish a complex regulatory network that alters expression of hundreds of genes and is required for bacterial fitness in the mammalian gut (Krypotou et al, 2023).

## Methods

**Table:** Reagents and tools table

Reagent/Resource	Reference or Source	Identifier or Catalog Number
**Experimental models**		
*Bacteroides thetaiotaomicron strains*, see Appendix Table S1	This study	N/A
*Escherichia coli strains*, see Appendix Table S1	This study	N/A
**Recombinant DNA**		
See Appendix Table S2	This study	N/A
**Antibodies**		
anti-HA rabbit	Millipore Sigma	Cat#H6908
anti-rabbit Alexa Fluor Plus 555	Thermo Fisher Scientific	Cat# A32732
**Oligonucleotides and other sequence-based reagents**		
See Appendix Table S2	Yale Keck Oligonucleotide Synthesis	N/A
**Chemicals, Enzymes and other reagents**		
Dextran	Millipore Sigma	Cat#P5251
FUdR	DOT Scientific	Cat#DSF10705-1
**Software**		
Fiji-ImageJ	Schindelin et al, 2012	N/A
Graphpad Prism	Graphpad	N/A
ABSINTH	Vitalis and Pappu, 2009	N/A
SOURSOP	Lalmansingh et al, 2023	N/A
ALBATROSS	Lotthammer et al, 2024	N/A
FINCHES	Ginell et al, 2025	N/A
Jalview	Waterhouse et al, 2009	N/A

### Bacterial culture conditions

Bacterial strains and plasmids used in this study are listed in Appendix Table S1. *E. coli* strains were grown on solid or liquid Luria Bertani (BD) media supplemented with 100 μg/mL ampicillin (Millipore) at 37 °C. *B. thetaiotaomicron* strains were grown anaerobically at 37 °C in brain heart infusion (Millipore) media containing 5% defibrinated horse blood, tryptone-yeast extract-glucose (TYG) media, or *Bacteroides* minimal media [100 mM KH_2_PO_4_ (pH 7.2), 15 mM NaCl, 8.5 mM (NH_4_SO_4_, 0.5 μg/ml L-cysteine, 1.9 μM hematin, 200 μM L-histidine, 100 μM MgCl_2_, 1.4 μM FeSO_4_, 50 μM CaCl_2_, 1 μg/ml vitamin K_3_, and 5 ng/ml vitamin B_12_, plus glucose (0.5% g/L)].

### DNA manipulations

Plasmid construction was done using the Oligos shown in Appendix Table S2. DNA amplifications were done using GoTaq (Promega) or Q5 High-Fidelity (NEB) DNA polymerases. DNA assembly was done using NEBuilder HiFi DNA (NEB). PCR cleanup and plasmid extractions were done using QIAquick PCR Purification (Qiagen) and QIAprep Spin Miniprep Kit (Qiagen) kits, respectively.

### Construction of *B. thetaiotaomicron* strains expressing *rho* mutant alleles

Plasmids expressing the HA-tagged *Btrho* alleles were created using the Oligos shown in Appendix Table S2 and assembled in *pNBU2-tetQ* or *pKNOCK-tetQ* (Koropatkin et al, 2008*)*. The protein sequence of each mutant variant is shown in Appendix Table S3. *B. thetaiotaomicron* strains expressing only the HA-tagged wild-type or mutant *rho* alleles were constructed using the approaches described below:

(i) For the strains expressing wild-type (AK82), ΔIDR (AK86), ΔKErich (AK408) or ΔPLD (AK427) *rho*, we used the approach previously described (Krypotou et al, 2023). Briefly, in a strain harboring *pEXCHANGE* for deletion of the native *rho, pNBU2* carrying the respective *rho-HA* alleles was introduced at the *att1*. Counterselection on FUdR was used to screen for loss of *pEXCHANGE* and PCR to confirm deletion of the native *rho*.
(ii) A modification of the approach described in (i) was used to make the PCR screening process more efficient by amplifying a shorter region to confirm the *rho* deletion, followed by confirmation of strains expressing the introduced *rho-HA* allele by Western blot. More specifically, a strain harboring *rhoΔIDR* in the native locus (AK245) was used to introduce a C-terminal HA-tag using *pEXCHANGE* (AK502). Counterselection on FUdR and PCR was used to confirm loss of *pEXCHANGE* and C-terminal HA-tagging, resulting in a strain expressing *rhoΔIDR-HA* from the native locus (AK493). This strain was then used for deletion of the *rho* locus, by introducing *pEXCHANGE::Δrho* (GT969), and expression of the *rho* alleles from *pNBU2* attached in *att1*, as done before. The use of the *rhoΔIDR-HA* vs the wild-type *rho* allowed for a shorter PCR amplification using primers binding on either side of the IDR (W3274 and W4760) and confirmation by Western blot due to a shift in the HA-detected protein band, as all constructs were larger than the *Bt*RhoΔIDR protein. This approach was used for the construction of strains expressing: ΔKE motif 1 (AK519), ΔKE motif 2 (AK545), ΔKE motifs 1 + 2 (AK542), ΔKE motif 3 (AK582), ΔKEBCD (AK527), ΔBCDPLD (AK525) and ΔPLD motif (AK544) *rho*.
(iii) For certain strains, we were unable to delete the native *rho* locus using the approaches described above, even after screening hundreds of candidate colonies. For this reason, a different approach based on a single-step direct selection was devised. A *pKNOCK* plasmid was constructed that harbored the *rho* promoter and the first 1808 nt of the *rho* ORF, which includes 800 nt after the end of the IDR, named *pKNOCK-tetQ-rho-replace* (AK598). When introduced into the strain harboring *rhoΔIDR-HA* (AK493), this plasmid integrates in the region upstream or downstream of the IDR deletion site. Depending on the integration site, wild-type or ΔIDR *rho-HA* is expressed. The integration site was confirmed by PCR with primers 10401 (binding within *pKNOCK*) and W5481 (binding within the sequence specifying the HA-tag) and by examining the size of the Rho-HA protein being expressed by Western blot. *pKNOCK* plasmid derivatives harboring the mutations of interest within the IDR were constructed, which would reintroduce the IDR sequence with the desired mutations using oligos shown in Appendix Table S2. This approach proved very efficient and accurate and was used for the construction of strains expressing: wild-type (AK600), ΔIDR (AK602), KE motifs 1 + 2 Neutral (AK610), KE motifs 1 + 2 K/R E/D (AK612), ΔBCD (AK614), ΔKEPLD (AK616), ΔPLD (AK631), KE motifs 1 + 2 Shuffled (AK633), KE motif 3 Neutral (AK654), KE motif 3 Shuffled (AK656), KE motifs 1 + 2 + 3 Neutral (AK658) and KE motifs 1 + 2 + 3 Shuffled (AK660) *rho*. Strains constructed by this method behaved identically to the ones expressing *rho* from *pNBU2* integrated at *att1* in respect to Rho condensation in vivo (Appendix Fig. S2).

### Construction of (p)ppGpp^0^*B. thetaiotaomicron* strains

To construct the (p)ppGpp^0^ strains, we made sequential deletions of the *BT0700* and *BT3998* genes using *pEXCHANGE* vectors (GT1061 and GT1062) carrying the corresponding flanking regions for each gene in strains expressing wild-type or mutant *rho* alleles. Oligos used for the construction of these plasmids are shown in Appendix Table S2. Counterselection on FUdR was used to screen for the loss of *pEXCHANGE* and PCR to confirm the locus deletion using the Oligos shown in Appendix Table S2. In the case of the *rhoΔIDR-HA*, a strain already deleted for *BT0700* and *BT3998* (GT1181) was used to introduce the *pEXCHANGE* for deleting *rho* (GT969), followed by introduction of the *pNBU2-tetQ-rhoΔIDR-HA* (AK74). Deletion of *rho* was confirmed by PCR.

### Construction of *E. coli* strains for overproduction of Rho proteins

For overproduction of wild-type and mutant *Bt*Rho proteins C-terminal His-tagged recombinant proteins were expressed using *pET22b* (Novagene). The *rho* alleles were amplified using the respective oligos shown in Appendix Table S2 and assembled into the NdeI/XhoI digested vector. All cloned plasmids were verified using sequencing and transformed into *E. coli* BL21 (DE3) resulting in the strains shown in Appendix Table S1.

### Purification of recombinant wild-type and mutant *Bt*Rho proteins

Wild-type and mutant *Bt*Rho protein purification was performed as described (Krypotou et al, 2023). Briefly, *E. coli* strains for protein expression and purification were grown in a 1 L LB culture to an OD_600_: 0.6 at 37 °C and after addition of 0.2 mM IPTG, they were incubated at 30 °C for 4 h. Bacteria were collected by centrifugation and resuspended in Lysis buffer [50 mM Tris-HCl pH 7.5, 1 M NaCl, 5% (vol/vol) glycerol, and 0.1 mM DTT, supplemented with cOmplete EDTA-free Protease Inhibitors cocktail (Roche)]. The suspension was sonicated until lysis was clear and the lysate was centrifuged at 11,000 × *g* at 4 °C for 30 min. The supernatant was loaded onto 1 mL NiNTA agarose (Qiagen) previously washed with Lysis buffer and applied on a Polyprep column (Bio-Rad) for gravity flow chromatography. The column was washed with 10 mL lysis buffer+10 mM imidazole. The proteins were eluted with Lysis buffer+200 mM imidazole. 1 mL fractions were collected and analyzed with SDS-PAGE using 4–12% Nupage (Thermo Fisher Scientific) gels and MOPS running buffer (Thermo Fisher Scientific). Gels were stained with GelCode™ Blue Safe Protein Stain (Thermo Fisher Scientific), and fractions containing the purest protein were pooled together. Ultrafiltration (Amicon-Millipore) was used for buffer exchange and to concentrate proteins into a storage buffer (100 mM Tris pH 8, 500 mM KCl, 50% Glycerol, 0.1 mM DTT). Protein samples were aliquoted and stored at −20 °C.

### Total RNA extraction from *B. thetaiotaomicron*

Wild-type *B. thetaiotaomicron* (GT23) was grown in 10 mL minimal media supplemented with glucose until mid-exponential phase and then subjected to carbon starvation for one h by transferring the bacteria to a minimal media without carbon. The bacteria were pelleted, treated with Bacteria RNAprotect (Qiagen), and followed by RNA extraction using the RNeasy kit (Qiagen) according to manufacturer’s instructions. RNA concentration was determined using Nanodrop (Eppendorf), and the RNA was stored at −20 °C.

### In vitro phase separation assays

Assays were performed as described (Krypotou et al, 2023). Briefly, purified proteins used for the assays were switched to a high salt buffer (50 mM Tris pH 8, 500 mM KCl, 2.5% Glycerol, 0.1 mM DTT) and concentrated using ultrafiltration (Amicon-Millipore). Then, a buffer without salt (50 mM Tris pH 8, 2.5% Glycerol, 0.1 mM DTT) was used to dilute the protein at the indicated protein concentration and a salt concentration of 150 mM KCl at 10 μl final volume. Samples were incubated at room temperature for 15 min prior to imaging. For assays in the presence of dextran, dextran was dissolved in the no salt buffer and added to the samples to a final concentration 10% w/v. For phase separation in the presence of RNA, total RNA extract from *B. thetaiotaomicron* was added at the indicated concentrations. For imaging, spacers (SecureSeal™ imaging spacer) were placed on slides, 8 μl of the samples were added to the spaces, covered by coverslips, and left to stand for 5 min before observation to allow the droplets to settle on the coverslip. The samples were observed with a Leica DMi8 inverted microscope, 100x oil lens and DIC filter.

### Immunostaining and fluorescence microscopy of *B. thetaiotaomicron* strains

*B. thetaiotaomicron* immunostaining was performed as described (Krypotou et al, 2023). Briefly, bacteria were grown anaerobically in minimal media supplemented with glucose until reaching mid-exponential phase. 1 mL of culture was briefly centrifuged, and the bacteria were resuspended in pre-reduced fixing buffer (4% PFA in PBS) for the samples corresponding to glucose-grown bacteria and incubated at 37 °C for 20 min, or in minimal media without carbon source for the carbon starvation samples and incubated at 37 °C for 30 min followed by PFA fixation as done for the glucose samples. Bacteria were collected and washed 3 times with PBS before resuspending in 50 μl PBS and storing at 4 °C until use. 20 μl of the fixed bacteria suspension were centrifuged and resuspended in permeabilization buffer (0.1 μg/ml lysozyme in 10 mM EDTA, 50 mM glucose and 20 mM Tris pH 7.5) and incubated at room temperature for 30 min. The suspension of permeabilized bacteria was added on slides precoated with 0.1% w/v poly-lysine and left to air dry. Then, slides were washed three times with PBS, submerged in ice-cold ethanol for 5 min and then in ice-cold acetone for 30 sec. After air drying, samples were rehydrated with PBS and blocked with 2% w/v BSA in PBS at RT for 15 min. After removing the blocking buffer, the primary antibody (anti-HA rabbit, Millipore Sigma, H6908) was added (1:100 dilution) in blocking solution and incubated in a humidity chamber at RT for 1 h. Then, slides were washed three times with PBS-Tween 0.05% v/v and the secondary antibody (anti-rabbit Alexa Fluor Plus 555, Thermo Fisher Scientific, A32732) was added (1:200 dilution) in blocking solution at RT in the dark for 1 h. Samples were then washed three times with PBS-Tween 0.05% v/v, mounted using ProlongGold (Thermo Fisher Scientific), covered with coverslips and let stand at RT overnight before observation. Samples were observed with a Leica DMi8 inverted microscope, 100x oil lens, Lumencor SpectraX as a light source and a Chroma custom filter for detection.

### Microscopy image analysis

Microscopy images were analyzed with Fiji-ImageJ (Schindelin et al, 2012). For calculating *Bt*Rho cellular dispersion as %clustering the method described in Krypotou et al (2023) was used. Briefly, the whole cell area was selected using the bright field image and the fluorescence intensity per pixel was determined using the Histogram function in Fiji-ImageJ (Schindelin et al, 2012). The %clustering was calculated using the number of pixels below a selected threshold (determined as described (Krypotou et al, 2023)) and normalized to the total number of pixels. Increase in the number of foci correlates with increase in clustering values, while bacteria with a uniform signal dispersion show lower clustering values.

### Computational simulations

Atomistic simulations were conducted using the ABSINTH (Vitalis and Pappu, 2009) implicit solvation and forcefield model implemented via the CAMPARI simulation engine (http://campari.sourceforge.net). Parameters were based on the on abs3.2_opls.prm parameter set with the radius of sodium ions increased to enhance sampling of highly acidic regions. Single molecule simulations were performed at 340 K in spherical droplets with radii size of 300 Å with an excess salt concentration of 2.5 mM NaCl. For each *Bt*Rho sequence, five independent simulations were performed with 10^7^ equilibration steps and 5.15 × 10^7^ production steps. In all simulations, the first 49 residues, that are not predicted to be disordered, were held fixed to the AlphaFold (Jumper et al, 2021) Q8A7C9 structure with only side-chain moves allowed.

The SOURSOP (Lalmansingh et al, 2023) analysis package was used to extract contacts from simulations using the get_contact_map() function. Here, the “closest-heavy” mode was used and a distance threshold of 15 Å was utilized in order to account for long-range electrostatic interactions. Mean net charge per residue (NCPR) profiles were calculated by taking the fraction of positive residues minus the fraction of negative residues in window sizes of five residues and then averaging these values for each window the given residue position is a part of. SOURSOP (Lalmansingh et al, 2023) was also utilized to extract radius of gyration values from simulations. We define a window of normalized radius of gyration as being Flory random coil-like between values 2.25 and 3 Å. This window was determined given that normalized Flory random coil simulations yield a length normalized radius of gyration of ~2.5 and ALBATROSS (Lotthammer et al, 2024) sequences with scaling exponents ~0.5 yielded a maximum normalized radius of gyration ~3 for IDRs of length ≥ 100. To predict *Bt*Rho interaction with RNA we deployed the FINCHES (Ginell et al, 2025) plot_protein_nucleic_vector() function using the Mpipi (Joseph et al, 2021) model, a window size of 31, and a smoothing window size of 21.

Two molecule simulations, aimed to assess the potential intermolecular interactions of *Bt*Rho IDR regions, were performed at 340 K in spherical droplets with radii size of 200 Å and an excess of 1.7 mM NaCl. For each two molecule simulation, a harmonic maximum distance restraint of 50 Å was applied between the central Cα atoms of each IDR region. Then, 10 independent full Hamiltonian two molecule simulations were performed with 5 × 10^6^ equilibration steps and 3.5 × 10^7^ production steps. These simulations were compared to reference two molecule simulations that mimic a Flory random coil. Here, just the repulsive Lennard-Jones interaction term is kept on with an exponent of six and a linear scaling factor of 0.001. Ten independent reference Hamiltonian simulations were performed with 10^6^ equilibration steps and 10^7^ production steps.

SOURSOP (Lalmansingh et al, 2023) was utilized to determine the degree of interaction between two IDR regions as referenced to the Flory random coil mimic. Specifically, we used the get_interchain_distance_map() function to calculate the intermolecular distances between all residues of the two molecules for the full and FRC-mimic simulations. Then, ∆XY values were calculated from these distances using the method of Shinn et al (2026), where X and Y denote the two molecules in the simulation. Here, ∆XY values lie between −1 and +1 and quantify the degree of interaction between the two molecules X and Y, with negative values implying the two molecules are attractive and positive values implying the two molecules are repulsive.

### Multiple sequence alignment

For the multiple sequence alignment shown in Fig. 1C the following species and proteins (Uniprot ID in parenthesis) were used to identify the IDRs shown in the figure: *B. thetaiotaomicron* (Q8A7C9), *B. fragilis* (Q5LAX6), *B. ovatus* (A7M2K2), *B. stercoris* (B0NLF9), *B. vulgatus* (A6L4F8), *B. intestinalis* (B3CBN1), *B. dorei* (I9RBX6), *B. nordii* (A0A413VY78), *B. helcogenes* (E6SP34), *B. salanitronis* (F0R3U0), *B. coprocola* (B3JLK9), *B. uniformis* (A7VA38), *B. plebeius* (B5CVX3), *B. oleiciplenus* (K9DXD9), *B. cellulosilyticus* (I9FPU3), *B. caccae* (A5ZIY7), *B. salyersiae* (I9HGG1), *B. xylanisolvens* (I9UPI3), *B. fluxus* (F3PTA2), *B. finegoldii* (K5CKK4), *B. pyogenes* (W4PRQ2), *B. eggerthii* (E5X1G1), *B. massiliensis* (U6RLP9), *B. clarus* (F3PH52), *B. faecis* (A0A1H3B4S9), *B. sartorii* (R9IIM7), *B. heparinolyticus* (A0A2R3MPJ8), *B. caecimuris* (A0A3A9C0F0), *B. acidifaciens* (A0A4S2ADR7) and *B. faecichinchillae* (A0A1M5GB30). The deduced amino acid sequences of the proteins were aligned using Kalign (Lassmann, 2019; Madeira et al, 2024). The alignment was visualized and colored using Jalview (Waterhouse et al, 2009). The alignment part corresponding to the *Bt*Rho IDR sequence was cropped and used for Fig. 1.

### Differential radial capillary action of ligand assay (DRaCALA)

To assess binding of wild-type and mutant *Bt*Rho to (p)ppGpp, differential radial capillary action of ligand assay (DRaCALA) (Roelofs et al, 2011; Yang et al, 2020a) was performed. [5’-α-^32^P]-pppGpp and [5’-α-^32^P]-ppGpp were synthesized from [5’-α-^32^P]-GTP (PerkinElmer) and unlabeled ATP with Rel*Seq*_1-385_ and GppA and subsequently purified with a 1 mL HiTrap QFF column (Cytiva), as described previously (Mechold et al, 2013; Yang et al, 2020a). Purified protein (2.5–10 μM, except 1.5 μM for *Bt*Rho IDR) was mixed with [5’-α-^32^P]-pppGpp (~ 0.05 nM) or [5’-α-^32^P]-ppGpp (~ 0.025 nM) in a reaction buffer containing 10 mM Tris-HCl (pH 7.5), 100 mM NaCl, and 5 mM MgCl_2_, incubated at room temperature for 15 min, and mixed again. 2 μL of each reaction was immediately spotted onto Amersham Protran 0.45 μm nitrocellulose membrane (GE Healthcare) with a replicator pinning tool (V&P Scientific, Inc.). The nitrocellulose membrane was dried, exposed to a phosphor screen overnight, and scanned with a Typhoon FLA9000 phosphorimager (GE Healthcare).

### Figure preparation and statistical analysis

For figure preparation Adobe Illustrator was used. Graphs were prepared using GraphPad Prism (v10, San Diego, California). Statistical analysis was performed using GraphPad Prism. *P* values were obtained from selected pairwise comparisons after one-way ANOVA analysis and Šidak test was used to correct for multiple comparisons. Details on the N number and statistical test used are also mentioned in the figure legends.

## Supplementary information

Appendix

Peer Review File

Source data Fig. 1

Source data Fig. 2

Source data Fig. 3

Source data Fig. 4

Source data Fig. 5

Source data Fig. 6

Expanded View Figures

## Data Availability

All data are available in the main text, or the expanded view materials. All the raw microscopy data have been deposited to Biostudies-BioImages (Accession number S-BIAD2753 https://www.ebi.ac.uk/biostudies/bioimages/studies/S-BIAD2753?key=16f18bcc-b1ef-43a6-a458-f698db45feb5). Raw data from the simulation trajectories are available upon request due to the large size of the files. The source data of this paper are collected in the following database record: biostudies:S-SCDT-10_1038-S44318-026-00793-1.
