# Supplementary material for: Short autoinhibitory sequences control phase separation of an essential bacterial transcription termination factor
Source: EMBO J. 2026 May 11;45(12):4124–52. doi: 10.1038/s44318-026-00793-1 (PMC13269538; doi:10.1038/s44318-026-00793-1)
Supplement: Supplementary file 1 — Appendix [file 44318_2026_793_MOESM1_ESM.pdf]

Appendix for:

Short autoinhibitory sequences control phase separation of an essential bacterial transcription termination factor

Table of contents

Appendix Figure S1 2

Appendix Figure S2 3

Appendix Table S1 4

Appendix Table S2 12

Appendix Table S3 15

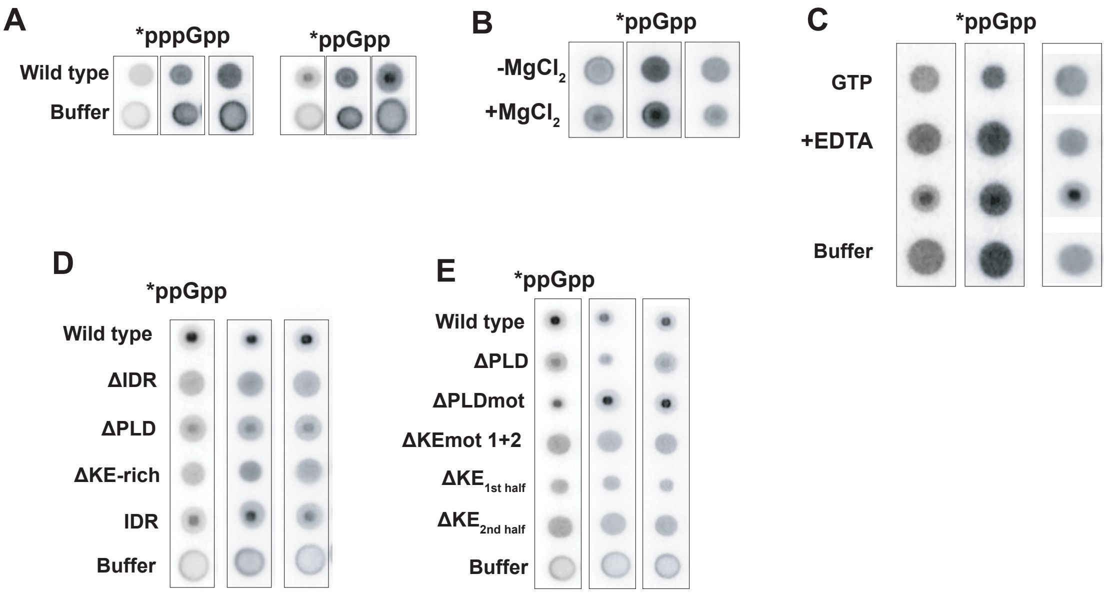

Appendix Figure S1. ppGpp binds to the *Bt*Rho IDR.
(A) All three replicates of the DRaCALA assays shown in **Fig. 6C. (B)** DRaCALA assays for the wild-type *Bt*Rho protein (2.5 µM) with 0.025 nM *ppGpp in the presence of absence of 5 mM $MgCl_2$. The three independent experiments performed are shown. **(C)** DRaCALA assays for the wild-type *Bt*Rho protein (5 µM) with 0.025 nM *ppGpp in the presence of 10 mM EDTA, or 100 µM GTP. **(D)** All three replicates of the DRaCALA assays shown in **Fig. 6D. (E)** All three replicates of the DRaCALA assays shown in **Fig. 6E.** Black boxes indicate each independent experiment and samples are from the same membrane.

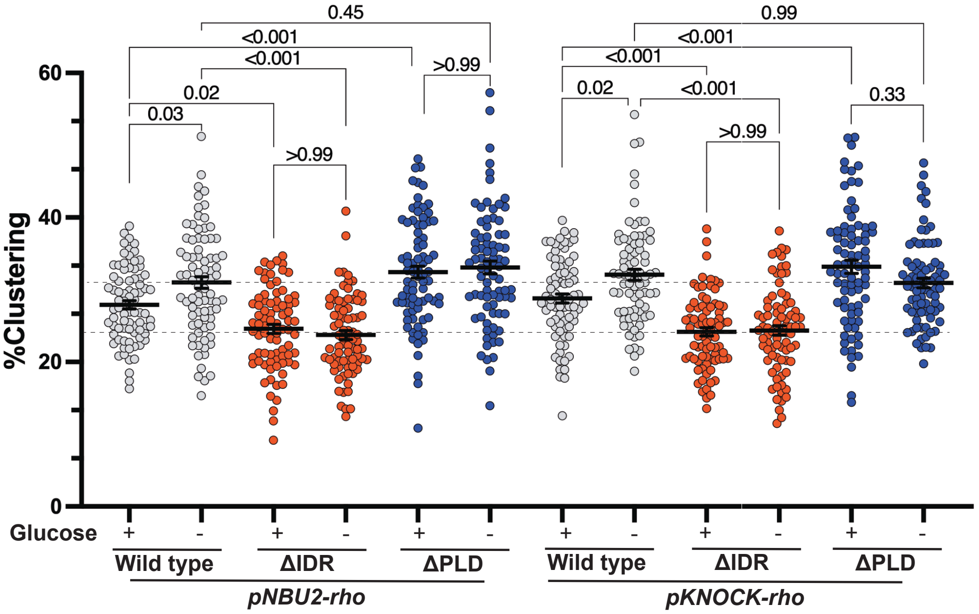

Appendix Figure S2. *Bt*Rho protein clustering behavior is independent of the locus of expression. *In vivo Bt*Rho phase separation calculated as %Clustering in *B. thetaiotaomicron* strains expressing HA-tagged mutant variants from the *pNBU2* integrated in the *att1* locus (wild type: AK82, ΔIDR: AK86, and ΔPLD: AK427) or by reconstructing the *rho* allele in the native *rho* locus using *pKNOCK* (wild type: AK600, ΔIDR: AK602, and ΔPLD: AK631) (see **Methods**). Bacteria were grown in glucose (+) until mid-exponential phase and then shifted to a media without any carbon source for 30 min (-). Data points represent clustering values of individual cells from two independent experiments (n = 80), black bars are mean values and error bars represent SEM. One way ANOVA was performed, followed by pairwise comparisons between wild type and the mutant variants for the same growth condition or between the two conditions for the same strain. P values for pairwise comparisons are shown. Šidak's test was used to correct for multiple comparisons.

Appendix Table S1. Strains and plasmids used in this study.

Identifier	Genotype and use	Reference
Bacteroides thetaiotaomicron VPI-5482		
GT23	Used for genetic manipulations and strain construction.<br>*Δtdk*	PMID: 18611383
GT970	Strain harboring the plasmid to delete the *rho* locus used for introducing *pNBU2* harboring *rho* variants.<br>*Δtdk rho::pEXCHANGE::Δrho*	Krypotou *et al*., 2023
GT1181	Strain unable to produce (p)ppGpp designated as (p)ppGpp^0 background.<br>*Δtdk ΔBT0700 ΔBT3998*	This study.
AK82	Strain expressing *rho-HA* from *att1* and deleted for the *rho* native locus.<br>*Δtdk Δrho pNBU2-tetQ::*P_{rho}*-rho-HA*	Krypotou *et al*., 2023
AK86	Strain expressing *rhoΔIDR-HA* from *att1* and deleted for the *rho* native locus.<br>*Δtdk Δrho pNBU2-tetQ::*P_{rho}*-rhoΔIDR-HA*	Krypotou *et al*., 2023
AK245	Strain deleted for the IDR from the *rho* native locus.<br>*Δtdk rhoΔIDR*	Krypotou *et al*., 2023
AK368	Strain harboring the plasmid to delete *rho* in a (p)ppGpp^0 background.<br>*Δtdk ΔBT0700 ΔBT3998 rho::pEXCHANGE::Δrho*	This study.
AK372	Strain harboring the plasmid to delete *rho* in a (p)ppGpp^0 background and expressing *rhoΔIDR-HA* from *att1.*<br>*Δtdk ΔBT0700 ΔBT3998 rho::pEXCHANGE::Δrho pNBU2-tetQ::*P_{rho}*-rhoΔIDR-HA*	This study.
AK384	Strain expressing *rhoΔIDR-HA* from *att1* in a (p)ppGpp^0 background and deleted for the *rho* native locus.<br>*Δtdk ΔBT0700 ΔBT3998 Δrho pNBU2-tetQ::*P_{rho}*-rhoΔIDR-HA*	This study.
AK405	Strain harboring the plasmid to delete *rho* and expressing *rhoΔKE-rich-HA* from *att1.*<br>*Δtdk rho::pEXCHANGE::Δrho pNBU2-tetQ::*P_{rho}*-rhoΔKE-rich-HA*	This study.
AK407	Strain harboring the plasmid to delete *rho* and expressing *rhoΔPLD-HA* from *att1.*<br>*Δtdk rho::pEXCHANGE::Δrho pNBU2-tetQ::*P_{rho}*-rhoΔPLD-HA*	This study.
AK408	Strain expressing *rhoΔKE-rich-HA* from *att1* and deleted for the *rho* native locus.<br>*Δtdk Δrho pNBU2-tetQ::*P_{rho}*-rhoΔKE-rich-HA*	This study.
AK427	Strain expressing *rhoΔPLD-HA* from *att1* and deleted for the *rho* native locus.<br>*Δtdk Δrho pNBU2-tetQ::*P_{rho}*-rhoΔPLD-HA*	This study.
AK438	Strain expressing *rho-HA* from *att1,* deleted for the *rho* native locus and harboring the plasmid to delete *BT0700*.<br>*Δtdk Δrho BT0701::pEXCHANGE::ΔBT0700 pNBU2-tetQ::*P_{rho}*-rho-HA*	This study.
AK440	Strain expressing *rhoΔKE-rich-HA* from *att1,* deleted for the *rho* native locus and harboring the plasmid to delete *BT0700*.<br>*Δtdk Δrho BT0701::pEXCHANGE::ΔBT0700 pNBU2-tetQ::*P_{rho}*-rhoΔKE-rich-HA*	This study.
AK441	Strain expressing *rhoΔPLD-HA* from *att1,* deleted for the *rho* native locus and harboring the plasmid to delete *BT0700*.<br>*Δtdk Δrho BT0701::pEXCHANGE::ΔBT0700 pNBU2-tetQ::*P_{rho}*-rhoΔPLD-HA*	This study.

AK456	Strain expressing *rho-HA* from *att1,* deleted for the *rho* native locus and *BT0700*.<br>*Δtdk Δrho ΔBT0700 pNBU2-tetQ::P$_{rho}$-rho-HA*	This study.
AK460	Strain expressing *rhoΔKE-rich-HA* from *att1,* deleted for the *rho* native locus and *BT0700*.<br>*Δtdk Δrho ΔBT0700 pNBU2-tetQ::P$_{rho}$-rhoΔKE-rich-HA*	This study.
AK462	Strain expressing *rhoΔPLD-HA* from *att1,* deleted for the *rho* native locus and *BT0700*.<br>*Δtdk Δrho ΔBT0700 pNBU2-tetQ::P$_{rho}$-rhoΔPLD-HA*	This study.
AK472	Strain expressing *rho-HA* from *att1,* deleted for *rho* and *BT0700* and harboring the plasmid to delete *BT3998*.<br>*Δtdk Δrho ΔBT0700 BT3997::pEXCHANGE::ΔBT3998 pNBU2-tetQ::P$_{rho}$-rho-HA*	This study.
AK473	Strain expressing *rhoΔKE-rich-HA* from *att1,* deleted for *rho* and *BT0700* and harboring the plasmid to delete *BT3998*.<br>*Δtdk Δrho ΔBT0700 BT3997::pEXCHANGE::ΔBT3998 pNBU2-tetQ::P$_{rho}$-rhoΔKE-rich-HA*	This study.
AK474	Strain expressing *rhoΔPLD-HA* from *att1,* deleted for *rho* and *BT0700* and harboring the plasmid to delete *BT3998*.<br>*Δtdk Δrho ΔBT0700 BT3997::pEXCHANGE::ΔBT3998 pNBU2-tetQ::P$_{rho}$-rhoΔPLD-HA*	This study.
AK475	Strain expressing *rho-HA* from *att1* in a (p)ppGpp^0 background and deleted for the *rho* native locus.<br>*Δtdk Δrho ΔBT0700 ΔBT3998 pNBU2-tetQ::P$_{rho}$-rho-HA*	This study.
AK476	Strain expressing *rhoΔKE-rich-HA* from *att1* in a (p)ppGpp^0 background and deleted for the *rho* native locus<br>*Δtdk Δrho ΔBT0700 ΔBT3998 pNBU2-tetQ::P$_{rho}$-rhoΔKE-rich-HA*	This study.
AK477	Strain expressing *rhoΔPLD-HA* from *att1* in a (p)ppGpp^0 background and deleted for the *rho* native locus.<br>*Δtdk Δrho ΔBT0700 ΔBT3998 pNBU2-tetQ::P$_{rho}$-rhoΔPLD-HA*	This study.
AK492	Strain harboring a plasmid to add HA-tag at C-term of *rhoΔIDR* in the *rho* locus.<br>*Δtdk rhoΔIDR::pEXCHANGE-rho HA*	This study.
AK493	Strains expressing *rhoΔIDR-HA* from the *rho* locus.<br>*Δtdk rhoΔIDR-HA*	This study.
AK500	Strain expressing *rhoΔIDR-HA* from the *rho* locus and harboring a plasmid for deleting the *rho* locus.<br>*Δtdk rhoΔIDR-HA rho::pEXCHANGE::Δrho*	This study.
AK504	Strain expressing *rhoΔIDR-HA* from the *rho* locus, *rhoΔKEmotif 1-HA* from *att1* and harboring a plasmid for deleting the *rho* locus.<br>*Δtdk rhoΔIDR-HA rho::pEXCHANGE::Δrho pNBU2-tetQ::P$_{rho}$-rhoΔKEmotif 1-HA*	This study.
AK507	Strain expressing *rhoΔIDR-HA* from the *rho* locus, *rhoΔKEmotif 2-HA* from *att1* and harboring a plasmid for deleting the *rho* locus.<br>*Δtdk rhoΔIDR-HA rho::pEXCHANGE::Δrho pNBU2-tetQ::P$_{rho}$-rhoΔKEmotif 2-HA*	This study.
AK508	Strain expressing *rhoΔIDR-HA* from the *rho* locus, *rhoΔKEmotifs 1+2-HA* from *att1* and harboring a plasmid for deleting the *rho* locus.<br>*Δtdk rhoΔIDR-HA rho::pEXCHANGE::Δrho pNBU2-tetQ::P$_{rho}$-rhoΔKEmotifs 1+2-HA*	This study.

AK511	Strain expressing *rhoΔIDR-HA* from the *rho* locus, *rhoΔKEBCD-HA* from *att1* and harboring a plasmid for deleting the *rho* locus.<br>*Δtdk rhoΔIDR-HA rho::pEXCHANGE::Δrho pNBU2-tetQ::*P_{rho}*-rhoΔKEBCD-HA*	This study.
AK512	Strain expressing *rhoΔIDR-HA* from the *rho* locus, *rhoΔBCDPLD-HA* from *att1* and harboring a plasmid for deleting the *rho* locus.<br>*Δtdk rhoΔIDR-HA rho::pEXCHANGE::Δrho pNBU2-tetQ::*P_{rho}*-rhoΔBCDPLD-HA*	This study.
AK519	Strain expressing *rhoΔKEmotif 1-HA* from *att1* and deleted for the *rho* native locus.<br>*Δtdk Δrho pNBU2-tetQ::*P_{rho}*-rhoΔKEmotif 1-HA*	This study.
AK525	Strain expressing *rhoΔBCDPLD-HA* from *att1* and deleted for the *rho* native locus.<br>*Δtdk Δrho pNBU2-tetQ::*P_{rho}*-rhoΔBCDPLD-HA*	This study.
AK527	Strain expressing *rhoΔKEBCD-HA* from *att1* and deleted for the *rho* native locus.<br>*Δtdk Δrho pNBU2-tetQ::*P_{rho}*-rhoΔKEBCD-HA*	This study.
AK533	Strain expressing *rhoΔIDR-HA* from the *rho* locus, *rhoΔPLDmotif-HA* from *att1* and harboring a plasmid for deleting the *rho* locus.<br>*Δtdk rhoΔIDR-HA rho::pEXCHANGE::Δrho pNBU2-tetQ::*P_{rho}*-rhoΔPLDmotif-HA*	This study.
AK542	Strain expressing *rhoΔKEmotifs 1+2-HA* from *att1* and deleted for the *rho* native locus.<br>*Δtdk Δrho pNBU2-tetQ::*P_{rho}*-rhoΔKEmotifs 1+2-HA*	This study.
AK544	Strain expressing *rhoΔPLDmotif-HA* from *att1* and deleted for the *rho* native locus.<br>*Δtdk Δrho pNBU2-tetQ::*P_{rho}*-rhoΔPLDmotif-HA*	This study.
AK545	Strain expressing *rhoΔKEmotif 2-HA* from *att1* and deleted for the *rho* native locus.<br>*Δtdk Δrho pNBU2-tetQ::*P_{rho}*-rhoΔKEmotif 2-HA*	This study.
AK571	Strain expressing *rhoΔPLDmotif-HA* from *att1,* deleted for the *rho* native locus and harboring the plasmid to delete *BT0700*.<br>*Δtdk Δrho BT0701::pEXCHANGE::ΔBT0700 pNBU2-tetQ::*P_{rho}*-rhoΔPLDmotif-HA*	This study.
AK575	Strain expressing *rhoΔPLDmotif-HA* from *att1,* deleted for the *rho* native locus and *BT0700.*<br>*Δtdk Δrho ΔBT0700 pNBU2-tetQ::*P_{rho}*-rhoΔPLDmotif-HA*	This study.
AK580	Strain expressing *rhoΔIDR-HA* from the *rho* locus, *rhoΔKE motif 3-HA* from *att1* and harboring a plasmid for deleting the *rho* locus.<br>*Δtdk rhoΔIDR-HA rho::pEXCHANGE::Δrho pNBU2-tetQ::*P_{rho}*-rhoΔKEmotif 3-HA*	This study.
AK582	Strain expressing *rhoΔKE motif 3-HA* from *att1* and deleted for the *rho* native locus.<br>*Δtdk Δrho pNBU2-tetQ::*P_{rho}*-rhoΔKEmotif 3-HA*	This study.
AK592	Strain expressing *rhoΔPLDmotif-HA* from *att1,* deleted for the *rho* native locus and *BT0700,* and harboring the plasmid to delete *BT3998*.<br>*Δtdk Δrho ΔBT0700 BT3997::pEXCHANGE::ΔBT3998 pNBU2-tetQ::*P_{rho}*-rhoΔPLDmotif-HA*	This study.
AK593	Strain expressing *rhoΔKEmotif 3-HA* from *att1,* deleted for the *rho* native locus and harboring the plasmid to delete *BT0700*.	This study.

	Δtdk Δrho BT0701::pEXCHANGE::ΔBT0700 pNBU2-tetQ::P_{rho}*-rhoΔKEmotif 3-HA*	
AK594	Strain expressing *rhoΔPLDmotif-HA* from *att1* in a (p)ppGpp^0 background and deleted for *rho* native locus.<br>*Δtdk Δrho ΔBT0700 ΔBT3998 pNBU2-tetQ::*P_{rho}*-rhoΔPLDmotif -HA*	This study.
AK596	Strain expressing *rhoΔKEmotif 3-HA* from *att1,* deleted for the *rho* native locus and *BT0700*.<br>*Δtdk Δrho ΔBT0700 pNBU2-tetQ::*P_{rho}*-rhoΔKEmotif 3-HA*	This study.
AK600	Strain expressing wild-type *rho-HA* by reintroducing part of *rho* using *pKNOCK* in a strain harboring *rhoΔIDR-HA* in the native *rho* locus.<br>*Δtdk rhoΔIDR-HA::pKNOCK-tetQ-rho-replace (wild-type rho-HA reconstituted)*	This study.
AK602	Strain expressing *rhoΔIDR-HA* by reintroducing part of *rho* using *pKNOCK* in a strain harboring *rhoΔIDR-HA* in the native *rho* locus.<br>*Δtdk rhoΔIDR-HA::pKNOCK-tetQ-rho-replace (rhoΔIDR-HA reconstituted)*	This study.
AK610	Strain expressing *rhoKE motifs 1+2 Neutral-HA* by reintroducing part of *rho* using *pKNOCK* in a strain harboring *rhoΔIDR-HA* in the native *rho* locus.<br>*Δtdk rhoΔIDR-HA::pKNOCK-tetQ-rhoKE motifs 1+2 Neutral-replace (rhoKE motifs 1+2 Neutral-HA reconstituted)*	This study.
AK612	Strain expressing *rhoKE motifs 1+2 K/R E/D-HA* by reintroducing part of *rho* using *pKNOCK* in a strain harboring *rhoΔIDR-HA* in the native *rho* locus.<br>*Δtdk rhoΔIDR-HA::pKNOCK-tetQ-rhoHA-KE motifs 1+2 K/R E/D-replace (rhoKE motifs 1+2 K/R E/D-HA reconstituted)*	This study.
AK614	Strain expressing *rhoΔBCD-HA* by reintroducing part of *rho* using *pKNOCK* in a strain harboring *rhoΔIDR-HA* in the native *rho* locus.<br>*Δtdk rhoΔIDR-HA::pKNOCK-tetQ-rhoΔBCD-replace (rhoΔBCD-HA reconstituted)*	This study.
AK616	Strain expressing *rhoΔKEPLD-HA* by reintroducing part of *rho* using *pKNOCK* in a strain harboring *rhoΔIDR-HA* in the native *rho* locus.<br>*Δtdk rhoΔIDR-HA::pKNOCK-tetQ-rhoΔKEPLD-replace (rhoΔKEPLD-HA reconstituted)*	This study.
AK619	Strain expressing *rhoΔKEmotif 3-HA* from *att1,* deleted for the *rho* native locus and *BT0700* and harboring the plasmid to delete *BT3998*.<br>*Δtdk Δrho ΔBT0700 BT3997::pEXCHANGE::ΔBT3998 pNBU2-tetQ::*P_{rho}*-rho-ΔKE motif 3-HA*	This study.
AK631	Strain expressing *rhoΔPLD-HA* by reintroducing part of *rho* using *pKNOCK* in a strain harboring *rhoΔIDR-HA* in the native *rho* locus.<br>*Δtdk rhoΔIDR-HA::pKNOCK-tetQ-rhoΔPLD-replace (rhoΔPLD-HA reconstituted)*	This study.
AK633	Strain expressing *rhoKE motifs 1+2 Shuffled-HA* by reintroducing part of *rho* using *pKNOCK* in a strain harboring *rhoΔIDR-HA* in the native *rho* locus.<br>*Δtdk rhoΔIDR-HA::pKNOCK-tetQ-rhoKE motifs 1+2 Shuffled-replace (rhoKE motifs 1+2 Shuffled-HA reconstituted)*	This study.
AK635	Strain expressing *rhoΔKEmotif 3-HA* from *att1* in a (p)ppGpp^0 background and deleted for *rho* native locus.<br>*Δtdk Δrho ΔBT0700 ΔBT3998 pNBU2-tetQ::*P_{rho}*-rho-ΔKEmotif 3-HA*	This study.
AK645	Strain expressing *rhoΔKEmotif 1-HA* from *att1,* deleted for the *rho* native locus and harboring the plasmid to delete *BT0700*.<br>*Δtdk Δrho BT0701::pEXCHANGE::ΔBT0700 pNBU2-tetQ::*P_{rho}*-rhoΔKE motif 1-HA*	This study.

AK649	Strain expressing *rhoΔKEmotif 1-HA* from *att1,* deleted for the *rho* native locus and *BT0700*.<br>*Δtdk Δrho ΔBT0700 pNBU2-tetQ::*P_{rho}*-rhoΔKEmotif 1-HA*	This study.
AK651	Strain expressing *rhoΔKEmotif 1-HA* from *att1,* deleted for the *rho* native locus and *BT0700* and harboring the plasmid to delete *BT3998*.<br>*Δtdk Δrho ΔBT0700 BT3997::pEXCHANGE::ΔBT3998 pNBU2-tetQ::*P_{rho}*-rhoΔKEmotif 1-HA*	This study.
AK654	Strain expressing *rhoKE motif 3 Neutral-HA* by reintroducing part of *rho* using *pKNOCK* in a strain harboring *rhoΔIDR-HA* in the native *rho* locus.<br>*Δtdk rhoΔIDR-HA::pKNOCK-tetQ-rhoKE motif 3 Neutral-replace (rhoKE motif 3 Neutral-HA reconstituted)*	This study.
AK656	Strain expressing *rhoKE motif 3 Shuffled-HA* by reintroducing part of *rho* using *pKNOCK* in a strain harboring *rhoΔIDR-HA* in the native *rho* locus.<br>*Δtdk rhoΔIDR-HA::pKNOCK-tetQ-rhoKE motif 3 Shuffled-replace (rhoKE motif 3 Shuffled-HA reconstituted)*	This study.
AK658	Strain expressing *rhoKE motif 1+2+3 Neutral-HA* by reintroducing part of *rho* using *pKNOCK* in a strain harboring *rhoΔIDR-HA* in the native *rho* locus.<br>*Δtdk rhoΔIDR-HA::pKNOCK-tetQ-rhoKE motifs 1+2+3 Neutral-replace (rhoKE motifs 1+2+3 Neutral-HA reconstituted)*	This study.
AK660	Strain expressing *rhoKE motif 1+2+3 Shuffled-HA* by reintroducing part of *rho* using *pKNOCK* in a strain harboring *rhoΔIDR-HA* in the native *rho* locus.<br>*Δtdk rhoΔIDR-HA::pKNOCK-tetQ-rhoKE motifs 1+2+3 Shuffled-replace (rhoKE motifs 1+2+3 Shuffled-HA reconstituted)*	This study.
AK669	Strain expressing *rhoΔBCD-HA* by reintroducing part of *rho* using *pKNOCK* in a strain harboring *rhoΔIDR-HA* in the native *rho* locus and harboring the plasmid to delete *BT0700*.<br>*Δtdk rhoΔIDR-HA::pKnock-tetQ-rhoΔBCD-replace (rhoΔBCD-HA reconstituted) BT0701::pEXCHANGE::ΔBT0700*	This study.
AK671	Strain expressing *rhoΔBCD-HA* by reintroducing part of *rho* using *pKNOCK* in a strain harboring *rhoΔIDR-HA* in the native *rho* locus and deleted for *BT0700*.<br>*Δtdk rhoΔIDR-HA::pKNOCK-tetQ-rhoΔBCD-replace (rhoΔBCD-HA reconstituted) ΔBT0700*	This study.
AK673	Strain expressing *rhoΔKEmotif 1-HA* from *att1* in a (p)ppGpp^0 background and deleted for *rho* native locus.<br>*Δtdk Δrho ΔBT0700 ΔBT3998 pNBU2-tetQ::*P_{rho}*-rhoΔKEmotif 1-HA*	This study.
AK683	Strain expressing *rhoΔBCD-HA* by reintroducing part of *rho* using *pKNOCK* in a strain harboring *rhoΔIDR-HA* in the native *rho* locus, deleted for *BT0700* and harboring the plasmid to delete *BT3998*.<br>*Δtdk rhoΔIDR-HA::pKNOCK-tetQ-rhoΔBCD-replace (rhoΔBCD-HA reconstituted) ΔBT0700 BT3997::pEXCHANGE::ΔBT3998*	This study.
AK684	Strain expressing *rhoΔBCD-HA* by reintroducing part of *rho* using *pKNOCK* in a strain harboring *rhoΔIDR-HA* in the native *rho* locus in a (p)ppGpp^0 background.<br>*Δtdk rhoΔIDR-HA::pKNOCK-tetQ-rhoΔBCD-replace (rhoΔBCD-HA reconstituted) ΔBT0700 ΔBT3998*	This study.
Escherichia coli DH5α		
	pET22b	Novagen
Escherichia coli BL21(DE3)		

AK692	Overexpression plasmid for wild-type *rho.*<br>*pET22b-rho*	This study.
AK693	Overexpression plasmid for *rhoΔIDR.*<br>*pET22b-rhoΔIDR*	This study.
AK694	Overexpression plasmid for *rhoΔKE-rich.*<br>*pET22b-rhoΔKE-rich*	This study.
AK695	Overexpression plasmid for *rhoΔBCD.*<br>*pET22b-rhoΔBCD*	This study.
AK696	Overexpression plasmid for *rhoΔPLD.*<br>*pET22b-rhoΔPLD*	This study.
AK489	Overexpression plasmid for *rhoΔKEBCD.*<br>*pET22b-rhoΔKEBCD*	This study.
AK490	Overexpression plasmid for *rhoΔKEPLD.*<br>*pET22b-rhoΔKEPLD*	This study.
AK491	Overexpression plasmid for *rhoΔBCDPLD.*<br>*pET22b-rhoΔBCDPLD*	This study.
AK484	Overexpression plasmid for *rhoΔKEmotif 1.*<br>*pET22b-rhoΔKE motif 1*	This study.
AK485	Overexpression plasmid for *rhoΔKEmotif 2.*<br>*pET22b-rhoΔKE motif 2*	This study.
AK686	Overexpression plasmid for *rhoΔKEmotif 3.*<br>*pET22b-rhoΔKE motif 3*	This study.
AK486	Overexpression plasmid for *rhoΔKEmotifs 1+2.*<br>*pET22b-rhoΔKE motifs 1+2*	This study.
AK554	Overexpression plasmid for *rhoKEmotifs 1+2 Neutral.*<br>*pET22b-rhoKE motifs 1+2 Neutral*	This study.
AK553	Overexpression plasmid for *rhoKEmotifs 1+2 Shuffled.*<br>*pET22b-rhoKE motifs 1+2 Shuffled*	This study.
AK555	Overexpression plasmid for *rhoΔPLD motif.*<br>*pET22b-rhoΔPLD motif*	This study.
AK556	Overexpression plasmid for *rhoKEmotifs 1+2 Reshuffled.*<br>*pET22b-rhoKE motifs 1+2 Reshuffled*	This study.
AK557	Overexpression plasmid for *rhoKEmotifs 1+2 K/R E/D.*<br>*pET22b-rhoKE motifs 1+2 K/R E/D*	This study.
AK679	Overexpression plasmid for *rhoKEmotif 3 Neutral.*<br>*pET22b-rhoKE motif 3 Neutral*	This study.
AK681	Overexpression plasmid for *rhoKEmotifs 1+2+3 Neutral.*<br>*pET22b-rhoKEmotifs 1+2+3 Neutral*	This study.
AK680	Overexpression plasmid for *rhoKEmotif 3 Shuffled.*<br>*pET22b-rhoKE motif 3 Shuffled*	This study.
AK682	Overexpression plasmid for *rhoKEmotifs 1+2+3 Shuffled.*<br>*pET22b-rhoKE motifs 1+2+3 Shuffled*	This study.
AK487	Overexpression plasmid for *rhoΔKE(1st 88aa).*<br>*pET22b-rhoΔKE(1-88aa)*	This study.
AK488	Overexpression plasmid for *rhoΔKE(2nd 88aa).*<br>*pET22b-rhoΔKE(89-176aa)*	This study.

AK339	Overexpression plasmid for IDR.<br>*pET22b-IDR*	Krypotou *et al*., 2023
Escherichia coli S17-1 *λ pir* (for conjugation with *B. thetaiotaomicron*)		
GT1061	Plasmid to delete *BT0700.*<br>*pEXCHANGE::ΔBT0700*	This study.
GT1062	Plasmid to delete *BT3998.*<br>*pEXCHANGE::ΔBT3998*	This study.
AK70	Integrative plasmid to express *rho-HA*.<br>*pNBU2-tetQ::P_{rho}-rho-HA*	Krypotou *et al*., 2023
AK74	Integrative plasmid to express *rhoΔIDR-HA*.<br>*pNBU2-tetQ::P_{rho}-rhoΔIDR-HA*	Krypotou *et al*., 2023
AK403	Integrative plasmid to express *rhoΔKE-rich-HA*.<br>*pNBU2-tetQ::P_{rho}-rhoΔKE-rich-HA*	This study.
AK422	Integrative plasmid to express *rhoΔPLD-HA*.<br>*pNBU2-tetQ::P_{rho}-rhoΔPLD-HA*	This study.
AK450	Integrative plasmid to express *rhoΔKEBCD-HA*.<br>*pNBU2-tetQ::P_{rho}-rhoΔKEBCD-HA*	This study.
AK451	Integrative plasmid to express *rhoΔBCDPLD-HA*.<br>*pNBU2-tetQ::P_{rho}-rhoΔBCDPLD-HA*	This study.
AK497	Integrative plasmid to express *rhoΔKE motif 1-HA*.<br>*pNBU2-tetQ::P_{rho}-rhoΔKE motif 1-HA*	This study.
AK498	Integrative plasmid to express *rhoΔKE motif 2-HA*.<br>*pNBU2-tetQ::P_{rho}-rhoΔKE motif 2-HA*	This study.
AK499	Integrative plasmid to express *rhoΔKE motifs 1+2-HA*.<br>*pNBU2-tetQ::P_{rho}-rhoΔKE motifs 1+2-HA*	This study.
AK502	Plasmid to introduce HA-tag at the C-term of *rho* at the native locus.<br>*pEXCHANGE-rho-HA*	This study.
AK538	Integrative plasmid to express *rhoΔPLDmotif-HA*.<br>*pNBU2-tetQ::P_{rho}-rhoΔPLDmotif-HA*	This study.
AK578	Integrative plasmid to express *rhoΔKE motif 3-HA*.<br>*pNBU2-tetQ::P_{rho}-rhoΔKEmotif 3-HA*	This study.
AK598	Plasmid harboring part of *rho* for reconstituting the *rho* locus in a strain harboring *rhoΔIDR-HA* in the native *rho* locus.<br>*pKNOCK-tetQ-rho-replace*	This study.
AK606	Plasmid harboring part of *rho* (including the mutation *KE motifs 1+2 Neutral*) for reconstituting the *rho* locus in a strain harboring *rhoΔIDR-HA* in the native *rho* locus.<br>*pKNOCK-tetQ-rhoKE motifs 1+2 Neutral-replace*	This study.
AK607	Plasmid harboring part of *rho* (including the mutation *KE motifs 1+2 K/R E/D*) for reconstituting the *rho* locus in a strain harboring *rhoΔIDR-HA* in the native *rho* locus.<br>*pKNOCK-tetQ-rhoKE motifs 1+2 K/R E/D-replace*	This study.
AK608	Plasmid harboring part of *rho* (including the mutation *ΔBCD*) for reconstituting the *rho* locus in a strain harboring *rhoΔIDR-HA* in the native *rho* locus.<br>*pKNOCK-tetQ-rhoΔBCD-replace*	This study.

AK609	Plasmid harboring part of *rho* (including the mutation *ΔKEPLD*) for reconstituting the *rho* locus in a strain harboring *rhoΔIDR-HA* in the native *rho* locus.<br>*pKNOCK-tetQ-rhoΔKEPLD-replace*	This study.
AK629	Plasmid harboring part of *rho* (including the mutation *KE motifs 1+2 Shuffled*) for reconstituting the *rho* locus in a strain harboring *rhoΔIDR-HA* in the native *rho* locus.<br>*pKNOCK-tetQ-rhoKE motifs 1+2 Shuffled-replace*	This study.
AK630	Plasmid harboring part of *rho* (including the mutation *ΔPLD*) for reconstituting the *rho* locus in a strain harboring *rhoΔIDR-HA* in the native *rho* locus.<br>*pKNOCK-tetQ-rhoΔPLD-replace*	This study.
AK662	Plasmid harboring part of *rho* (including the mutation *KE motif 3 Neutral*) for reconstituting the *rho* locus in a strain harboring *rhoΔIDR-HA* in the native *rho* locus.<br>*pKNOCK-tetQ-rhoKE motif 3 Neutral-replace*	This study.
AK663	Plasmid harboring part of *rho* (including the mutation *KE motif 3 Shuffled*) for reconstituting the *rho* locus in a strain harboring *rhoΔIDR-HA* in the native *rho* locus.<br>*pKNOCK-tetQ-rhoKE motif 3 Shuffled-replace*	This study.
AK664	Plasmid harboring part of *rho* (including the mutation *KE motifs 1+2+3 Neutral*) for reconstituting the *rho* locus in a strain harboring *rhoΔIDR-HA* in the native *rho* locus.<br>*pKNOCK-tetQ-rhoKE motifs 1+2+3 Neutral-replace*	This study.
AK665	Plasmid harboring part of *rho* (including the mutation *KE motifs 1+2+3 Shuffled*) for reconstituting the *rho* locus in a strain harboring *rhoΔIDR-HA* in the native *rho* locus.<br>*pKNOCK-tetQ-rhoKE motifs 1+2+3 Shuffled-replace*	This study.
GT969	Plasmid to delete *rho.*<br>*pEXCHANGE::Δrho*	Krypotou *et al*., 2023

Appendix Table S2. Oligos used for DNA manipulation and strain construction.

Identifier	Primer use and sequence (5' to 3')
W3263	Forward primer to identify the in-locus *rho* deletion. GGAAGAACGGCTGACTGGGAC
W3264	Reverse primer to identify the in-locus *rho* deletion. TGAATCCGCAGATATTATGCGGG
13277	Forward primer to verify insertion of *pNBU2* in *att1.* CCTTTGCACCGCTTTCAACG
13278	Reverse primer to verify insertion of *pNBU2* in *att1.* TCAACTAAACATGAGATACTAGC
W860	Forward primer to verify insertion of *pNBU2* in *att2.* GGTAACATTATCTAATGAGCGGACA
W861	Reverse primer to verify insertion of *pNBU2* in *att2.* CGGATACCTCCTCTATTTCCAGTTC
W3179	Forward primer to assemble the promoter of *rho* into *pNBU2.* GCTCTAGAACTAGTGGATCCGCTGAAAAAAGTGTTTGATTGTTCA
W3396	Reverse primer for deletion of the *rho* KE-rich subdomain. GGCTCCGGCTATGGCTTGCT
W3397	Forward primer for deletion of the *rho* KE-rich subdomain. AGCAAGCCATAGCCGGAGCCGATGACTTTATCCCAATCGAAGACC
W4763	Reverse primer for end of *rho* carrying the HA-tag to be assembled in *pNBU2.* AAGATAGGCAATTAGTCGACTTAAGCGTAGTCTGGGACGTCGTATGGGTAGCTATTCATGCT CATCAGGAAT
W3190	Reverse primer for deletion of the *rho* BCD subdomain. ATCGTCTTCGCTGGATAAGATGGTA
W3191	Forward primer for deletion of the *rho* BCD subdomain. TCTTATCCAGCGAAGACGATACAGCACAAGCCGCCCCC
W3180	Reverse primer for deletion of the *rho* PLD subdomain. TGTTGTTGTACAGCCGGAAGCTCGGGGGCGGCTTGTGC
W4571	Forward primer for deletion of the *rho* PLD subdomain. CTTCCGGCTGTACAACAACAGCCG
W4557	Forward primer for deletion of the *rho* KE-rich and PLD subdomains. AGCAAGCCATAGCCGGAGCCACAGCACAAGCCGCCCCC
W4558	Forward primer for deletion of the *rho* BCD and PLD subdomains. TACCATCTTATCCAGCGAAGACGATCTTCCGGCTGTACAACAACAG
W5327	Reverse primer for deletion of the *rho* KE motif 2. AGCAGCTTTCTCTGCCTTCG
W5328	Forward primer for deletion of the *rho* KE motif 2. GCAGAGAAAGCTGCTGACGCCAATATCGCTGAAAAAGC
W5329	Reverse primer for deletion of the *rho* PLD motif. TTGTTGCGGTTGTGAATGCTGTTG
W5330	Forward primer for deletion of the *rho* PLD motif. CAACCGCAACAAAACAATAACAACGCCGGCAATAAC
W5471	Forward primer for deletion of the first half (89aa) of the *rho* KE-rich subdomain. CCATAGCCGGAGCCAAAGACGCCAATATCGCTGAAAAAG
W5472	Reverse primer for deletion of the second half (89aa) of the *rho* KE-rich subdomain. GGGATAAAGTCATCGCGGGGACGGCCTACTTTG
W5363	Forward primer for assembly of *rho* into *pET22b.* TAAGAAGGAGATATACATATGTATAACATTATCCAATTGAACGACAAAAATC
W5469	Reverse primer for assembly of *rho* into *pET22b.* GGTGGTGGTGGTGCTCGAG GCTATTCATGCTCATCAGGAATTCG
W5475	Forward primer for deletion of the *rho* KE motif 1. TAAGAAAGTTGCCGCAACAGCCGCACCTAAAAAAGAAGAGAAAG
W5476	Reverse primer for deletion of the *rho* KE motif 1.

	TGCGGCAACTTTCTTAGTGGCT
W5477	Forward primer for creating *pEXCHANGE* to add HA tag at C-term of *rho* in native locus (800bp upstream).<br>GCTCTAGAACTAGTGGATCCCGACCGTGTTCCTTTCGAACAC
W5478	Reverse primer for creating *pEXCHANGE* to add HA tag at C-term of *rho* in native locus (800bp upstream).<br>TTAAGCGTAGTCTGGGACGTCGTATGGGTAGCTATTCATGCTCATCAGGAATTCG
W5479	Forward primer for creating *pEXCHANGE* to add HA tag at C-term of *rho* in native locus (800bp downstream).<br>CCAGACTACGCTTAATCAAGAATAAACGATCATAAATAAGGAACG
W3178	Reverse primer for creating *pEXCHANGE* to add HA tag at C-term of *rho* in native locus (800bp downstream).<br>AAGATAACATTCGAGTCGACGTTCCTACATCCAGGAAACTGTCATT
W5480	Forward primer binding to *rho* for confirmation of HA-tag in the *rho* locus.<br>GAAGGCATCATCCGTCCGC
W5481	Reverse primer binding to HA for confirmation of HA-tag in the *rho* locus.<br>GCGTAGTCTGGGACGTCG
W5482	Reverse primer for creating the *rho* KE motif 1 Shuffled mutation.<br>TTTTCTTTTTACGGTTGCGTTCTAATTTTTCCTCGCGTGCGGCAACTTTCTTAGTGGC
W5483	Forward primer for creating the *rho* KE motif 1 Shuffled mutation.<br>TTAGAACGCAACCGTAAAAAGAAAAAGTCGGATGGAACAGCCGCACCTAAAAAAGAAGAG
W5484	Reverse primer for creating the *rho* KE motif 2 Shuffled mutation.<br>GCGACGTTTGCCGCGAGGCTTAGCAGCTTTCTCTGCCTTCGG
W5485	Forward primer for creating the *rho* KE motif 2 Shuffled mutation.<br>CTCGCGGCAAACGTCGCAAACCCGTAGACGCCAATATCGCTGAAAAAGCAG
W5486	Reverse primer for creating the *rho* KE motif 1 Neutral mutation.<br>GTAGCTCCAGCCGGGGCAGCTGTTAATACAGCTGCGGCAACTTTCTTAGTGGCTCC
W5487	Forward primer for creating the *rho* KE motif 1 Neutral mutation.<br>TGCCCCGGCTGGAGCTACAAACGCTGTATCGAATACAGCCGCACCTAAAAAAGAAGAGAAA<br>G
W5488	Reverse primer for creating the *rho* KE motif 2 Neutral mutation.<br>GGTGCGCCAGCTACAGCAGCAGGAGCAGCTTTCTCTGCCTTCGGTTC
W5489	Forward primer for creating the *rho* KE motif 2 Neutral mutation.<br>GCTGTAGCTGGCGCACCCGCAAAAGACGCCAATATCGCTGAAAAAGCAG
W5490	Reverse primer for creating the *rho* KE motif 1 Reshuffled mutation.<br>TCGCGTAATTTGTTTTCTTTCGAGCGTCCTTTCTCGCGTGCGGCAACTTTCTTAGTGGCTCC<br>G
W5491	Forward primer for creating the *rho* KE motif 1 Reshuffled mutation.<br>CGAAAGAAAACAAATTACGCGATAAAGAAAAGACAGCCGCACCTAAAAAAGAAGAGAAAG
W5492	Reverse primer for creating the *rho* KE motif 2 Reshuffled mutation.<br>CGTTTGGGTACAGGCTTGCGAGCAGCTTTCTCTGCCTTCG
W5493	Forward primer for creating the *rho* KE motif 2 Reshuffled mutation.<br>CAAGCCTGTACCCAAACGCGGCAAACGTGACGCCAATATCGCTGAAAAAGCAG
W5494	Reverse primer for creating the *rho* KE motif 1 K/R E/D mutation.<br>GGTTGCGTTCTCCACGTTTGTCATCACGTAAGCGATCTGCGGCAACTTTCTTAGTGGCTCCG
W5495	Forward primer for creating the *rho* KE motif 1 K/R E/D mutation.<br>CAAACGTGGAGAACGCAACCGTAAGTCGAAGACAGCCGCACCTAAAAAAGAAGAGAAAG
W5496	Reverse primer for creating the *rho* KE motif 2 K/R E/D mutation.<br>GTTTGCCTACGCGCTTGCGAGGAGCAGCTTTCTCTGCCTTCGGTT
W5497	Forward primer for creating the *rho* KE motif 2 K/R E/D mutation.<br>AAGCGCGTAGGCAAACCCAAGCGTGACGCCAATATCGCTGAAAAAGCAG
W5498	Reverse primer used for assembly of the HA tag into *pNBU2.*<br>AAGATAGGCAATTAGTCGACTTAAGCGTAGTCTGGGACGTCGTATGGGTA
W5501	Reverse primer for deletion of the *rho* KE motif 3.<br>CGGTGCCGATTTCTTTTGCG

W5502	Forward primer for deletion of the *rho* KE motif 3.<br>AAGAAATCGGCACCG GATGACTTTATCCCAATCGAAGACC
W5694	Forward primer used to amplify 200bp upstream *rho* and used to create *pKNOCK-tetQ-rho-replace.*<br>GCTCTAGAACTAGTGGATCC CGCTGAAAAAAGTGTTTGATTGTTCA
W5695	Reverse primer used to amplify 1808bp after the *rho* start codon and used to create *pKNOCK-tetQ-rho-replace.*<br>GGCCCCCCCTCGAGGTCGAC CGTTTGGGCTTGTGAAGTGC
W5696	Reverse primer for creating the *rho* KE motif 3 Shuffled mutation.<br>TTTCGTCTTCGGAGTCCACGATACTTTCCGGTGCCGATTTCTTTTGCG
W5697	Forward primer for creating the *rho* KE motif 3 Shuffled mutation.<br>TCGTGGACTCCGAAGACGAAATCGATACCAGCTTAGATGACTTTATCCCAATCGAAGACC
W5698	Reverse primer for creating the *rho* KE motif 3 Neutral mutation.<br>GCTGGATAAGATGGTACTAGCCACAGCGATCACCGGTGCCGATTTCTTTTGCGG
W5699	Forward primer for creating the *rho* KE motif 3 Neutral mutation.<br>AGTACCATCTTATCCAGCGCTGCTGTGGATGACTTTATCCCAATCGAAGACC
W3288	Forward primer for 5' of *BT0700* to delete the in locus allele.<br>GCTCTAGAACTAGTGGATCCTCTTCCACCAGTTTCTTGAAGAGTT
W3289	Reverse primer for 5' of *BT0700* to delete the in locus allele.<br>ATTCAATCTTGTTAATTCGACGTAAAATTA
W3290	Forward primer for 3' of *BT0700* to delete the in locus allele.<br>TCGAATTAACAAGATTGAATGCACTCTATGCTAACAACAAATCTGTTG
W3291	Reverse primer for 3' of *BT0700* to delete the in locus allele.<br>AAGATAACATTCGAGTCGACGGTATCCGGAGTATATGTGCCTTT
W3292	Forward primer to identify the in locus *BT0700* deletion.<br>GTCTACTCCGTAACGTTTGCCG
W3293	Reverse primer to identify the in locus *BT0700* deletion.<br>TATCGGTGATTACCGGAGCA
W3294	Forward primer for 5' of *BT3998* to delete the in locus allele.<br>GCTCTAGAACTAGTGGATCCATGTACGATATTTATGGAGACTGGAATC
W3295	Reverse primer for 5' of *BT3998* to delete the in locus allele.<br>ATTCGTTATCCTTTCATTCGCTT
W3296	Forward primer for 3' of *BT3998* to delete the in locus allele.<br>CGAATGAAAGGATAACGAATTTTATCGTCCGTCCAGTTCTTTC
W3297	Reverse primer for 3' of *BT3998* to delete the in locus allele.<br>AAGATAACATTCGAGTCGACACGGTACGACGAAGTTTCATTC
W3298	Forward primer to identify the in locus *BT3998* deletion.<br>CTGATAGACGAACGCCGCGA
W3299	Reverse primer to identify the in locus BT3998 deletion.<br>CGCAAGACTGCTTCCGGATAC
W3274	Forward primer binding to *rho* and used to confirm the *rho* locus deletion.<br>GATTCTTGATGAGCAAGCCA
W4760	Reverse primer binding to *rho* and used to confirm the *rho* locus deletion.<br>CGATGATAGTCAGCGAACC
10401	Forward primer binding to *pNBU2* and *pKNOCK* used for confirmation of cloning and sequencing.<br>GTGGCGGCCGCTCTAGAACT
13814	Reverse primer binding to *pNBU2* used for confirmation of cloning and sequencing.<br>GTTCCATCACTGGAAGATAGGCAA
10138	Reverse primer binding to *pKNOCK* used for confirmation of cloning and sequencing.<br>ACGCGTCCTCGGTACCGGGC
W4847	Forward primer binding to *pET22b* used for confirmation of cloning and sequencing.<br>CGAAATTAATACGACTCACTATAGGG
W4848	Reverse primer binding to *pET22b* used for confirmation of cloning and sequencing.<br>ATGCTAGTTATTGCTCAGCG

Appendix Table S3. Amino acid sequence of all the *Bt*Rho variants used in this study.

Variant	Sequence
Wild type	MYNIIQLNDKNLSELQVIAKELGIKKADSFKKEELVYKILDEQAIAGATKKVAAEKLKEERKGDKNKRSRTAAPKKEEKVAPAAKNAEVTKNKENAPAAK<br>PQQQPKEEAANKAKEAPVAEPKAEKAAPKRKVGRPRKDANIAEKAENKEVENAKPIVKPTEEKAVAEKTVVAPAAEKATPTQETEKKVKENKPAVAEKPV<br>IAKPQKKSAPVIDEESTILSSEDDDDFIPIEDLPSEKIELPTELFGKFEATKAETAQAAPEQAPQPQQQQHSQPQQRQRIVRPRDNNNNAGNNNVNANNN<br>NNFQRNNNNNQRPPMQQRPAQQQNNVAENLPAVQQQPERKVIEREKPYEFDDILSGVGVLEIMQDGYGFLRSSDYNYLSSPDDIYVSQSQIKLFGLKTGD<br>VVEGIIRPPKEGEKYFPLVKVSKINGRDAAFVRDRVPFEHLTPLFPDEKFRLCKGGYSDSMSARVVDLFAPIGKGQRALIVAQPKTGKTILMKDIANAIA<br>ANHPEVYMIMLLIDERPEEVTDMARSVNAEVIASTFDEPAERHVKIAGIVLEKAKRLVECGHDVVIFLDSITRLARAYNTVSPASGKVLSGGVDANALHK<br>PKRFFGAARNIENGGSLTIIATALIDTGSKMDEVIFEEFKGTGNMELQLDRNLSNKRIFPAVNITASSTRRDDLLLDKTTLDRMWILRKYLADMNPIEAM<br>DFVKDRLEKTRDNDEFLMSMNS
ΔIDR	MYNIIQLNDKNLSELQVIAKELGIKKADSFKKEELVYKILDEQAIAGAELFGVGVLEIMQDGYGFLRSSDYNYLSSPDDIYVSQSQIKLFGLKTGDVVEG<br>IIRPPKEGEKYFPLVKVSKINGRDAAFVRDRVPFEHLTPLFPDEKFRLCKGGYSDSMSARVVDLFAPIGKGQRALIVAQPKTGKTILMKDIANAIAANHP<br>EVYMIMLLIDERPEEVTDMARSVNAEVIASTFDEPAERHVKIAGIVLEKAKRLVECGHDVVIFLDSITRLARAYNTVSPASGKVLSGGVDANALHKPKRF<br>FGAARNIENGGSLTIIATALIDTGSKMDEVIFEEFKGTGNMELQLDRNLSNKRIFPAVNITASSTRRDDLLLDKTTLDRMWILRKYLADMNPIEAMDFVK<br>DRLEKTRDNDEFLMSMNS
ΔKE-rich	MYNIIQLNDKNLSELQVIAKELGIKKADSFKKEELVYKILDEQAIAGADDFIPIEDLPSEKIELPTELFGKFEATKAETAQAAPEQAPQPQQQQHSQPQQ<br>RQRIVRPRDNNNNAGNNNVNANNNNNFQRNNNNNQRPPMQQRPAQQQNNVAENLPAVQQQPERKVIEREKPYEFDDILSGVGVLEIMQDGYGFLRSSDYN<br>YLSSPDDIYVSQSQIKLFGLKTGDVVEGIIRPPKEGEKYFPLVKVSKINGRDAAFVRDRVPFEHLTPLFPDEKFRLCKGGYSDSMSARVVDLFAPIGKGQ<br>RALIVAQPKTGKTILMKDIANAIAANHPEVYMIMLLIDERPEEVTDMARSVNAEVIASTFDEPAERHVKIAGIVLEKAKRLVECGHDVVIFLDSITRLAR<br>AYNTVSPASGKVLSGGVDANALHKPKRFFGAARNIENGGSLTIIATALIDTGSKMDEVIFEEFKGTGNMELQLDRNLSNKRIFPAVNITASSTRRDDLLL<br>DKTTLDRMWILRKYLADMNPIEAMDFVKDRLEKTRDNDEFLMSMNS
ΔBCD	MYNIIQLNDKNLSELQVIAKELGIKKADSFKKEELVYKILDEQAIAGATKKVAAEKLKEERKGDKNKRSRTAAPKKEEKVAPAAKNAEVTKNKENAPAAK<br>PQQQPKEEAANKAKEAPVAEPKAEKAAPKRKVGRPRKDANIAEKAENKEVENAKPIVKPTEEKAVAEKTVVAPAAEKATPTQETEKKVKENKPAVAEKPV<br>IAKPQKKSAPVIDEESTILSSEDDTAQAAPEQAPQPQQQQHSQPQQRQRIVRPRDNNNNAGNNNVNANNNNNFQRNNNNNQRPPMQQRPAQQQNNVAENL<br>PAVQQQPERKVIEREKPYEFDDILSGVGVLEIMQDGYGFLRSSDYNYLSSPDDIYVSQSQIKLFGLKTGDVVEGIIRPPKEGEKYFPLVKVSKINGRDAA<br>FVRDRVPFEHLTPLFPDEKFRLCKGGYSDSMSARVVDLFAPIGKGQRALIVAQPKTGKTILMKDIANAIAANHPEVYMIMLLIDERPEEVTDMARSVNAE<br>VIASTFDEPAERHVKIAGIVLEKAKRLVECGHDVVIFLDSITRLARAYNTVSPASGKVLSGGVDANALHKPKRFFGAARNIENGGSLTIIATALIDTGSK<br>MDEVIFEEFKGTGNMELQLDRNLSNKRIFPAVNITASSTRRDDLLLDKTTLDRMWILRKYLADMNPIEAMDFVKDRLEKTRDNDEFLMSMNS
ΔPLD	MYNIIQLNDKNLSELQVIAKELGIKKADSFKKEELVYKILDEQAIAGATKKVAAEKLKEERKGDKNKRSRTAAPKKEEKVAPAAKNAEVTKNKENAPAAK<br>PQQQPKEEAANKAKEAPVAEPKAEKAAPKRKVGRPRKDANIAEKAENKEVENAKPIVKPTEEKAVAEKTVVAPAAEKATPTQETEKKVKENKPAVAEKPV<br>IAKPQKKSAPVIDEESTILSSEDDDDFIPIEDLPSEKIELPTELFGKFEATKAETAQAAPELPAVQQQPERKVIEREKPYEFDDILSGVGVLEIMQDGYG<br>FLRSSDYNYLSSPDDIYVSQSQIKLFGLKTGDVVEGIIRPPKEGEKYFPLVKVSKINGRDAAFVRDRVPFEHLTPLFPDEKFRLCKGGYSDSMSARVVDL<br>FAPIGKGQRALIVAQPKTGKTILMKDIANAIAANHPEVYMIMLLIDERPEEVTDMARSVNAEVIASTFDEPAERHVKIAGIVLEKAKRLVECGHDVVIFL<br>DSITRLARAYNTVSPASGKVLSGGVDANALHKPKRFFGAARNIENGGSLTIIATALIDTGSKMDEVIFEEFKGTGNMELQLDRNLSNKRIFPAVNITASS<br>TRRDDLLLDKTTLDRMWILRKYLADMNPIEAMDFVKDRLEKTRDNDEFLMSMNS
ΔKEBCD	MYNIIQLNDKNLSELQVIAKELGIKKADSFKKEELVYKILDEQAIAGATAQAAPEQAPQPQQQQHSQPQQRQRIVRPRDNNNNAGNNNVNANNNNNFQRN<br>NNNNQRPPMQQRPAQQQNNVAENLPAVQQQPERKVIEREKPYEFDDILSGVGVLEIMQDGYGFLRSSDYNYLSSPDDIYVSQSQIKLFGLKTGDVVEGII<br>RPPKEGEKYFPLVKVSKINGRDAAFVRDRVPFEHLTPLFPDEKFRLCKGGYSDSMSARVVDLFAPIGKGQRALIVAQPKTGKTILMKDIANAIAANHPEV<br>YMIMLLIDERPEEVTDMARSVNAEVIASTFDEPAERHVKIAGIVLEKAKRLVECGHDVVIFLDSITRLARAYNTVSPASGKVLSGGVDANALHKPKRFFG<br>AARNIENGGSLTIIATALIDTGSKMDEVIFEEFKGTGNMELQLDRNLSNKRIFPAVNITASSTRRDDLLLDKTTLDRMWILRKYLADMNPIEAMDFVKDR<br>LEKTRDNDEFLMSMNS
ΔBCDPLD	MYNIIQLNDKNLSELQVIAKELGIKKADSFKKEELVYKILDEQAIAGATKKVAAEKLKEERKGDKNKRSRTAAPKKEEKVAPAAKNAEVTKNKENAPAAK<br>PQQQPKEEAANKAKEAPVAEPKAEKAAPKRKVGRPRKDANIAEKAENKEVENAKPIVKPTEEKAVAEKTVVAPAAEKATPTQETEKKVKENKPAVAEKPV<br>IAKPQKKSAPVIDEESTILSSEDDLPAVQQQPERKVIEREKPYEFDDILSGVGVLEIMQDGYGFLRSSDYNYLSSPDDIYVSQSQIKLFGLKTGDVVEGI<br>IRPPKEGEKYFPLVKVSKINGRDAAFVRDRVPFEHLTPLFPDEKFRLCKGGYSDSMSARVVDLFAPIGKGQRALIVAQPKTGKTILMKDIANAIAANHPE<br>VYMIMLLIDERPEEVTDMARSVNAEVIASTFDEPAERHVKIAGIVLEKAKRLVECGHDVVIFLDSITRLARAYNTVSPASGKVLSGGVDANALHKPKRFF<br>GAARNIENGGSLTIIATALIDTGSKMDEVIFEEFKGTGNMELQLDRNLSNKRIFPAVNITASSTRRDDLLLDKTTLDRMWILRKYLADMNPIEAMDFVKD<br>RLEKTRDNDEFLMSMNS
ΔKEPLD	MYNIIQLNDKNLSELQVIAKELGIKKADSFKKEELVYKILDEQAIAGADDFIPIEDLPSEKIELPTELFGKFEATKAETAQAAPELPAVQQQPERKVIER<br>EKPYEFDDILSGVGVLEIMQDGYGFLRSSDYNYLSSPDDIYVSQSQIKLFGLKTGDVVEGIIRPPKEGEKYFPLVKVSKINGRDAAFVRDRVPFEHLTPL<br>FPDEKFRLCKGGYSDSMSARVVDLFAPIGKGQRALIVAQPKTGKTILMKDIANAIAANHPEVYMIMLLIDERPEEVTDMARSVNAEVIASTFDEPAERHV<br>KIAGIVLEKAKRLVECGHDVVIFLDSITRLARAYNTVSPASGKVLSGGVDANALHKPKRFFGAARNIENGGSLTIIATALIDTGSKMDEVIFEEFKGTGN<br>MELQLDRNLSNKRIFPAVNITASSTRRDDLLLDKTTLDRMWILRKYLADMNPIEAMDFVKDRLEKTRDNDEFLMSMNS
ΔKE motif 1	MYNIIQLNDKNLSELQVIAKELGIKKADSFKKEELVYKILDEQAIAGATKKVAATAAPKKEEKVAPAAKNAEVTKNKENAPAAKPQQQPKEEAANKAKEA<br>PVAEPKAEKAAPKRKVGRPRKDANIAEKAENKEVENAKPIVKPTEEKAVAEKTVVAPAAEKATPTQETEKKVKENKPAVAEKPVIAKPQKKSAPVIDEES<br>TILSSEDDDDFIPIEDLPSEKIELPTELFGKFEATKAETAQAAPEQAPQPQQQQHSQPQQRQRIVRPRDNNNNAGNNNVNANNNNNFQRNNNNNQRPPMQ<br>QRPAQQQNNVAENLPAVQQQPERKVIEREKPYEFDDILSGVGVLEIMQDGYGFLRSSDYNYLSSPDDIYVSQSQIKLFGLKTGDVVEGIIRPPKEGEKYF<br>PLVKVSKINGRDAAFVRDRVPFEHLTPLFPDEKFRLCKGGYSDSMSARVVDLFAPIGKGQRALIVAQPKTGKTILMKDIANAIAANHPEVYMIMLLIDER<br>PEEVTDMARSVNAEVIASTFDEPAERHVKIAGIVLEKAKRLVECGHDVVIFLDSITRLARAYNTVSPASGKVLSGGVDANALHKPKRFFGAARNIENGGS<br>LTIIATALIDTGSKMDEVIFEEFKGTGNMELQLDRNLSNKRIFPAVNITASSTRRDDLLLDKTTLDRMWILRKYLADMNPIEAMDFVKDRLEKTRDNDEF<br>LMSMNS
ΔKE motif 2	MYNIIQLNDKNLSELQVIAKELGIKKADSFKKEELVYKILDEQAIAGATKKVAAEKLKEERKGDKNKRSRTAAPKKEEKVAPAAKNAEVTKNKENAPAAK<br>PQQQPKEEAANKAKEAPVAEPKAEKAADANIAEKAENKEVENAKPIVKPTEEKAVAEKTVVAPAAEKATPTQETEKKVKENKPAVAEKPVIAKPQKKSAP<br>VIDEESTILSSEDDDDFIPIEDLPSEKIELPTELFGKFEATKAETAQAAPEQAPQPQQQQHSQPQQRQRIVRPRDNNNNAGNNNVNANNNNNFQRNNNNN<br>QRPPMQQRPAQQQNNVAENLPAVQQQPERKVIEREKPYEFDDILSGVGVLEIMQDGYGFLRSSDYNYLSSPDDIYVSQSQIKLFGLKTGDVVEGIIRPPK<br>EGEKYFPLVKVSKINGRDAAFVRDRVPFEHLTPLFPDEKFRLCKGGYSDSMSARVVDLFAPIGKGQRALIVAQPKTGKTILMKDIANAIAANHPEVYMIM<br>LLIDERPEEVTDMARSVNAEVIASTFDEPAERHVKIAGIVLEKAKRLVECGHDVVIFLDSITRLARAYNTVSPASGKVLSGGVDANALHKPKRFFGAARN<br>IENGGSLTIIATALIDTGSKMDEVIFEEFKGTGNMELQLDRNLSNKRIFPAVNITASSTRRDDLLLDKTTLDRMWILRKYLADMNPIEAMDFVKDRLEKT<br>RDNDEFLMSMNS
ΔKE motif 3	MYNIIQLNDKNLSELQVIAKELGIKKADSFKKEELVYKILDEQAIAGATKKVAAEKLKEERKGDKNKRSRTAAPKKEEKVAPAAKNAEVTKNKENAPAAK<br>PQQQPKEEAANKAKEAPVAEPKAEKAAPKRKVGRPRKDANIAEKAENKEVENAKPIVKPTEEKAVAEKTVVAPAAEKATPTQETEKKVKENKPAVAEKPV<br>IAKPQKKSAPDDFIPIEDLPSEKIELPTELFGKFEATKAETAQAAPEQAPQPQQQQHSQPQQRQRIVRPRDNNNNAGNNNVNANNNNNFQRNNNNNQRPP<br>MQQRPAQQQNNVAENLPAVQQQPERKVIEREKPYEFDDILSGVGVLEIMQDGYGFLRSSDYNYLSSPDDIYVSQSQIKLFGLKTGDVVEGIIRPPKEGEK<br>YFPLVKVSKINGRDAAFVRDRVPFEHLTPLFPDEKFRLCKGGYSDSMSARVVDLFAPIGKGQRALIVAQPKTGKTILMKDIANAIAANHPEVYMIMLLID<br>ERPEEVTDMARSVNAEVIASTFDEPAERHVKIAGIVLEKAKRLVECGHDVVIFLDSITRLARAYNTVSPASGKVLSGGVDANALHKPKRFFGAARNIENG<br>GSLTIIATALIDTGSKMDEVIFEEFKGTGNMELQLDRNLSNKRIFPAVNITASSTRRDDLLLDKTTLDRMWILRKYLADMNPIEAMDFVKDRLEKTRDND<br>EFLMSMNS
ΔKE motifs 1+2	MYNIIQLNDKNLSELQVIAKELGIKKADSFKKEELVYKILDEQAIAGATKKVAATAAPKKEEKVAPAAKNAEVTKNKENAPAAKPQQQPKEEAANKAKEA<br>PVAEPKAEKAADANIAEKAENKEVENAKPIVKPTEEKAVAEKTVVAPAAEKATPTQETEKKVKENKPAVAEKPVIAKPQKKSAPVIDEESTILSSEDDDD<br>FIPIEDLPSEKIELPTELFGKFEATKAETAQAAPEQAPQPQQQQHSQPQQRQRIVRPRDNNNNAGNNNVNANNNNNFQRNNNNNQRPPMQQRPAQQQNNV<br>AENLPAVQQQPERKVIEREKPYEFDDILSGVGVLEIMQDGYGFLRSSDYNYLSSPDDIYVSQSQIKLFGLKTGDVVEGIIRPPKEGEKYFPLVKVSKING<br>RDAAFVRDRVPFEHLTPLFPDEKFRLCKGGYSDSMSARVVDLFAPIGKGQRALIVAQPKTGKTILMKDIANAIAANHPEVYMIMLLIDERPEEVTDMARS<br>VNAEVIASTFDEPAERHVKIAGIVLEKAKRLVECGHDVVIFLDSITRLARAYNTVSPASGKVLSGGVDANALHKPKRFFGAARNIENGGSLTIIATALID<br>TGSKMDEVIFEEFKGTGNMELQLDRNLSNKRIFPAVNITASSTRRDDLLLDKTTLDRMWILRKYLADMNPIEAMDFVKDRLEKTRDNDEFLMSMNS

KE motifs 1+2 Neutral	MYNIIQLNDKNLSELQVIAKELGIKKADSFKKEELVYKILDEQAIAGATKKVAAAVLTAAPAGATNAVSNTAAPKKEEKVAPAAKNAEVTKNKENAPAAK<br>PQQQPKEEAANKAKEAPVAEPKAEKAAPAAVAGAPAKDANIAEKAENKEVENAKPIVKPTEEKAVAEKTVVAPAAEKATPTQETEKKVKENKPAVAEKPV<br>IAKPQKKSAPVIDEESTILSSEDDDDFIPIEDLPSEKIELPTELFGKFEATKAETAQAAPEQAPQPQQQQHSQPQQRQRIVRPRDNNNNAGNNNVNANNN<br>NNFQRNNNNNQRPPMQQRPAQQQNNVAENLPAVQQQPERKVIEREKPYEFDDILSGVGVLEIMQDGYGFLRSSDYNYLSSPDDIYVSQSQIKLFGLKTGD<br>VVEGIIRPPKEGEKYFPLVKVSKINGRDAAFVRDRVPFEHLTPLFPDEKFRLCKGGYSDSMSARVVDLFAPIGKGQRALIVAQPKTGKTILMKDIANAIA<br>ANHPEVYMIMLLIDERPEEVTDMARSVNAEVIASTFDEPAERHVKIAGIVLEKAKRLVECGHDVVIFLDSITRLARAYNTVSPASGKVLSGGVDANALHK<br>PKRFFGAARNIENGGSLTIIATALIDTGSKMDEVIFEEFKGTGNMELQLDRNLSNKRIFPAVNITASSTRRDDLLLDKTTLDRMWILRKYLADMNPIEAM<br>DFVKDRLEKTRDNDEFLMSMNS
KE motifs 1+2 Shuffled	MYNIIQLNDKNLSELQVIAKELGIKKADSFKKEELVYKILDEQAIAGATKKVAAREEKLERNRKKKKSDGTAAPKKEEKVAPAAKNAEVTKNKENAPAAK<br>PQQQPKEEAANKAKEAPVAEPKAEKAAKPRGKRRKPVDANIAEKAENKEVENAKPIVKPTEEKAVAEKTVVAPAAEKATPTQETEKKVKENKPAVAEKPV<br>IAKPQKKSAPVIDEESTILSSEDDDDFIPIEDLPSEKIELPTELFGKFEATKAETAQAAPEQAPQPQQQQHSQPQQRQRIVRPRDNNNNAGNNNVNANNN<br>NNFQRNNNNNQRPPMQQRPAQQQNNVAENLPAVQQQPERKVIEREKPYEFDDILSGVGVLEIMQDGYGFLRSSDYNYLSSPDDIYVSQSQIKLFGLKTGD<br>VVEGIIRPPKEGEKYFPLVKVSKINGRDAAFVRDRVPFEHLTPLFPDEKFRLCKGGYSDSMSARVVDLFAPIGKGQRALIVAQPKTGKTILMKDIANAIA<br>ANHPEVYMIMLLIDERPEEVTDMARSVNAEVIASTFDEPAERHVKIAGIVLEKAKRLVECGHDVVIFLDSITRLARAYNTVSPASGKVLSGGVDANALHK<br>PKRFFGAARNIENGGSLTIIATALIDTGSKMDEVIFEEFKGTGNMELQLDRNLSNKRIFPAVNITASSTRRDDLLLDKTTLDRMWILRKYLADMNPIEAM<br>DFVKDRLEKTRDNDEFLMSMNS
KE motifs 1+2 Reshuffled	MYNIIQLNDKNLSELQVIAKELGIKKADSFKKEELVYKILDEQAIAGATKKVAAREEKLERNRKKKKSDGTAAPKKEEKVAPAAKNAEVTKNKENAPAAK<br>PQQQPKEEAANKAKEAPVAEPKAEKAAKPRGKRRKPVDANIAEKAENKEVENAKPIVKPTEEKAVAEKTVVAPAAEKATPTQETEKKVKENKPAVAEKPV<br>IAKPQKKSAPVIDEESTILSSEDDDDFIPIEDLPSEKIELPTELFGKFEATKAETAQAAPEQAPQPQQQQHSQPQQRQRIVRPRDNNNNAGNNNVNANNN<br>NNFQRNNNNNQRPPMQQRPAQQQNNVAENLPAVQQQPERKVIEREKPYEFDDILSGVGVLEIMQDGYGFLRSSDYNYLSSPDDIYVSQSQIKLFGLKTGD<br>VVEGIIRPPKEGEKYFPLVKVSKINGRDAAFVRDRVPFEHLTPLFPDEKFRLCKGGYSDSMSARVVDLFAPIGKGQRALIVAQPKTGKTILMKDIANAIA<br>ANHPEVYMIMLLIDERPEEVTDMARSVNAEVIASTFDEPAERHVKIAGIVLEKAKRLVECGHDVVIFLDSITRLARAYNTVSPASGKVLSGGVDANALHK<br>PKRFFGAARNIENGGSLTIIATALIDTGSKMDEVIFEEFKGTGNMELQLDRNLSNKRIFPAVNITASSTRRDDLLLDKTTLDRMWILRKYLADMNPIEAM<br>DFVKDRLEKTRDNDEFLMSMNS
KE motifs 1+2 K/R E/D	MYNIIQLNDKNLSELQVIAKELGIKKADSFKKEELVYKILDEQAIAGATKKVAADRLRDDKRGERNRKSKTAAPKKEEKVAPAAKNAEVTKNKENAPAAK<br>PQQQPKEEAANKAKEAPVAEPKAEKAAPRKRVGKPKRDANIAEKAENKEVENAKPIVKPTEEKAVAEKTVVAPAAEKATPTQETEKKVKENKPAVAEKPV<br>IAKPQKKSAPVIDEESTILSSEDDDDFIPIEDLPSEKIELPTELFGKFEATKAETAQAAPEQAPQPQQQQHSQPQQRQRIVRPRDNNNNAGNNNVNANNN<br>NNFQRNNNNNQRPPMQQRPAQQQNNVAENLPAVQQQPERKVIEREKPYEFDDILSGVGVLEIMQDGYGFLRSSDYNYLSSPDDIYVSQSQIKLFGLKTGD<br>VVEGIIRPPKEGEKYFPLVKVSKINGRDAAFVRDRVPFEHLTPLFPDEKFRLCKGGYSDSMSARVVDLFAPIGKGQRALIVAQPKTGKTILMKDIANAIA<br>ANHPEVYMIMLLIDERPEEVTDMARSVNAEVIASTFDEPAERHVKIAGIVLEKAKRLVECGHDVVIFLDSITRLARAYNTVSPASGKVLSGGVDANALHK<br>PKRFFGAARNIENGGSLTIIATALIDTGSKMDEVIFEEFKGTGNMELQLDRNLSNKRIFPAVNITASSTRRDDLLLDKTTLDRMWILRKYLADMNPIEAM<br>DFVKDRLEKTRDNDEFLMSMNS
KE motif 3 Neutral	MYNIIQLNDKNLSELQVIAKELGIKKADSFKKEELVYKILDEQAIAGATKKVAAEKLKEERKGDKNKRSRTAAPKKEEKVAPAAKNAEVTKNKENAPAAK<br>PQQQPKEEAANKAKEAPVAEPKAEKAAPKRKVGRPRKDANIAEKAENKEVENAKPIVKPTEEKAVAEKTVVAPAAEKATPTQETEKKVKENKPAVAEKPV<br>IAKPQKKSAPVIAVASTILSSAAVDDFIPIEDLPSEKIELPTELFGKFEATKAETAQAAPEQAPQPQQQQHSQPQQRQRIVRPRDNNNNAGNNNVNANNN<br>NNFQRNNNNNQRPPMQQRPAQQQNNVAENLPAVQQQPERKVIEREKPYEFDDILSGVGVLEIMQDGYGFLRSSDYNYLSSPDDIYVSQSQIKLFGLKTGD<br>VVEGIIRPPKEGEKYFPLVKVSKINGRDAAFVRDRVPFEHLTPLFPDEKFRLCKGGYSDSMSARVVDLFAPIGKGQRALIVAQPKTGKTILMKDIANAIA<br>ANHPEVYMIMLLIDERPEEVTDMARSVNAEVIASTFDEPAERHVKIAGIVLEKAKRLVECGHDVVIFLDSITRLARAYNTVSPASGKVLSGGVDANALHK<br>PKRFFGAARNIENGGSLTIIATALIDTGSKMDEVIFEEFKGTGNMELQLDRNLSNKRIFPAVNITASSTRRDDLLLDKTTLDRMWILRKYLADMNPIEAM<br>DFVKDRLEKTRDNDEFLMSMNS
KE motif 3 Shuffled	MYNIIQLNDKNLSELQVIAKELGIKKADSFKKEELVYKILDEQAIAGATKKVAAEKLKEERKGDKNKRSRTAAPKKEEKVAPAAKNAEVTKNKENAPAAK<br>PQQQPKEEAANKAKEAPVAEPKAEKAAPKRKVGRPRKDANIAEKAENKEVENAKPIVKPTEEKAVAEKTVVAPAAEKATPTQETEKKVKENKPAVAEKPV<br>IAKPQKKSAPESIVDSEDEIDTSLDDFIPIEDLPSEKIELPTELFGKFEATKAETAQAAPEQAPQPQQQQHSQPQQRQRIVRPRDNNNNAGNNNVNANNN<br>NNFQRNNNNNQRPPMQQRPAQQQNNVAENLPAVQQQPERKVIEREKPYEFDDILSGVGVLEIMQDGYGFLRSSDYNYLSSPDDIYVSQSQIKLFGLKTGD<br>VVEGIIRPPKEGEKYFPLVKVSKINGRDAAFVRDRVPFEHLTPLFPDEKFRLCKGGYSDSMSARVVDLFAPIGKGQRALIVAQPKTGKTILMKDIANAIA<br>ANHPEVYMIMLLIDERPEEVTDMARSVNAEVIASTFDEPAERHVKIAGIVLEKAKRLVECGHDVVIFLDSITRLARAYNTVSPASGKVLSGGVDANALHK<br>PKRFFGAARNIENGGSLTIIATALIDTGSKMDEVIFEEFKGTGNMELQLDRNLSNKRIFPAVNITASSTRRDDLLLDKTTLDRMWILRKYLADMNPIEAM<br>DFVKDRLEKTRDNDEFLMSMNS
KE motifs 1+2+3 Neutral	MYNIIQLNDKNLSELQVIAKELGIKKADSFKKEELVYKILDEQAIAGATKKVAAAVLTAAPAGATNAVSNTAAPKKEEKVAPAAKNAEVTKNKENAPAAK<br>PQQQPKEEAANKAKEAPVAEPKAEKAAPAAVAGAPAKDANIAEKAENKEVENAKPIVKPTEEKAVAEKTVVAPAAEKATPTQETEKKVKENKPAVAEKPV<br>IAKPQKKSAPVIAVASTILSSAAVDDFIPIEDLPSEKIELPTELFGKFEATKAETAQAAPEQAPQPQQQQHSQPQQRQRIVRPRDNNNNAGNNNVNANNN<br>NNFQRNNNNNQRPPMQQRPAQQQNNVAENLPAVQQQPERKVIEREKPYEFDDILSGVGVLEIMQDGYGFLRSSDYNYLSSPDDIYVSQSQIKLFGLKTGD<br>VVEGIIRPPKEGEKYFPLVKVSKINGRDAAFVRDRVPFEHLTPLFPDEKFRLCKGGYSDSMSARVVDLFAPIGKGQRALIVAQPKTGKTILMKDIANAIA<br>ANHPEVYMIMLLIDERPEEVTDMARSVNAEVIASTFDEPAERHVKIAGIVLEKAKRLVECGHDVVIFLDSITRLARAYNTVSPASGKVLSGGVDANALHK<br>PKRFFGAARNIENGGSLTIIATALIDTGSKMDEVIFEEFKGTGNMELQLDRNLSNKRIFPAVNITASSTRRDDLLLDKTTLDRMWILRKYLADMNPIEAM<br>DFVKDRLEKTRDNDEFLMSMNS
KE motifs 1+2+3 Shuffled	MYNIIQLNDKNLSELQVIAKELGIKKADSFKKEELVYKILDEQAIAGATKKVAAREEKLERNRKKKKSDGTAAPKKEEKVAPAAKNAEVTKNKENAPAAK<br>PQQQPKEEAANKAKEAPVAEPKAEKAAKPRGKRRKPVDANIAEKAENKEVENAKPIVKPTEEKAVAEKTVVAPAAEKATPTQETEKKVKENKPAVAEKPV<br>IAKPQKKSAPESIVDSEDEIDTSLDDFIPIEDLPSEKIELPTELFGKFEATKAETAQAAPEQAPQPQQQQHSQPQQRQRIVRPRDNNNNAGNNNVNANNN<br>NNFQRNNNNNQRPPMQQRPAQQQNNVAENLPAVQQQPERKVIEREKPYEFDDILSGVGVLEIMQDGYGFLRSSDYNYLSSPDDIYVSQSQIKLFGLKTGD<br>VVEGIIRPPKEGEKYFPLVKVSKINGRDAAFVRDRVPFEHLTPLFPDEKFRLCKGGYSDSMSARVVDLFAPIGKGQRALIVAQPKTGKTILMKDIANAIA<br>ANHPEVYMIMLLIDERPEEVTDMARSVNAEVIASTFDEPAERHVKIAGIVLEKAKRLVECGHDVVIFLDSITRLARAYNTVSPASGKVLSGGVDANALHK<br>PKRFFGAARNIENGGSLTIIATALIDTGSKMDEVIFEEFKGTGNMELQLDRNLSNKRIFPAVNITASSTRRDDLLLDKTTLDRMWILRKYLADMNPIEAM<br>DFVKDRLEKTRDNDEFLMSMNS
ΔPLDmotif	MYNIIQLNDKNLSELQVIAKELGIKKADSFKKEELVYKILDEQAIAGATKKVAAEKLKEERKGDKNKRSRTAAPKKEEKVAPAAKNAEVTKNKENAPAAK<br>PQQQPKEEAANKAKEAPVAEPKAEKAAPKRKVGRPRKDANIAEKAENKEVENAKPIVKPTEEKAVAEKTVVAPAAEKATPTQETEKKVKENKPAVAEKPV<br>IAKPQKKSAPVIDEESTILSSEDDDDFIPIEDLPSEKIELPTELFGKFEATKAETAQAAPEQAPQPQQQQHSQPQQNNNNAGNNNVNANNNNNFQRNNNN<br>NQRPPMQQRPAQQQNNVAENLPAVQQQPERKVIEREKPYEFDDILSGVGVLEIMQDGYGFLRSSDYNYLSSPDDIYVSQSQIKLFGLKTGDVVEGIIRPP<br>KEGEKYFPLVKVSKINGRDAAFVRDRVPFEHLTPLFPDEKFRLCKGGYSDSMSARVVDLFAPIGKGQRALIVAQPKTGKTILMKDIANAIAANHPEVYMI<br>MLLIDERPEEVTDMARSVNAEVIASTFDEPAERHVKIAGIVLEKAKRLVECGHDVVIFLDSITRLARAYNTVSPASGKVLSGGVDANALHKPKRFFGAAR<br>NIENGGSLTIIATALIDTGSKMDEVIFEEFKGTGNMELQLDRNLSNKRIFPAVNITASSTRRDDLLLDKTTLDRMWILRKYLADMNPIEAMDFVKDRLEK<br>TRDNDEFLMSMNS
ΔKE$_{\text{1st half}}$	MYNIIQLNDKNLSELQVIAKELGIKKADSFKKEELVYKILDEQAIAGAKDANIAEKAENKEVENAKPIVKPTEEKAVAEKTVVAPAAEKATPTQETEKKV<br>KENKPAVAEKPVIAKPQKKSAPVIDEESTILSSEDDDDFIPIEDLPSEKIELPTELFGKFEATKAETAQAAPEQAPQPQQQQHSQPQQRQRIVRPRDNNN<br>NAGNNNVNANNNNNFQRNNNNNQRPPMQQRPAQQQNNVAENLPAVQQQPERKVIEREKPYEFDDILSGVGVLEIMQDGYGFLRSSDYNYLSSPDDIYVSQ<br>SQIKLFGLKTGDVVEGIIRPPKEGEKYFPLVKVSKINGRDAAFVRDRVPFEHLTPLFPDEKFRLCKGGYSDSMSARVVDLFAPIGKGQRALIVAQPKTGK<br>TILMKDIANAIAANHPEVYMIMLLIDERPEEVTDMARSVNAEVIASTFDEPAERHVKIAGIVLEKAKRLVECGHDVVIFLDSITRLARAYNTVSPASGKV<br>LSGGVDANALHKPKRFFGAARNIENGGSLTIIATALIDTGSKMDEVIFEEFKGTGNMELQLDRNLSNKRIFPAVNITASSTRRDDLLLDKTTLDRMWILR<br>KYLADMNPIEAMDFVKDRLEKTRDNDEFLMSMNS
ΔKE$_{\text{2nd half}}$	MYNIIQLNDKNLSELQVIAKELGIKKADSFKKEELVYKILDEQAIAGATKKVAAEKLKEERKGDKNKRSRTAAPKKEEKVAPAAKNAEVTKNKENAPAAK<br>PQQQPKEEAANKAKEAPVAEPKAEKAAPKRKVGRPRDDFIPIEDLPSEKIELPTELFGKFEATKAETAQAAPEQAPQPQQQQHSQPQQRQRIVRPRDNNN<br>NAGNNNVNANNNNNFQRNNNNNQRPPMQQRPAQQQNNVAENLPAVQQQPERKVIEREKPYEFDDILSGVGVLEIMQDGYGFLRSSDYNYLSSPDDIYVSQ<br>SQIKLFGLKTGDVVEGIIRPPKEGEKYFPLVKVSKINGRDAAFVRDRVPFEHLTPLFPDEKFRLCKGGYSDSMSARVVDLFAPIGKGQRALIVAQPKTGK<br>TILMKDIANAIAANHPEVYMIMLLIDERPEEVTDMARSVNAEVIASTFDEPAERHVKIAGIVLEKAKRLVECGHDVVIFLDSITRLARAYNTVSPASGKV<br>LSGGVDANALHKPKRFFGAARNIENGGSLTIIATALIDTGSKMDEVIFEEFKGTGNMELQLDRNLSNKRIFPAVNITASSTRRDDLLLDKTTLDRMWILR<br>KYLADMNPIEAMDFVKDRLEKTRDNDEFLMSMNS
