# Supplementary material for: Short autoinhibitory sequences control phase separation of an essential bacterial transcription termination factor
Source: EMBO J. 2026 May 11;45(12):4124–52. doi: 10.1038/s44318-026-00793-1 (PMC13269538; doi:10.1038/s44318-026-00793-1)
Supplement: Supplementary file 3 — Source data Fig. 1 [file 44318_2026_793_MOESM3_ESM.zip › Figure 1/1C/Rho_Bacteroidetes.rtf]

>BthetaMYNIIQLNDKNLSELQVIAKELGIKKADSFKKEELVYKILDEQAIAGATKKVAAEKLKEERKGDKNKRSRTAAPKKEEKVAPAAKNAEVTKNKENAPAAKPQQQPKEEAANKAKEAPVAEPKAEKAAPKRKVGRPRKDANIAEKAENKEVENAKPIVKPTEEKAVAEKTVVAPAAEKATPTQETEKKVKENKPAVAEKPVIAKPQKKSAPVIDEESTILSSEDDDDFIPIEDLPSEKIELPTELFGKFEATKAETAQAAPEQAPQPQQQQHSQPQQRQRIVRPRDNNNNAGNNNVNANNNNNFQRNNNNNQRPPMQQRPAQQQNNVAENLPAVQQQPERKVIEREKPYEFDDILSGVGVLEIMQDGYGFLRSSDYNYLSSPDDIYVSQSQIKLFGLKTGDVVEGIIRPPKEGEKYFPLVKVSKINGRDAAFVRDRVPFEHLTPLFPDEKFRLCKGGYSDSMSARVVDLFAPIGKGQRALIVAQPKTGKTILMKDIANAIAANHPEVYMIMLLIDERPEEVTDMARSVNAEVIASTFDEPAERHVKIAGIVLEKAKRLVECGHDVVIFLDSITRLARAYNTVSPASGKVLSGGVDANALHKPKRFFGAARNIENGGSLTIIATALIDTGSKMDEVIFEEFKGTGNMELQLDRNLSNKRIFPAVNITASSTRRDDLLLDKTTLDRMWILRKYLADMNPIEAMDFVKDRLEKTRDNDEFLMSMNS>BfragilisMYNIIQLNDKNLSELQAIAQELGIKKTDSLKKEELVYKILDEQAIAGATKKVAADKLKEERKEDKKKRSRVTVKKENADKVFSSTKNGEVTKTDAKTPAAKTQPQPKTTEPTPETAKEANAETNATPAESVKVTPYATPKKKPGRPRKNQVETEAKPAEETTEKPETVPSAQEEKPAAQPETEKRPISKPILKPKPAVVDEESSILSDIDADDDFIPIEDLPSEKVELPTELFGKFESTKAEAATAPEPVAQPQRPRVIRPRDNNNNNNYNNNNNNQRNNNQRQPVQQRPMPQQNAAEAAPVQERRVIEREKPYEFDDILTGTGVLEIMQDGYGFLRSSDYNYLSSPDDIYVSQSQIKLFGLKTGDVVEGVIRPPKEGEKYFPLVKVSKINGRDAAFVRDRVPFDHLTPLFPDEKFKLCKGGYSDSMSARVVDLFSPIGKGQRALIVAQPKTGKTILMKEIANAIAANHPEVYMIMLLIDERPEEVTDMARSVNAEVIASTFDEPAERHVKIAGIVLEKAKRLVECGHDVVIFLDSITRLARAYNTVSPASGKVLSGGVDANALHKPKRFFGAARNIENGGSLTIIATALIDTGSKMDEVIFEEFKGTGNMELQLDRNLSNKRIFPAVNIVASSTRRDDLLLDKQTLDRMWILRKYLSDMNPIEAMDFVKDRLEKTKDNDEFLMSMNS>BovatusMYNIIQLNDKNLSELQVIAKELGIKKADSYKKEDLVYKILDEQAIVGATKKVAADKLKEERKNEEQKKKRSRVAPTKKEDKVVSATKSEEANKTKETAPVKAAPQPSKKEESTNKEKETVVVEAKAENTATPKRKVGRPRKSSDAEEKKEVENAKPAAPKVVEPKPVVAEKATGATEKPAPVQQQTAEKKAKNKPAAEANKPVAETNKPATEPNKPVVEKKVIDKPQKKAAPVIDEESNILSSVDDDDFIPIEDLPSEKIELPTELFGKFEATKTESAQTAPEQPSHPQQQQQSQQQQSQQQRPRIVRSRDNNNGNNNANNNGNNGNNTNNNFQRNNNNQNQVQNPNQNQNQNQNQQRSPMPRAAQQNNAGENLPVQQQQERKVIEREKPYEFDDILNGVGVLEIMQDGYGFLRSSDYNYLSSPDDIYVSQSQIKLFGLKTGDVVEGVIRPPKEGEKYFPLVKVSKINGRDAAFVRDRVPFEHLTPLFPDEKFKLCKGGYSDSMSARVVDLFAPIGKGQRALIVAQPKTGKTILMKDIANAIAANHPEVYMIMLLIDERPEEVTDMARSVNAEVIASTFDEPAERHVKIAGIVLEKAKRLVECGHDVVIFLDSITRLARAYNTVSPASGKVLSGGVDANALHKPKRFFGAARNIENGGSLTIIATALIDTGSKMDEVIFEEFKGTGNMELQLDRNLSNKRIFPAVNITASSTRRDDLLLDKTTLDRMWILRKYLADMNPIEAMDFVKDRLEKTRDNDEFLMSMNS>BstercorisMYNIIQLNDKNLSELQAIAQELGIKKTDSLKKEELVYKILDEQAIAGATKKVAADKLKEERKGDKQKRSRVTVKKEAADKVFTANKNGELTKGKTEEAPAVQASVQAAEAAKEPAVAATPAAETEVPKRKPGRPRKVKQEAAPVAETPKTVPAETKVETTEPKKAEPKKVVAAPQPTPDESPVLPEAEDDFIPIEDLPTEKIELPSELIGKFEATKAETPVPATEQPQQRSRLRLRDNNTPYNNNRNNNNQRPAQQQQRPAQQQNNNENYVASERKPAIEREKAYEFDDILTGTGVLEIMQDGYGFLRSSDYNYLSSPDDIYVSQSQIKLFGLKTGDVVEGVIRPPKEGEKYFPLVKVSKINGRDPAFVRDRVPFEHLTPLFPDEKFKLCKGGYSDSLSARVVDLFAPIGKGQRALIVAQPKTGKTILMKDIANAIAANHPEVYMIMLLIDERPEEVTDMARTVNAEVIASTFDEPAERHVKIAGIVLEKAKRMVECGHDVVIFLDSITRLARAYNTVSPASGKVLSGGVDANALHKPKRFFGAARNIEGGGSLTILATALIDTGSKMDEVIFEEFKGTGNMELQLDRNLSNKRIFPAVNIVASSTRRDDLLQDKQTLDRMWILRKYLADMNPIEAMDFVKDRLEKTKDNDEFLMSMNG>BvulgatusMYNIIQLNDKDLSELQSIAKELGIKKTDSLKKEDLVYKILDEQAIVGATQKVAAEKANESQPRKRSRINVKKDGDKVYTATKDKAQKLEANTPQPAAAAAETVKETAPVAVTPQTEAVAETSAEEKTPKKRGRKPGSKKVTAKTEEQPATKTKETAKEEVKAEAPAKPTPKKAKKEETPAIPEEKEVILPELEFGSETDDFIPIEDLPSEKIELPTELFGKFEATKVETPAPVQTPAPKFQPRQQRDQNQKYNNPRNNNYNNPRQANNNNNTTAEGEASASIPQQPERKQVEKPYEFEGILTGTGVLEIMPDGYGFLRSSDYNYLSSPDDIYVSQSQIKLFGLKTGDVVEGSIRPPKEGEKYFPLVKVEKINGLDPGLVRDRVPFDHLTPLFPDEKFKLCKGGYSDNLSARVVDMFSPIGKGQRALIVAQPKTGKTILMKDIANAIAANHPETYMIMLLIDERPEEVTDMARSVNAEVIASTFDEPAERHVKIAGIVLEKAKRMVECGHDVVIFLDSITRLARAYNTVSPASGKVLSGGVDANALHKPKRFFGAARNIENGGSLTIIATALIDTGSKMDEVIFEEFKGTGNMELQLDRNLSNKRIFPAVNIVASSTRRDDLLLDKTTLDRMWILRKYLADMNPIEAMDFVKSRLENTKDNEEFLMSMNS>BintestinalisMYNIIQLNDKNLSELQSIAQELGIKKTDSLKKEELVYKILDEQAIAGATKKVAAEKLKEERKVDRQQKRTRVTVKKESTDKVFTANKNGDLTKAKAEASVAAAKVQPVAEAVKAPVTEAPKAPVTEKEVAVTATEAPEKAETPNSTKRKPGRPRKAKVEEKAPIPAPVPVAKKEEPKKAEPELNFETEAPKVIKKVVVEDSPILSEPEDDFIPIEDLPTEKVELPSELLGKFEATKAETPAVPVVESVPTNQRPRLRPRDNNNNTPYNNNNNNNRNNNPRPIQQQRPAQPNANNENYSPVPERRPVVEREKVYEFDDILTGTGVLEIMQDGYGFLRSSDYNYLSSPDDIYVSQSQIKLFGLKTGDVVEGVIRPPKEGEKYFPLVKVGKINGRDPAFVRDRVPFEHLTPLFPDEKFRLCKGGYSDSMSARVVDLFAPIGKGQRALIVAQPKTGKTILMKDIANAIAANHPEVYMIMLLIDERPEEVTDMARTVNAEVIASTFDEPAERHVKIAGIVLEKAKRLVECGHDVVIFLDSITRLARAYNTVSPASGKVLSGGVDANALHKPKRFFGAARNIENGGSLTILATALIDTGSKMDEVIFEEFKGTGNMELQLDRNLSNKRIFPAVNIVASSTRRDDLLQDKQTLDRMWILRKYLADMNPIEAMDFVKDRMEKTKDNDEFLMSMNS>BdoreiMYNIIQLNDKDLSELQSIAKELGIKKTDSLKKEDLVYKILDEQAIVGATKKVAAEKANESQPRKRSRINVKKDGDKVYTATKDKAQKLEANTPQPAATAAETVKETAPVAVTPQTETVAETSTEEKTPKKRGRKPGSKKVTAKTEEQPATETKETAKEEVKAEAPAKPTPKKAKKEETPAIPEEKEVILPELEFGSETDDFIPIEDLPSEKIELPTELFGKFEATKVETPAPVQTPAPKFQPRQQRDQNQKYNNPRNNNYNNPRQANNNNNTTAEGEASASIPQQPERKQVEKPYEFEGILTGTGVLEIMPDGYGFLRSSDYNYLSSPDDIYVSQSQIKLFGLKTGDVVEGSIRPPKEGEKYFPLVKVEKINGLDPGLVRDRVPFDHLTPLFPDEKFKLCKGGYSDNLSARVVDMFSPIGKGQRALIVAQPKTGKTILMKDIANAIAANHPETYMIMLLIDERPEEVTDMARSVNAEVIASTFDEPAERHVKIAGIVLEKAKRMVECGHDVVIFLDSITRLARAYNTVSPASGKVLSGGVDANALHKPKRFFGAARNIENGGSLTIIATALIDTGSKMDEVIFEEFKGTGNMELQLDRNLSNKRIFPAVNIVASSTRRDDLLLDKTTLDRMWILRKYLADMNPIEAMDFVKSRLENTKDNEEFLMSMNS>BnordiiMYNIIQLNDKDLSELQTIARELGIKKTDSLKKEELVYKILDEQAIAGATKKVAADKLKEERNEDKKKRSRVAMKKENKVFSATKNGEITKTETAVPVPAAKAQPKEAEPVKAEVVPATEKAVAPPKKKVGRPRKNAVENKPKEEQVAPVAAAETEKTVEVKKAEVKKVEVKKIETKPISKPIIKPKAPAVIDEESNILSESADDDFIPIEDLPSEKVELPTELFGKFESTKTEPVVTPVPEQTQNQQRPRVIRPRDNNNNNNNNPNSNNNAPRNNNPRLPMQRPVQQQNTGENAPAPERKVIEREKPYEFDDILTGTGVLEIMQDGYGFLRSSDYNYLSSPDDIYVSQSQIKLFGLKTGDVVEGIIRPPKEGEKYFPLVKVSKIDGRDAAFVRDRVPFDHLTPLFPDEKFRLCKGGYSDSMSARVVDLFSPIGKGQRALIVAQPKTGKTLLMKDIANAIAANHPEVYMIMLLIDERPEEVTDMARSVNAEVIASTFDEPAERHVKIAGIVLEKAKRLVECGHDVVIFLDSITRLARAYNTVSPASGKVLSGGVDANALHKPKRFFGAARNIENGGSLTIIATALIDTGSKMDEVIFEEFKGTGNMELQLDRNLSNKRIFPAVNIVASSTRRDDLLLDKTTLDRMWILRKYLADMNPIEAMDFVKDRLEKTKDNDEFLMSMNS>BhelcogenesMYNIIQLNDKNLSELQTIAQELGIKKTDSLKKEELVYKILDEQAIAGATKKVAAEKLKDERKGDRQKRSRVTLKTESSDKVFTANKNGNLTKPKTEAVPTTKEQPKAAETVKPATTPAATTSPVETSTTPKRKPGRPRKVKEDTIAPQTTETVKDSQTAEAKPSFEPKAPAAETKVEKKNVPDENPILAEAEDDFIPIEDLPTEKIELPSELIGKFEATKAEISVLAEQPQQPQPQRPRLRPRDNNNPPYSNNNNRNGSQQRPVQQQRSVQQPYNNSDYAVPERKPVEREKAYEFDDILTGTGVLEIMQDGYGFLRSSDYNYLSSPDDIYVSQSQIKLFGLKTGDVVEGVIRPPKEGEKYFPLVKVSKINGRDPAFVRDRVPFEHLTPLFPDEKFRLCKGGYSDSMSARVVDLFAPIGKGQRALIVAQPKTGKTILMKDIANAIAANHPEVYMIMLLIDERPEEVTDMARTVNAEVIASTFDEPAERHVKIAGIVLEKAKRMVECGHDVVIFLDSITRLARAYNTVSPASGKVLSGGVDANALHKPKRFFGAARNIEGGGSLTILATALIDTGSKMDEVIFEEFKGTGNMELQLDRNLSNKRIFPAVNIVASSTRRDDLLQDKQTLDRMWILRKYLSDMNPIEAMDFVKDRLEKTKDNDEFLLSMNS>BsalanitronisMYNIIQLNDKDLSELQQIAKELGLKKTSTLQKEELVYRILDEQAIVGATKKATTGKTNEERKEGQPRKRSRISVKKEGDKVYTATKDKAQKLEASTPPLPAPAPLFKTENVIAATGTSENQPETVPAPENEEKPKPKRGRKPGSKNKSSVEKEQPQVSNDIENTIPEQTEASPAMPDNAELPELNMDEGDDFIAIEDLPSEKIELPTELLGKFEATKMEQPIVPDKDNKSNNNRYQSRQQRHNNRQANGRPNQPAQASADTPQATAAAPEAATAKPAEKLYEFDDILKGSGVLEIMPDGYGFLRSSDYNYLSSPDDIYVSQSQIKLFGLKTGDVVDGTIRPPKEGEKYFPLVKVSKINGLPPELVRDRVPFDHLTPLFPDEKFKLCKGYDDNLSVRIVDLFAPIGKGQRALIVAQPKTGKTILMKDIANAIAANHPEVYMIMLLIDERPEEVTDMSRSVNAEVIASTFDEPAERHVKIAGIVLEKAKRMVECGHDVVIFLDSITRLARAYNTVSPASGKVLSGGVDANALHKPKRFFGAARNIEGGGSLTIIATALIDTGSKMDEVIFEEFKGTGNMELQLDRNLSNKRIFPAVNLTASSTRRDDLLLDKTTLDRMWILRKFLADMNPIEAMNSVKEHLEKTKDNDEFLMSMNS>BcoprocolaMYNIIQLNDKDLSELQQIAKELGLKKTSTLRKEELVYRILDEQAIVGATKKAAAAKTNEERKEGQPRKRSRISVKKEGDKVYTATKDKAQKLEANTPPLPAPAPIFKDEISQPAEVQTETTNADTVAEKPKAKRGRKPGSKNKTTLEKEQLSAPKETEKQTPAVPAPVPVPMENDDMELPALNLTGGDDFIPIEDLPSEKIELPTELLGKFEATKMDTAAPVPAEKPEKENKPNNNKYQARQQRYNNNNNRNNNNNQAGRQQQQHQNTNHPAPQNTTANLPAPQSVVAEQVVQQPKPVEKPYEFDDILSGTGVLEIMPDGYGFLRSSDYNYLSSPDDVYVSQSQIKLFGLKTGDVVEGTIRPPKEGEKYFPLVKVSKINGLDPGLVRDRVPFDHLTPLFPDEKFKLCKGYDDNLSARVVDLFAPIGKGQRALIVAQPKTGKTILMKDIANAIAANHPEVYMIMLLIDERPEEVTDMARSVNAEVIASTFDEPAERHVKIAGIVLEKAKRMVECGHDVVIFLDSITRLARAYNTVSPASGKVLSGGVDANALHKPKRFFGAARNIEGGGSLTIIATALIDTGSKMDEVIFEEFKGTGNMELQLDRNLSNKRIFPAVNITASSTRRDDLLLDKTTLDRMWILRKYLADMNPIEAMDFVKERLERAKDNEEFLMSMNS>BuniformisMYNIIQLNDKNLSELQTIAQELGIKKTDSLKKEELVYKILDEQAIAGATKKVAADKLKEERKGEKQKRSRVTVKTVKKEGADKVFSANKNGDLTKAKTEEAPAAKEQPKTVEAPQEPTIEAATPAPAKEAAVPKRKPGRPRKVKEETAAPQATETPKVAEPELNFEAKAPKTEPQAEAKAEKNIVPDESPILAEAADDFIPIEDLPTEKIELPSELIGKFEATKAETPATPTPAAAAAPAEPQQPQRPRLRARDNNNPYNNNNNNNRNGGYNQQRPVQQRPAQPYNNGENYPAAPERKPVIEREKTYEFDDILTGTGVLEIMQDGYGFLRSSDYNYLSSPDDIYVSQSQIKLFGLKTGDVVEGVIRPPKEGEKYFPLVKVSKINGRDPAFVRDRVPFEHLTPLFPDEKFKLCKGGYSDSMSARVVDLFAPIGKGQRALIVAQPKTGKTILMKDIANAIAANHPEVYMIMLLIDERPEEVTDMARTVNAEVIASTFDEPAERHVKIAGIVLEKAKRMVECGHDVVIFLDSITRLARAYNTVSPASGKVLSGGVDANALHKPKRFFGAARNIEGGGSLTILATALIDTGSKMDEVIFEEFKGTGNMELQLDRNLSNKRIFPAVNIVASSTRRDDLLQDKQTLDRMWILRKYLADMNPIEAMDFVKDRLEKTKDNDEFLMSMNS>BplebeiusMYNIIQLNDKNLSELQQIAKELGLKKVDSLKKEELVYRILDEQAIVGATKKVAAQQNNEERKEGQPRKRSRISIKKEGDKVYTATKDKAQKLEANTPALPAPTPLFKEIPASPEKTEIVAPVAEKTAQPEVIAAPATEEKPKAKRGRKPGSKNKSTLAKEAQEAAESTPAEVTAKPAPLPIDDDDMELPPLDLNNNDFIPIEDLPSEKFEIPSELLGKFEATKMEVPVMPAKEPKQKNQQRQARQNNRNNRPNNNNNNNNQGKDNNPQPAANQNVPEQQPSSPSTETPANNTPAPAPKPVEKVYDFDDILQGTGVLEIMPDGYGFLRSSDYNYLSSPDDIYVSQSQIKLLGLKTGDVVEGTIRPPKEGEKYFPLVKVSKINGLDPALVRDRIPFDHLTPLFPDEKFKLCKGYNDNLSARVVDLFAPIGKGQRALIVAQPKTGKTILMKDIANAIAANHPEVYMIMLLIDERPEEVTDMARSVKAEVIASTFDEPAERHVKIAGIVLEKAKRMVECGHDVVIFLDSITRLARAYNTVSPASGKVLSGGVDANALHKPKRFFGAARNIEGGGSLTIVATALIDTGSKMDEVIFEEFKGTGNMELQLDRNLSNKRIFPAVNIVASSTRRDDLLLDKTTLDRMWILRKYLSDMNPIEAMDFVKDRLEKTKDNEEFLMSMNS>BoleiciplenusMYNIIQLNDKNLSELQTIAQELGIKKTDSLKKEELVYKILDEQAIAGATKKVAAEKLKEERKVDRQEKRTRVTVKKESTDKVFTANKNGDLTKAKAEATAASAKVRPITEAAKTPAAEKAPEVTVTATPEKAEDSTTPKRKPGRPRKAKSEEKTPAPIVKEEPQKTEPELSFEAETPKTSTSKIPAPKVVAPQVVKKVVVEDSPILSEPEDDFIPIEDLPTEKVELPSELLGKFEATKAETPAVAPITEPAPAMAQRPRLRPRDNNNNTPYNNNNRNNNQRPVQQQRPVQPQNGGENYAPVPERRTVIEREKVYEFDDILTGTGVLEIMQDGYGFLRSSDYNYLSSPDDIYVSQSQIKLFGLKTGDVVDGVIRPPKEGEKYFPLVKVEKINGRDPAFVRDRVPFEHLTPLFPDEKFRLCKGGYSDSMSARVVDLFAPIGKGQRALIVAQPKTGKTILMKDIANAIAANHPEVYMIMLLIDERPEEVTDMARTVNAEVIASTFDEPAERHVKIAGIVLEKAKRLVECGHDVVIFLDSITRLARAYNTVSPASGKVLSGGVDANALHKPKRFFGAARNIENGGSLTILATALIDTGSKMDEVIFEEFKGTGNMELQLDRNLSNKRIYPAVNIVASSTRRDDLLQDKQTLDRMWILRKYLADMNPIEAMDFVKDRMEKTRDNDEFLLSMNS>BcellulosilyticusMYNIIQLNDKNLSELQSIAQELGIKKTDSLKKEELVYKILDEQAIAGATKKVAAEKLKEERKVDRQQKRTRVTVKKESTDKVFTANKNGDLTKAKAETTVAAAKVQPVAEATKAPVAEKAPEAVVTTASAPEKAEDATPKRKPGRPRKTKTEEKAPIPAPMPVAKKEEPKKAEPELNFETEAPKVVKKVVIEDSPILSEPEDDFIPIEDLPTEKIELPSELLGKFEATKAETPIAPVAEPTPATAQRPRLRPRDNNNTPYNNNNNNNRNNNPRPVQQQRPAQQNAGDNYAPVQERRPVIEREKVYEFDDILTGTGVLEIMQDGYGFLRSSDYNYLSSPDDIYVSQSQIKLFGLKTGDVVEGVIRPPKEGEKYFPLVKVGKINGRDPAFVRDRVPFEHLTPLFPDEKFRLCKGGYSDSMSARVVDLFAPIGKGQRALIVAQPKTGKTILMKDIANAIAANHPEVYMIMLLIDERPEEVTDMARTVNAEVIASTFDEPAERHVKIAGIVLEKAKRLVECGHDVVIFLDSITRLARAYNTVSPASGKVLSGGVDANALHKPKRFFGAARNIENGGSLTILATALIDTGSKMDEVIFEEFKGTGNMELQLDRNLSNKRIFPAVNIVASSTRRDDLLQDKQTLDRMWILRKYLADMNPIEAMDFVKDRMEKTKDNDEFLMSMNS>BcaccaeMLSIIQQKRNINNLPYYMYNIIQLNDKDLSELQVIAKELGIKKTDSLKKEDLVYKILDEQAIAGATKKVAADKLKEERKEEQKKKRSRVAPAKKDNKVVSATKEGEAEKAKEAAPAKPQQPSKKEESANKEKETPAVEVKAENTAAPKRKVGRPRKNADAAEQKEVESVKTATPATPKVTEDKVVTEKAPEVIEKAVPAQAPEKKTKANKPAEEKKVVVKPQPQKKAEPVIDEESNILSGADDDDFIPIEDLPSEKIELPTELFGKFEATKTEPAQTATEQQAPQPQQQAHQQQQQQRPRIVRPRDNNNGNNNASNSNNNANNNNNNNFQRNNNQNQNQQRVPMPRPAQPNNANENLPVPQQQQERKVIEREKPYEFDDILNGVGVLEIMQDGYGFLRSSDYNYLSSPDDIYVSQSQIKLFGLKTGDVVEGVIRPPKEGEKYFPLVKVSKINGRDAAFVRDRVPFEHLTPLFPDEKFKLCKGGYSDSMSARVVDLFAPIGKGQRALIVAQPKTGKTILMKDIANAIAANHPEVYMIMLLIDERPEEVTDMARSVNAEVIASTFDEPAERHVKIAGIVLEKAKRLVECGHDVVIFLDSITRLARAYNTVSPASGKVLSGGVDANALHKPKRFFGAARNIENGGSLTIIATALIDTGSKMDEVIFEEFKGTGNMELQLDRNLSNKRIFPAVNITASSTRRDDLLLDKTTLDRMWILRKYLADMNPIEAMDFVKDRLEKTRDNDEFLMSMNS>BsalyersiaeMYNIIQLNDKDLSELQTIAHELGIKKTDSLKKEELVYKILDEQAIAGATKKVAADKLKEERNEDKKKRSRVAVKKENKVFSATKNGEITKAETTAPAPATKAQAQEAATVKQETATPAAETPAAAPKKKVGRPRKNPVVNKPVEEQPAAKEEKEVVVTETKKAEEEKKTEAKPASKPISKPASKPISKPIIKPKAPAVVDEESKILSESSDDDFIPIEDLPSEKVELPTELFGKFESTKAETITLPAPEQAQNQPRPRVIRPRDNNNNSNNNANNNNNAPRNNNNPRLPMQRPAQQQNAGENVPAPERKVIEREKPYEFDDILTGTGVLEIMQDGYGFLRSSDYNYLSSPDDIYVSQSQIKLFGLKTGDVVEGVIRPPKEGEKYFPLVKVSKIDGRDAAFVRDRVPFDHLTPLFPDEKFRLCKGGYSDSMSARVVDLFSPIGKGQRALIVAQPKTGKTLLMKDIANAIAANHPEVYMIMLLIDERPEEVTDMARSVNAEVIASTFDEPAERHVKIAGIVLEKAKRLVECGHDVVIFLDSITRLARAYNTVSPASGKVLSGGVDANALHKPKRFFGAARNIENGGSLTIIATALIDTGSKMDEVIFEEFKGTGNMELQLDRNLSNKRIFPAVNIVASSTRRDDLLLDKTTLDRMWILRKYLADMNPIEAMDFVKDRLEKTKDNDEFLMSMNS>BxylanisolvensMYNIIQLNDKNLSELQVIAKELGIKKADSYKKEDLVYKILDEQAIVGATKKVAADKLKEERKNEEQKKKRSRVAPTKKEDKVVSTPKSGEVNKTKEATPVKAPQPSKKEESTNKEKEAPVVEAKAENATTAPKRKVGRPRKSADAEEKKEVENVTPAAPKVVETKPVVAEKTTETKEKAAPAQQPTAEKKAKSKPAAETNKPAAEPNKPVAEKKVIDKPQKKAAPVIDEESNILSSVDDDDFIPIEDLPSEKIELPTELFGKFEATKTEPVQTAPEQPSHPQQQQQSQQQQAQQQRPRIVRPRDNNNGNNNVNNNSNNANNNNNNFQRNNNNQNQVQNPNQNQNQQRLPMPRATQQNHANENLPAQQQQQQERKVIEREKPYEFDDILNGVGVLEIMQDGYGFLRSSDYNYLSSPDDIYVSQSQIKLFGLKTGDVVEGVIRPPKEGEKYFPLVKVSKINGRDAAFVRDRVPFEHLTPLFPDEKFKLCKGGYSDSMSARVVDLFAPIGKGQRALIVAQPKTGKTILMKDIANAIAANHPEVYMIMLLIDERPEEVTDMARSVNAEVIASTFDEPAERHVKIAGIVLEKAKRLVECGHDVVIFLDSITRLARAYNTVSPASGKVLSGGVDANALHKPKRFFGAARNIENGGSLTIIATALIDTGSKMDEVIFEEFKGTGNMELQLDRNLSNKRIFPAVNITASSTRRDDLLLDKTTLDRMWILRKYLADMNPIEAMDFVKDRLEKTRDNDEFLMSMNS>BfluxusMYNIIQLNDKSLSELQTIAQELGIKKTDSLKKEELVYKILDEQAIAGATKKVAADKLKEERKGEKQKRSRVTVKKESADKVFTANKNGDLTKSKTEEAPSTPKQPKAAEPAKEPVTAAAAATSASSPESAPATPAATPKRKPGRPRKVKEEEVAAPTAEAAKKPEITEPELNFEPKAPAKETKAEKKAVVEENPILTEAEDDFIPIEDLPTEKIELPSELIGKFEATKAETPAPAELPQQAQPQPQRPRPRPRDNNAPYNNNNNRNNNYPQRPIQQQRPAQQPYNSGDNYPAERKPVEREKAYEFDDILTGTGVLEIMQDGYGFLRSSDYNYLSSPDDIYVSQSQIKLFGLKTGDVVEGVIRPPKEGEKYFPLVKVSKINGRDPAFVRDRVPFEHLTPLFPDEKFKLCKGGYSDSLSARVVDLFAPIGKGQRALIVAQPKTGKTILMKDIANAIAANHPEVYMIMLLIDERPEEVTDMARTVNAEVIASTFDEPAERHVKIAGIVLEKAKRMVECGHDVVIFLDSITRLARAYNTVSPASGKVLSGGVDANALHKPKRFFGAARNIEGGGSLTILATALIDTGSKMDEVIFEEFKGTGNMELQLDRNLSNKRIFPAVNIVASSTRRDDLLQDKQTLDRMWILRKYLSDMNPIEAMDFVKDRLEKTKDNDEFLLSMNG>BfinegoldiiMYNIIQLNDKDLSELQIIAKELGIKKTDSYKKEDLVYKILDEQAIVGATKKVAADKLKEERKEEKKKRSRVTPAKKEDKVVSATKTGEVTKTKEAAPAKAQQAPKEETTNKEKETPAVEAKAENAVAPKRKVGRPRKNSDTTDKKEVEETKQVASNSVEAKPVVAEKAPETTEKTAPAQQQSTEKKEKPSKPAVEKDKPAAEKAVEKKVVAKPQKKAEPVIDEESNILTGADDDDFIPIEDLPSEKIELPTELFGKFEATKTEPVQSAPEQVAQPQQQQSQSQQQRPRIVRPRDNNNANNNANNNFQRNNNQNQGQNQNQHQQRLPMPRATQQNNASENLPAQQPQERKAIEREKPYEFDDILNGVGVLEIMQDGYGFLRSSDYNYLSSPDDIYVSQSQIKLFGLKTGDVVEGVIRPPKEGEKYFPLVKVSKINGRDAAFVRDRVPFEHLTPLFPDEKFKLCKGGYSDSMSARVVDLFAPIGKGQRALIVAQPKTGKTILMKDIANAIAANHPEVYMIMLLIDERPEEVTDMARSVNAEVIASTFDEPAERHVKIAGIVLEKAKRLVECGHDVVIFLDSITRLARAYNTVSPASGKVLSGGVDANALHKPKRFFGAARNIENGGSLTIIATALIDTGSKMDEVIFEEFKGTGNMELQLDRNLSNKRIFPAVNITASSTRRDDLLLDKTTLDRMWILRKYLADMNPIEAMDFVKDRLEKTRDNDEFLMSMNS>BpyogenesMYNIIQLNDKNLSELQVIAKELGIKKTESLKKEELVYKILDEQAIAGATKKVAADKLKEERKEEKKKRSRVAAPQKEDKVMSATKDGEITKPKATPDPQPQVLPKEKTAGRDDESSATAEKTGKTTESKRKVGRPRKETAKAKKEAETVAKTEKTTEEKTVAEKKAVVADDVQTSKETEKKAETPKTTPETATKPNKPAPVVDEESAILSSADEDDFIPIEDLPSERIELPTELVGKFEATKAEPAQAPTEPGTQPQQRPRIARQRDNNNAGNNNAQRNNPRQQMQRPAQQNNLNESTQPQQQERKAPEPREKAYEFDDILSGTGVLEIMQDGYGFLRSSDYNYLSSPDDIYVSQSQIKLFGLKTGDVVEGVIRPPKEGEKYFPLVKVSRINGRDAAFVRDRVPFEHLTPLFPDEKFKLCKGGYSDSMSARVVDLFSPIGKGQRALIVAQPKTGKTILMKDIANAIAANHPEVYMIMLLIDERPEEVTDMARSVNAEVIASTFDEPADRHVKIAGIVLEKAKRLVECGHDVVIFLDSITRLARAYNTVSPASGKVLSGGVDANALHKPKRFFGAARNIENGGSLTIIATALIDTGSKMDEVIFEEFKGTGNMELQLDRNLSNKRIFPSVNITASSTRRDDLLLDKTTLDRMWILRKYLADMNPLEAMDFVKDRLEKTKDNDEFLMSMNS>BeggerthiiMYNIIQLNDKNLSELQAIAQELGIKKTDSLKKEELVYKILDEQAIAGATKKVAADKLKEERKGDKQKRSRVTVKKEGTDKVFTANKNGELTKGKTGETPATQTSAQTVETAKETAASSTPATETEAPKRKPGRPRKVKQEAAPVTEVSKTAQTETKAEVAEPQKAEIKKAEPKVTPAAPQPTPDETPVLPEVEDDFIPIEDLPTEKIELPSELIGKFEATKAETPVPVAEQPQQQHPRLRLRDNNTPYNNNRNNNNSQRPAQQQRPAQQQNSNDNNYAAPERKPVAEREKAYEFDDILTGTGVLEIMQDGYGFLRSSDYNYLSSPDDIYVSQSQIKLFGLKTGDVVEGVIRPPKEGEKYFPLVKVSKINGRDPAFVRDRVPFEHLTPLFPDEKFRLCKGGYSDSMSARVVDLFAPIGKGQRALIVAQPKTGKTILMKDIANAIAANHPEVYMIMLLIDERPEEVTDMARTVNAEVIASTFDEPAERHVKIAGIVLEKAKRMVECGHDVVIFLDSITRLARAYNTVSPASGKVLSGGVDANALHKPKRFFGAARNIEGGGSLTILATALIDTGSKMDEVIFEEFKGTGNMELQLDRNLSNKRIFPAVNIVASSTRRDDLLQDKQTLDRMWILRKYLADMNPIEAMDFVKDRMEKTKDNDEFLMSMNS>BmassiliensisMNCDLKTFHVPIAFFDYTIKKYFTNNIYYMYNIIQLNDKDLSELQSIAKELGITKTESLKKEELVYKILDEQAIVGATKKVAAAKVNEDRKENQPKKRSRISVKKEGDKVYTATKDKAQKLEAATPTTAAHIAEEAEKEAVSTVETAQPIQEKTVTEAVEKSEPKKRGRKPGTKNKTAAKTEEQPATTAEETNNTTPQKVSATNTPKEKEVILPELDFDSDTDDFIPIEDLPSEKIELPTELLGKFEATKNEAPVPTVTPASKFQPRQPREQNPRYNNPNQRNNNNYNNQRPTNNNNTEGLESPQQPQQSAPERKPVEKPYEFEGILTGTGVLEIMPDGYGFLRSSDYNYLSSPDDIYVSQSQIKLFGLKTGDVVEGSIRPPKEGEKYFPLVKVEKINGLDPGLVRDRVPFDHLTPLFPDEKFRLCKGGYSDNLSARVVDMFAPIGKGQRALIVAQPKTGKTILMKDIANAIAANHPEVYMIMLLIDERPEEVTDMARSVNAEVIASTFDEPAERHVKIAGIVLEKAKRMVECGHDVVIFLDSITRLARAYNTVSPASGKVLSGGVDANALHKPKRFFGAARNIEGGGSLTIIATALIDTGSKMDEVIFEEFKGTGNMELQLDRNLSNKRIFPAVNIVASSTRRDDLLLDKTTLDRMWILRKYLADMNPIEAMDFVKSRLENTRDNEEFLMSMNS>BclarusMYNIIQLNDKNLSELQAIAQELGIKKTDSLKKEELVYKILDEQAIAGATKKVAADKLKEERKGDKQKRSRVTVKKEGADKVFTANKNGDLTKGKAEETPAVQTPVQAAEAAKEPAVATAPAAEAEAPKRKPGRPRKVKQEAAPVAEAPKKVAPAEPKAETAEPKKAEPKKAVAAPQPTPDESPILPEVEDDFIPIEDLPTEKIELPSELIGKFEATKAETPVPVAEQPQQRPRLRLRDNNTPYNNNRNNNNNQRPAQQQQRPVQQQNNTENYVAPERKPAAEREKAYEFDDILTGTGVLEIMQDGYGFLRSSDYNYLSSPDDIYVSQSQIKLFGLKTGDVVEGVIRPPKEGEKYFPLVKVSKINGRDPAFVRDRVPFEHLTPLFPDEKFKLCKGGYSDSMSARVVDLFAPIGKGQRALIVAQPKTGKTILMKDIANAIAANHPEVYMIMLLIDERPEEVTDMARTVNAEVIASTFDEPAERHVKIAGIVLEKAKRMVECGHDVVIFLDSITRLARAYNTVSPASGKVLSGGVDANALHKPKRFFGAARNIEGGGSLTILATALIDTGSKMDEVIFEEFKGTGNMELQLDRNLSNKRIFPAVNIVASSTRRDDLLQDKQTLDRMWILRKYLADMNPIEAMDFVKDRLEKTKDNDEFLMSMNG>BfaecisMYNIIQLNDKNLSELQIIAKELGIKKADSFKKEELVYKILDEQAIAGATKKVAAEKLKEERKGEKSKRSRTAVPKKEEKAAPAPKNAEVAKNKENAPVAKVQQQPKEEAANKAKEAPAAEPKAENAAPKRKVGRPRKDANTTEKAESKEVENAKPIVKATEEKAVAEKTVVAPAEKAAPVQETEKKVKENKPAIAEKPVIAKPQKKSAPVIDEESNILSSDDDDDFIPIEDLPSEKIELPTELFGKFEATKAETAQAAPEQAPQPQQQHSQPQQRQRIVRPRDNNNNNNNAGNNNANANANNNNNFQRNNNNNQRPPMQQRPAPQQNNVAESVPPVQQQPERKVIEREKPYEFDDILSGVGVLEIMQDGYGFLRSSDYNYLSSPDDIYVSQSQIKLFGLKTGDVVEGIIRPPKEGEKYFPLVKVSKINGRDAAFVRDRVPFEHLTPLFPDEKFRLCKGGYSDSMSARVVDLFAPIGKGQRALIVAQPKTGKTILMKDIANAIAANHPEVYMIMLLIDERPEEVTDMARSVNAEVIASTFDEPAERHVKIAGIVLEKAKRLVECGHDVVIFLDSITRLARAYNTVSPASGKVLSGGVDANALHKPKRFFGAARNIENGGSLTIIATALIDTGSKMDEVIFEEFKGTGNMELQLDRNLSNKRIFPAVNITASSTRRDDLLLDKTTLDRMWILRKYLADMNPIEAMDFVKDRLEKTRDNDEFLMSMNS>BsartoriiMYNIIQLNDKDLSELQSIAKELGIKKTDSLKKEDLVYRILDEQAIVGATKKVAAEKANEGQPRKRSRINVKKEGDKVYTATKDKAQKLEANTPQPASATAETVKEAAPAAVTPPTEAVAEASAEEKTPKKRGRKPGSKKVTAKTEEQPATETKETVKEEVKAEAPAKPKPQKAKKEETPATPKEKEVILPELEFSGETEDFIPIEDLPSEKIELPTELFGKFEATKVETPAPVQTPAPKFQPRQQRDQNQKYNNPRNNNYNNPRQANNNNNNTAAEGEAPANIPQQPERKQVEKPYEFEGILTGTGVLEIMPDGYGFLRSSDYNYLSSPDDIYVSQSQIKLFGLKTGDVVEGSIRPPKEGEKYFPLVKVEKINGLDPGLVRDRVPFDHLTPLFPDEKFKLCKGGYSDNLSARVVDMFSPIGKGQRALIVAQPKTGKTILMKDIANAIAANHPETYMIMLLIDERPEEVTDMARSVNAEVIASTFDEPAERHVKIAGIVLEKAKRMVECGHDVVIFLDSITRLARAYNTVSPASGKVLSGGVDANALHKPKRFFGAARNIENGGSLTIIATALIDTGSKMDEVIFEEFKGTGNMELQLDRNLSNKRIFPAVNIVASSTRRDDLLLDKTTLDRMWILRKYLADMNPIEAMDFVKSRLENTKDNEEFLMSMNS>BheparinolyticusMYNIIQLNDKNLSELQTIAQELGIKKTESLKKEELVYKILDEQAIAGATKKVAADKLKEERKGDKRSRVTVKTVKKESVNKVFSANKNGELTKSKNEGTPIIKEQAKVAEAAPTTKPANTAATEASKPANTVVATSPKNTESSSKRKPGRPRKTQEEADAASKATENAKVTEPELNFEPKATKTEPNKVAKNIVVDESPILSEAEDDFIPIEDLPTEKTELPSELIGKFEATKTEVPATPAPMPTEQQRPHLRSRDNNNNNRAGAYNQQRPMQQRPAQQHGGNENNPAAADNKPAVEREKAYEFDDILTGTGVLEIMQDGYGFLRSSDYNYLSSPDDIYVSQSQIKLFGLKTGDVVEGVIRPPKEGEKYFPLVKVSKINGRDPAFVRDRVPFEHLTPLFPDEKFKLCKGGYSDSLSARVVDLFAPIGKGQRALIVAQPKTGKTILMKDIANAIAANHPEVYMIMLLIDERPEEVTDMARTVNAEVIASTFDEPAERHVKIAGIVLEKAKRMVECGHDVVIFLDSITRLARAYNTVSPASGKVLSGGVDANALHKPKRFFGAARNIEGGGSLTILATALIDTGSKMDEVIFEEFKGTGNMELQLDRNLSNKRIFPAVNIVASSTRRDDLLQDKQTLDRMWILRKYLADMNPMEAMDFVKDRLEKTKDNDEFLMSMNS>BcaecimurisMYNIIQLNDKNLSELQAIAKELGIKKADSYKKEDLVYKILDEQAIVGATKKVAADKLKEERKNEEQKKKRSRVAPAKKEDKVVSATKNGEVTKTKEAIPVKAQQPSKKEESTNKDKEAPVVEAKSENATTAPKRKVGRPRKNADAEEKKEVENAKLAAPKAVETGSVVAEKTTETTEKTAPAQQPTAEKKAKSKPVVEINKPAADPNKPVAEKKNIDKLQKKTALVIDEESNILSSVDDDDFIPIEDLPSEKIELPTELFGKFEATKTESVQTAPEQPSHPQQQQQPQQQQAQQQRPRIVRPRDNNNGNNNANNNNANNNFQRNNNNQNQQRLPMPRATQQNHASDNLPAQQQQERKVIEREKPYEFNDILNGVGVLEIMQDGYGFLRSSDYNYLSSPDDIYVSQSQIKLFGLKTGDVVEGVIRPPKEGEKYFPLVKVSKINGRDAAFVRDRVPFEHLTPLFPDEKFKLCKGGYSDSMSARVVDLFAPIGKGQRALIVAQPKTGKTILMKDIANAIAANHPEVYMIMLLIDERPEEVTDMARSVNAEVIASTFDEPAERHVKIAGIVLEKAKRLVECGHDVVIFLDSITRLARAYNTVSPASGKVLSGGVDANALHKPKRFFGAARNIENGGSLTIIATALIDTGSKMDEVIFEEFKGTGNMELQLDRNLSNKRIFPAVNITASSTRRDDLLLDRTTLDRMWILRKYLADMNPIEAMDFVKDRLDKTRDNDEFLMSMNS>BacidifaciensMYNIIQLNDKELSELQIIAKDLGIKKADSFKKEDLVYKILDEQAIVGATKKVAADKLKEERKEDKKKRSPRVTPAKKEDKAVSAAKGGEVAKTKEATPVKTQQPSKEENTNKEKETPAIDAKNENAAPKRKVGRPRKNSETTEKKEVENAKPVVTKAMEAKPVAAEKPTEATQKAAPVSQPAEKKEKPNKPVVETNSSTAETNKSAAEANKPSEKKAIAKPQKKAAPAIDEESNILSNADDDDFIPIEDLPSEKIELPTELFGKFEATKAEPAQMLTEQAPYSQQQQPQQHPSQQQQQAQQQRPRIVRPRDNNANNNNNFQRNNNQNQAQNQQRLPMQRSNQQNNASENFPAQQPQERKIIEREKPYEFDDILNGVGVLEIMQDGYGFLRSSDYNYLSSPDDIYVSQSQIKLFGLKTGDVVEGVIRPPKEGEKYFPLVKVSKINGRDAAFVRDRVPFEHLTPLFPDEKFKLCKGGYSDSMSARVVDLFAPIGKGQRALIVAQPKTGKTILMKDIANAIAANHPEVYMIMLLIDERPEEVTDMARSVNAEVIASTFDEPAERHVKIAGIVLEKAKRLVECGHDVVIFLDSITRLARAYNTVSPASGKVLSGGVDANALHKPKRFFGAARNIENGGSLTIIATALIDTGSKMDEVIFEEFKGTGNMELQLDRNLSNKRIFPAVNITASSTRRDDLLLDKTTLDRMWILRKYLADMNPIEAMDFVKDRLEKTKDNDEFLMSMNS>BfaecichinchillaeMYNIIQLNDKNLSELQVIAQELGIKKADSFKKEELVYKILDEQAIVGATKKVAADKLKEERKEDKKKRSRVTPVKKENKVMSATKEGEVTKNKETAPVAKPQPKEEIVKEETVVKEDINKEVANKEVITKEKEAPVAETKAEQNATPKRKVGRPRKEAKPTVKKETEVVKPVEKVVEKAPEQKVVAEPTVITTVKPTPVETENKVKVNQPISELKPILKPKKAEPVIDEESNILSGSDDEDFIPIEDLPSEKIELPTELFGKFEATKAESTQTEPEQAPQPQQQQVQQPQQNIHRQRVIRTRENNGNYNANNSNNANNNNPNNSNFQRNNQRTQMQRPVPQNNAGENLPVQQERKVIEREKPYEFDDILNGVGVLEIMQDGYGFLRSSDYNYLSSPDDIYVSQSQIKLFGLKTGDVVEGVIRPPKEGEKYFPLVKVSKINGRDAAFVRDRVPFEHLTPLFPDEKFKLCKGGYSDSMSARVVDLFAPIGKGQRALIVAQPKTGKTILMKDIANAIAANHPEVYMIMLLIDERPEEVTDMARSVNAEVIASTFDEPAERHVKIAGIVLEKAKRLVECGHDVVIFLDSITRLARAYNTVSPASGKVLSGGVDANALHKPKRFFGAARNIENGGSLTIIATALIDTGSKMDEVIFEEFKGTGNMELQLDRNLSNKRIFPAVNITASSTRRDDLLLDKTTLDRMWILRKYLADMNPIEAMDFVKDRLEKTRDNEEFLMSMNS
