# Supplementary figures and images for: Short autoinhibitory sequences control phase separation of an essential bacterial transcription termination factor
Source: EMBO J. 2026 May 11;45(12):4124–52. doi: 10.1038/s44318-026-00793-1 (PMC13269538; doi:10.1038/s44318-026-00793-1)

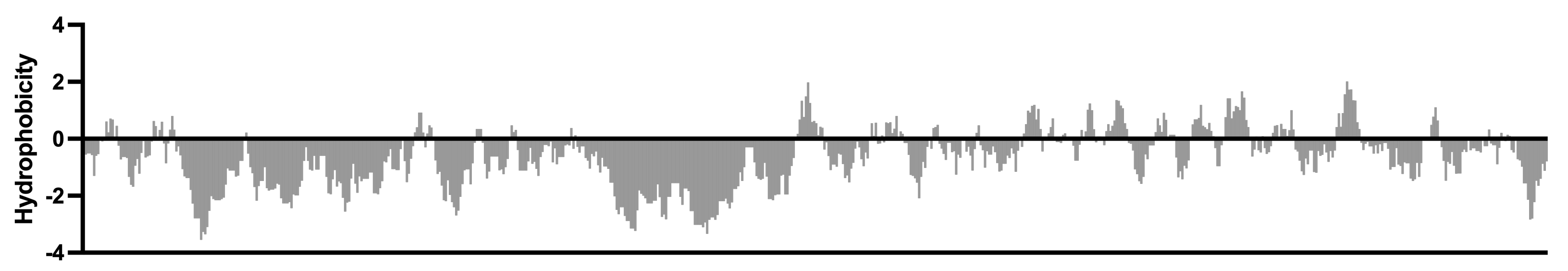

Supplement: Supplementary file 3 — Source data Fig. 1 [file 44318_2026_793_MOESM3_ESM.zip › Figure 1/1A/hydrophobicity.tiff]

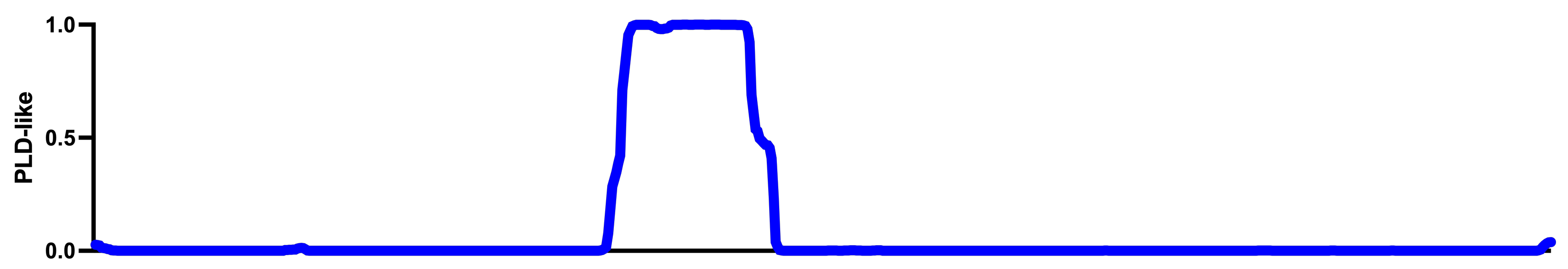

Supplement: Supplementary file 3 — Source data Fig. 1 [file 44318_2026_793_MOESM3_ESM.zip › Figure 1/1A/PLAAC.tiff]

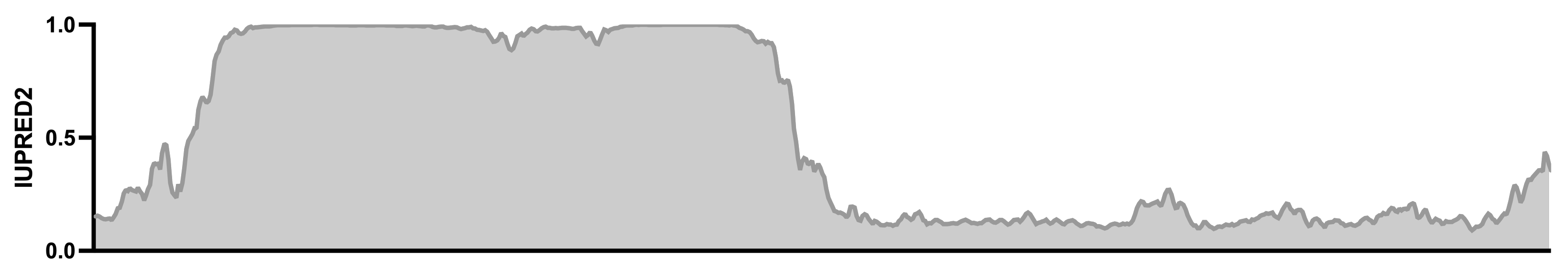

Supplement: Supplementary file 3 — Source data Fig. 1 [file 44318_2026_793_MOESM3_ESM.zip › Figure 1/1A/AIUPred_disorder.tiff]

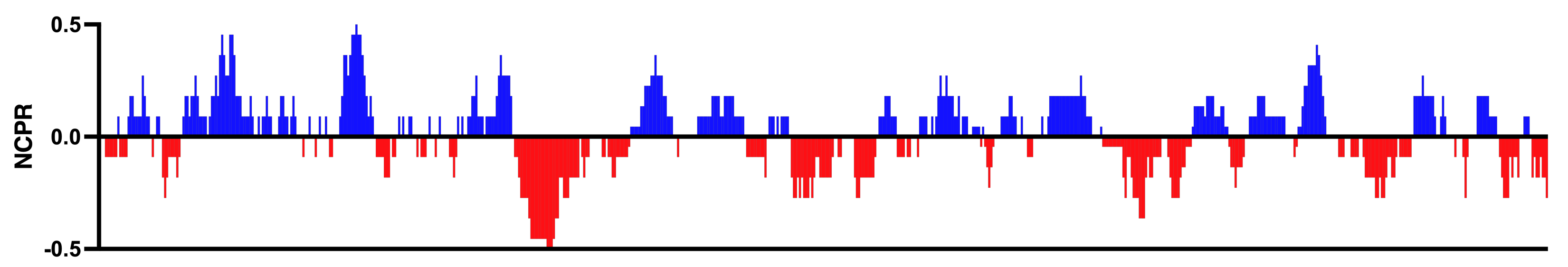

Supplement: Supplementary file 3 — Source data Fig. 1 [file 44318_2026_793_MOESM3_ESM.zip › Figure 1/1A/charge.tiff]

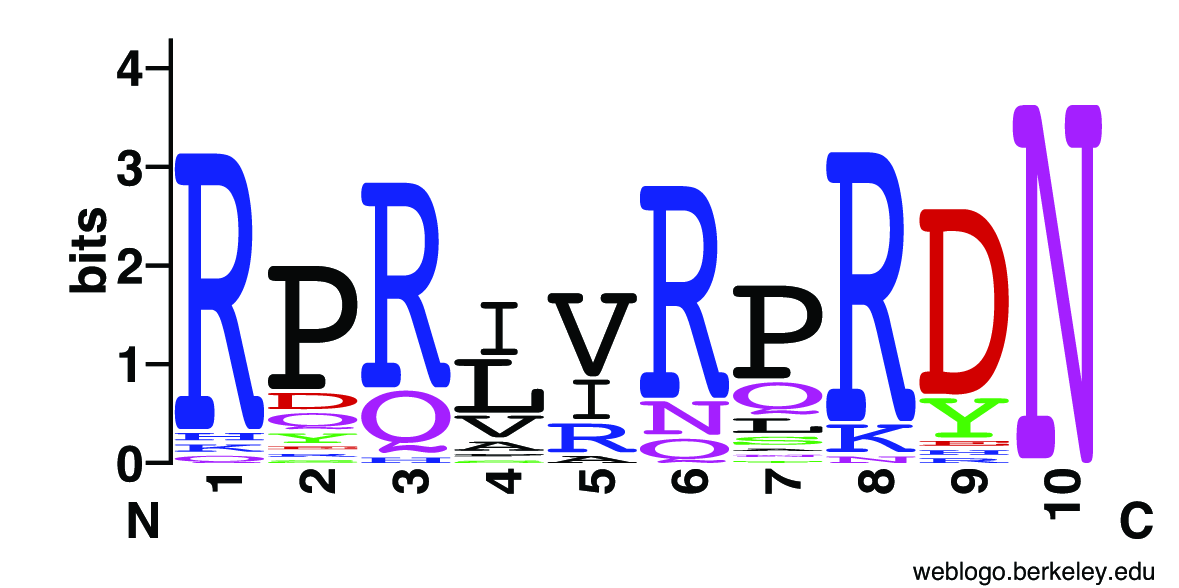

Supplement: Supplementary file 6 — Source data Fig. 4 [file 44318_2026_793_MOESM6_ESM.zip › Figure 4/4A/PLDmotif.tif]
