# Supplementary material for: Short autoinhibitory sequences control phase separation of an essential bacterial transcription termination factor
Source: EMBO J. 2026 May 11;45(12):4124–52. doi: 10.1038/s44318-026-00793-1 (PMC13269538; doi:10.1038/s44318-026-00793-1)
Supplement: Supplementary file 5 — Source data Fig. 3 [file 44318_2026_793_MOESM5_ESM.zip › Figure 3/README.rtf]

All the raw microscopy data for Figures 3D, 3E, 3F and 3G have been submitted to Biostudies-Bioimages S-BIAD2753.
