# Supplementary material for: Short autoinhibitory sequences control phase separation of an essential bacterial transcription termination factor
Source: EMBO J. 2026 May 11;45(12):4124–52. doi: 10.1038/s44318-026-00793-1 (PMC13269538; doi:10.1038/s44318-026-00793-1)
Supplement: Supplementary file 6 — Source data Fig. 4 [file 44318_2026_793_MOESM6_ESM.zip › Figure 4/4A/PLDmotif.rtf]

>Btheta/277-287RQRIVRPRD---NN>Bfragilis/255-268RPRVIRPRDNNNNN>Bovatus/303-316RPRIVRSRDNNNGN>Bstercoris/231-240-SRL-RLRD--NNT>Bvulgatus/246-256RDQ--NQKYNNPR->Bintestinalis/253-265RPRL-RPRDNNNNT>Bdorei/246-256RDQ--NQKYNNPR->Bnordii/248-261RPRVIRPRDNNNNN>Bhelcogenes/237-248RPRL-RPRD-NNNP>Bsalanitronis/234-243RYQSRQQRHN---->Bcoprocola/237-249KYQARQQRYNNNN->Buniformis/247-257RPRL-RARD--NNN>Bplebeius/240-252QRQARQNNRNNRP->Boleiciplenus/257-269RPRL-RPRDNNNNT>Bcellulosilyticus/247-258RPRL-RPRD-NNNT>Bcaccae/298-311RPRIVRPRDNNNGN>Bsalyersiae/256-269RPRVIRPRDNNNNS>Bxylanisolvens/296-309RPRIVRPRDNNNGN>Bfluxus/247-257RPRP-RPRD--NNA>Bfinegoldii/286-299RPRIVRPRDNNNAN>Bpyogenes/259-272RPRIARQRDNNNAG>Beggerthii/236-246HPRL-RLRD--NNT>Bmassiliensis/270-281REQ--NPRYNNPNQ>Bclarus/232-241-PRL-RLRD--NNT>Bfaecis/275-288RQRIVRPRDNNNNN>Bsartorii/246-256RDQ--NQKYNNPR->Bheparinolyticus/249-258RPHL-RSRD--NN->Bcaecimuris/296-309RPRIVRPRDNNNGN>Bacidifaciens/303-312RPRIVRPRDN---->Bfaecichinchillae/295-307RQRVIRTRENN-GN
