# Supplementary material for: Short autoinhibitory sequences control phase separation of an essential bacterial transcription termination factor
Source: EMBO J. 2026 May 11;45(12):4124–52. doi: 10.1038/s44318-026-00793-1 (PMC13269538; doi:10.1038/s44318-026-00793-1)
Supplement: Supplementary file 8 — Source data Fig. 6 [file 44318_2026_793_MOESM8_ESM.zip › Figure 6/README.rtf]

All the raw microscopy data for figures 6A, 6B and 6F have been submitted to Biostudies-Bioimages S-BIAD2753.
