# Supplementary material for: Short autoinhibitory sequences control phase separation of an essential bacterial transcription termination factor
Source: EMBO J. 2026 May 11;45(12):4124–52. doi: 10.1038/s44318-026-00793-1 (PMC13269538; doi:10.1038/s44318-026-00793-1)
Supplement: Supplementary file 8 — Source data Fig. 6 [file 44318_2026_793_MOESM8_ESM.zip › Figure 6/6E/DRaCALA_triplicates.pptx]

## Slide 1
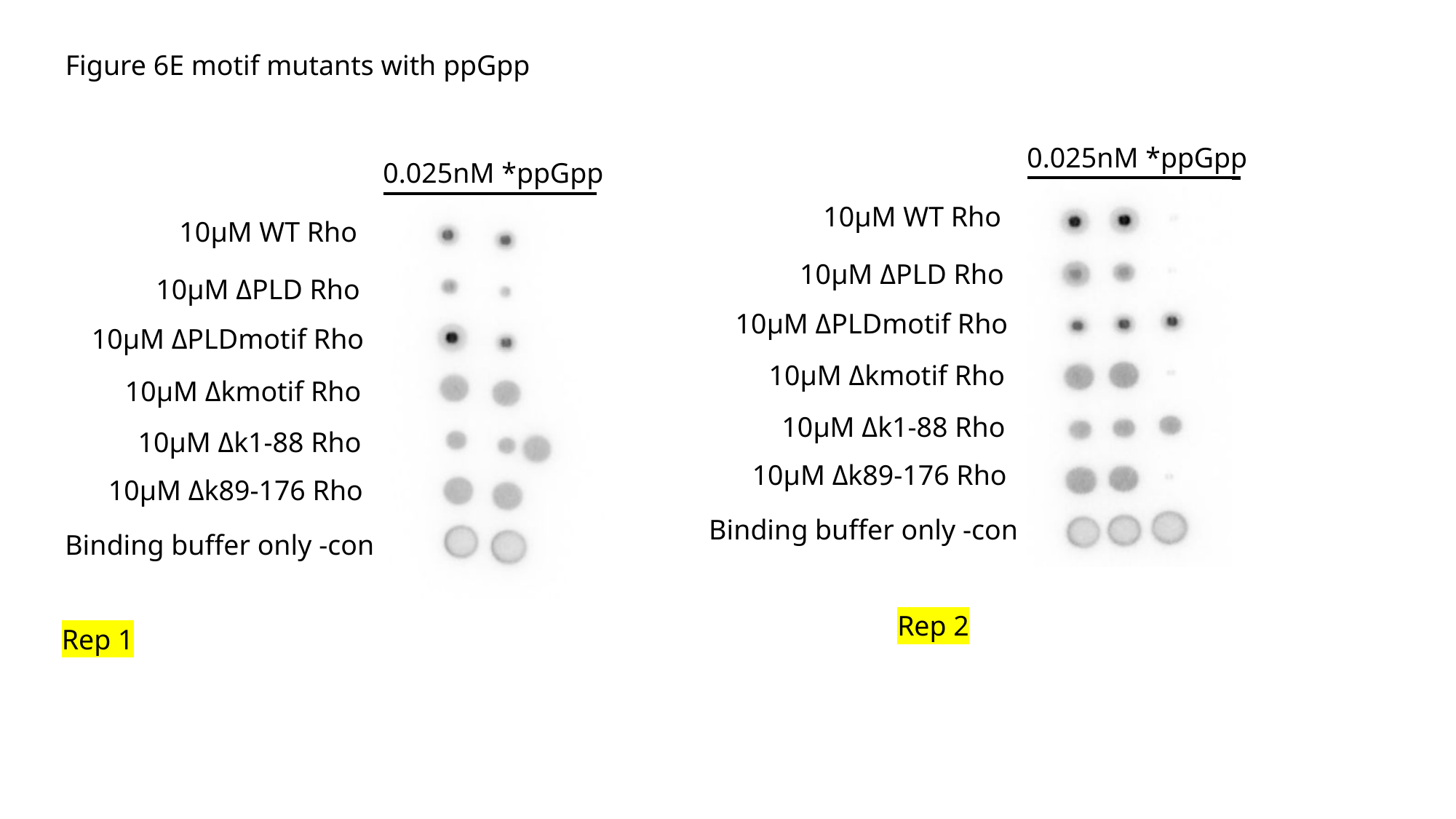

Figure 6E motif mutants with ppGpp
0.025nM *ppGpp
0.025nM *ppGpp
10μM WT Rho
10μM WT Rho
10μM ΔPLD Rho
10μM ΔPLD Rho
10μM ΔPLDmotif Rho
10μM ΔPLDmotif Rho
10μM Δkmotif Rho
10μM Δkmotif Rho
10μM Δk1-88 Rho
10μM Δk1-88 Rho
10μM Δk89-176 Rho
10μM Δk89-176 Rho
Binding buffer only -con
Binding buffer only -con
Rep 2
Rep 1

## Slide 2
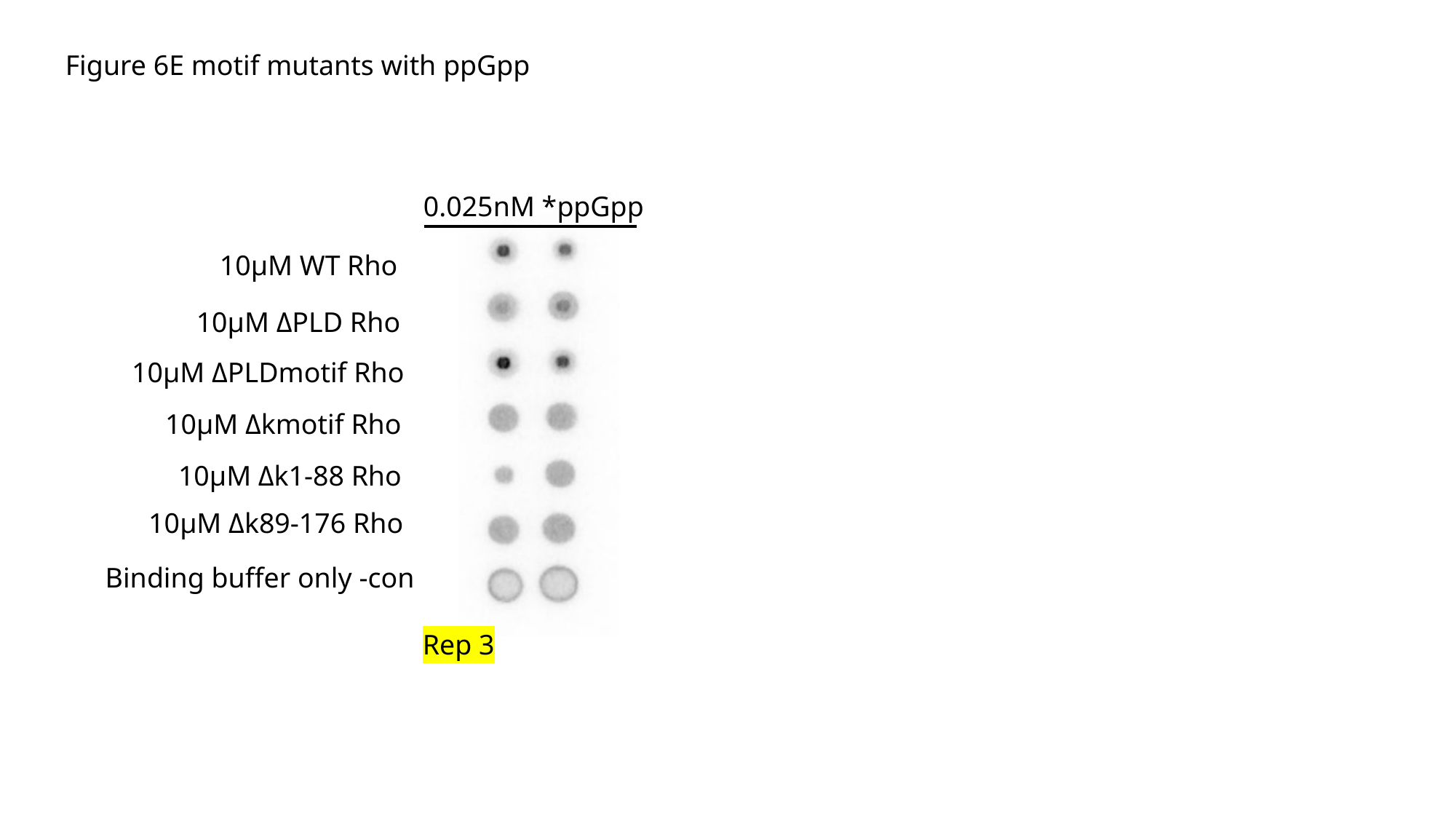

Figure 6E motif mutants with ppGpp
0.025nM *ppGpp
10μM WT Rho
10μM ΔPLD Rho
10μM ΔPLDmotif Rho
10μM Δkmotif Rho
10μM Δk1-88 Rho
10μM Δk89-176 Rho
Binding buffer only -con
Rep 3
