# Supplementary material for: Short autoinhibitory sequences control phase separation of an essential bacterial transcription termination factor
Source: EMBO J. 2026 May 11;45(12):4124–52. doi: 10.1038/s44318-026-00793-1 (PMC13269538; doi:10.1038/s44318-026-00793-1)
Supplement: Supplementary file 8 — Source data Fig. 6 [file 44318_2026_793_MOESM8_ESM.zip › Figure 6/6D/DRaCALA_triplicates.pptx]

## Slide 1
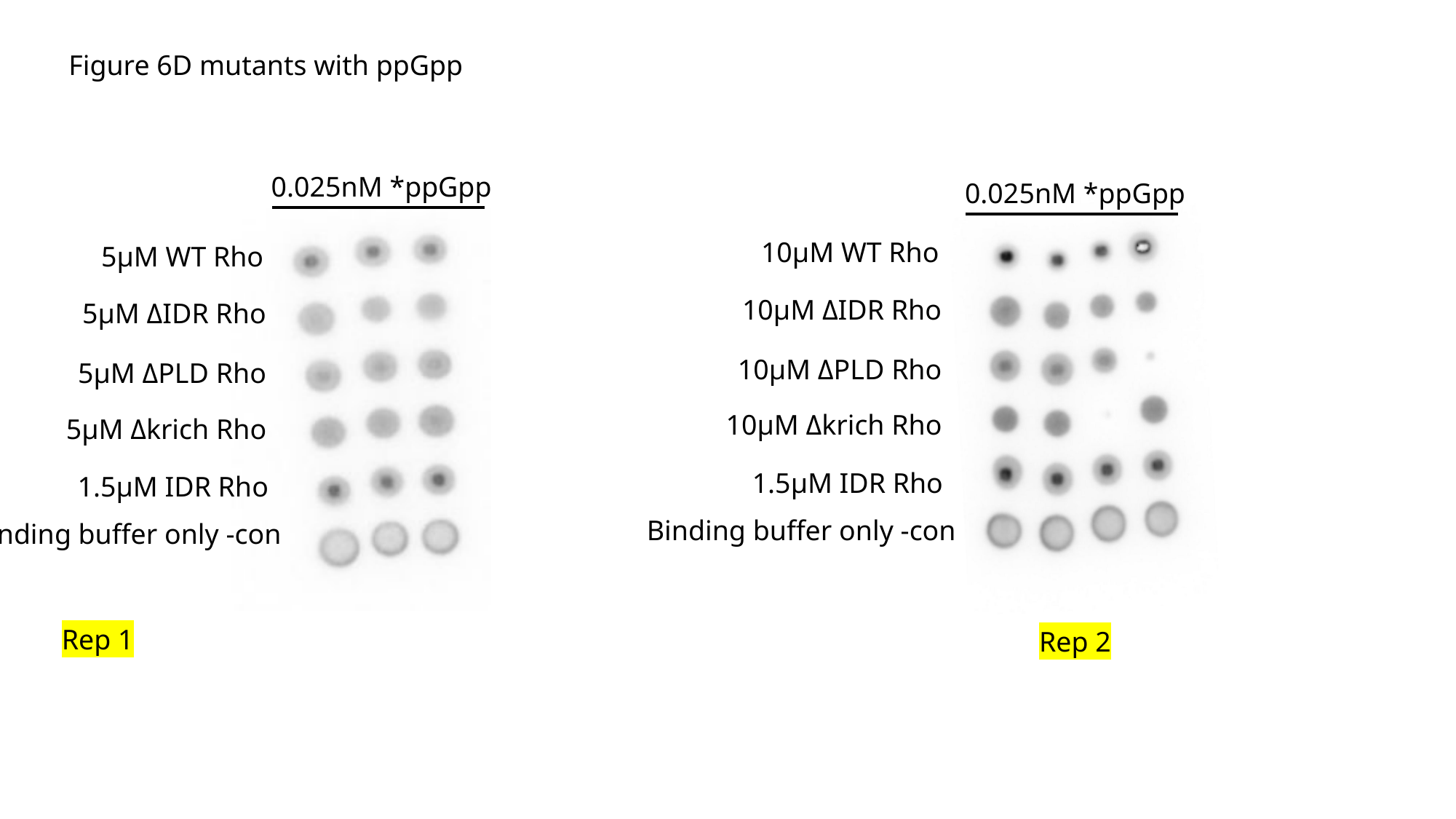

Figure 6D mutants with ppGpp
0.025nM *ppGpp
0.025nM *ppGpp
10μM WT Rho
5μM WT Rho
10μM ΔIDR Rho
5μM ΔIDR Rho
10μM ΔPLD Rho
5μM ΔPLD Rho
10μM Δkrich Rho
5μM Δkrich Rho
1.5μM IDR Rho
1.5μM IDR Rho
Binding buffer only -con
Binding buffer only -con
Rep 1
Rep 2

## Slide 2
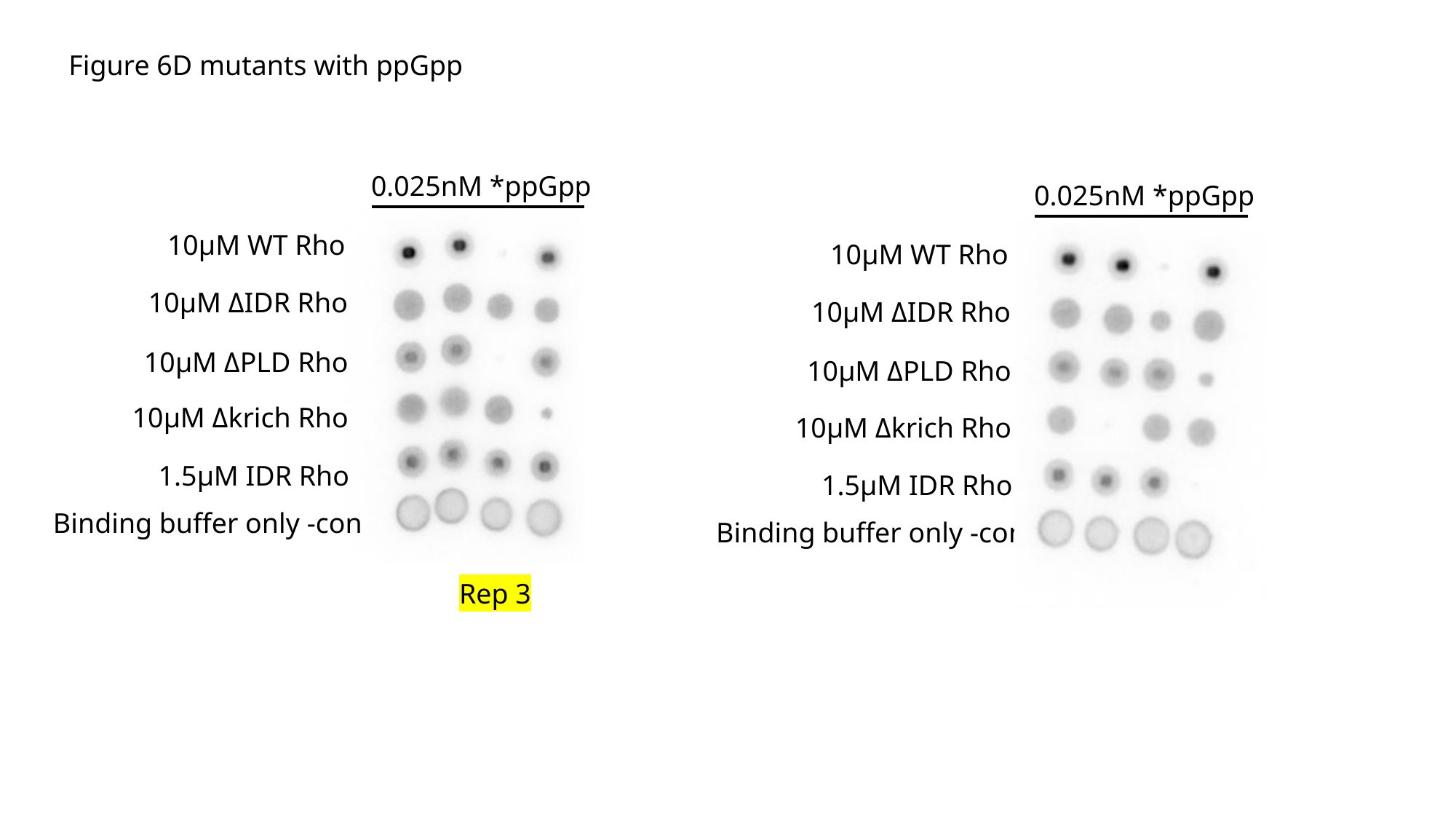

Figure 6D mutants with ppGpp
0.025nM *ppGpp
0.025nM *ppGpp
10μM WT Rho
10μM WT Rho
10μM ΔIDR Rho
10μM ΔIDR Rho
10μM ΔPLD Rho
10μM ΔPLD Rho
10μM Δkrich Rho
10μM Δkrich Rho
1.5μM IDR Rho
1.5μM IDR Rho
Binding buffer only -con
Binding buffer only -con
Rep 3
