# Supplementary material for: Short autoinhibitory sequences control phase separation of an essential bacterial transcription termination factor
Source: EMBO J. 2026 May 11;45(12):4124–52. doi: 10.1038/s44318-026-00793-1 (PMC13269538; doi:10.1038/s44318-026-00793-1)
Supplement: Supplementary file 8 — Source data Fig. 6 [file 44318_2026_793_MOESM8_ESM.zip › Figure 6/6C/DRaCALA_triplicates.pptx]

## Slide 1
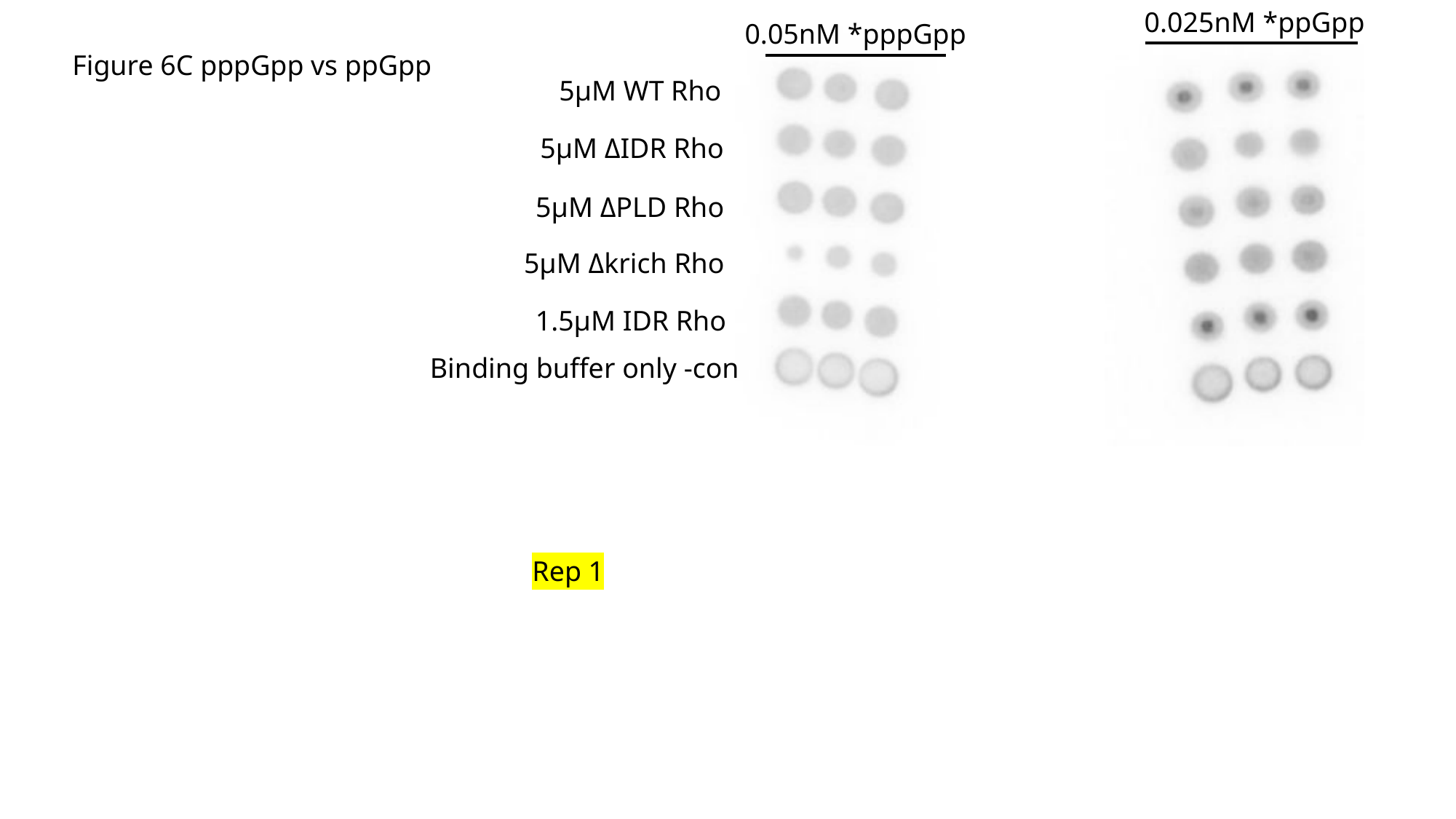

0.025nM *ppGpp
0.05nM *pppGpp
Figure 6C pppGpp vs ppGpp
5μM WT Rho
5μM ΔIDR Rho
5μM ΔPLD Rho
5μM Δkrich Rho
1.5μM IDR Rho
Binding buffer only -con
Rep 1

## Slide 2
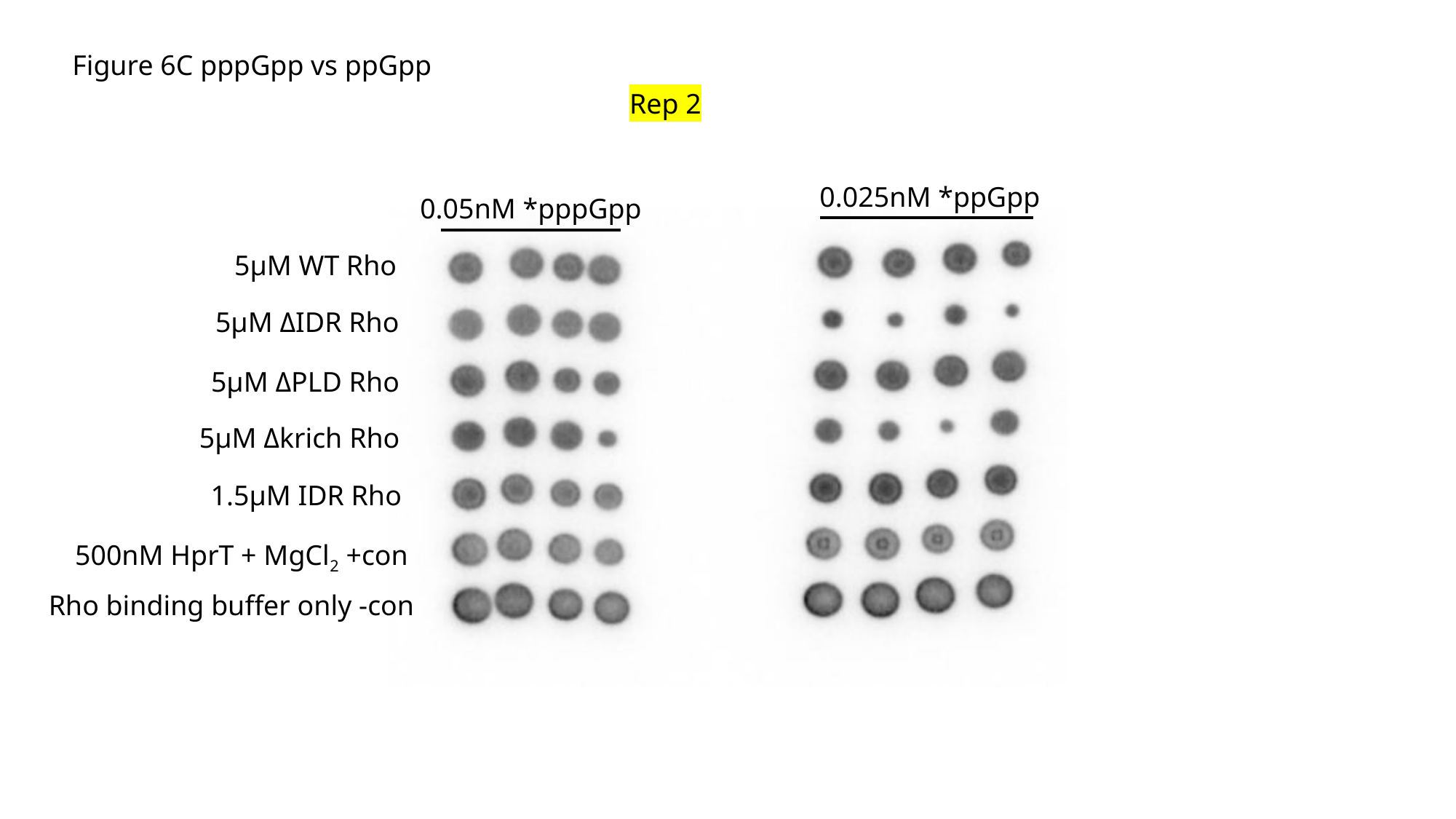

Figure 6C pppGpp vs ppGpp
Rep 2
0.025nM *ppGpp
0.05nM *pppGpp
5μM WT Rho
5μM ΔIDR Rho
5μM ΔPLD Rho
5μM Δkrich Rho
1.5μM IDR Rho
500nM HprT + MgCl2 +con
Rho binding buffer only -con

## Slide 3
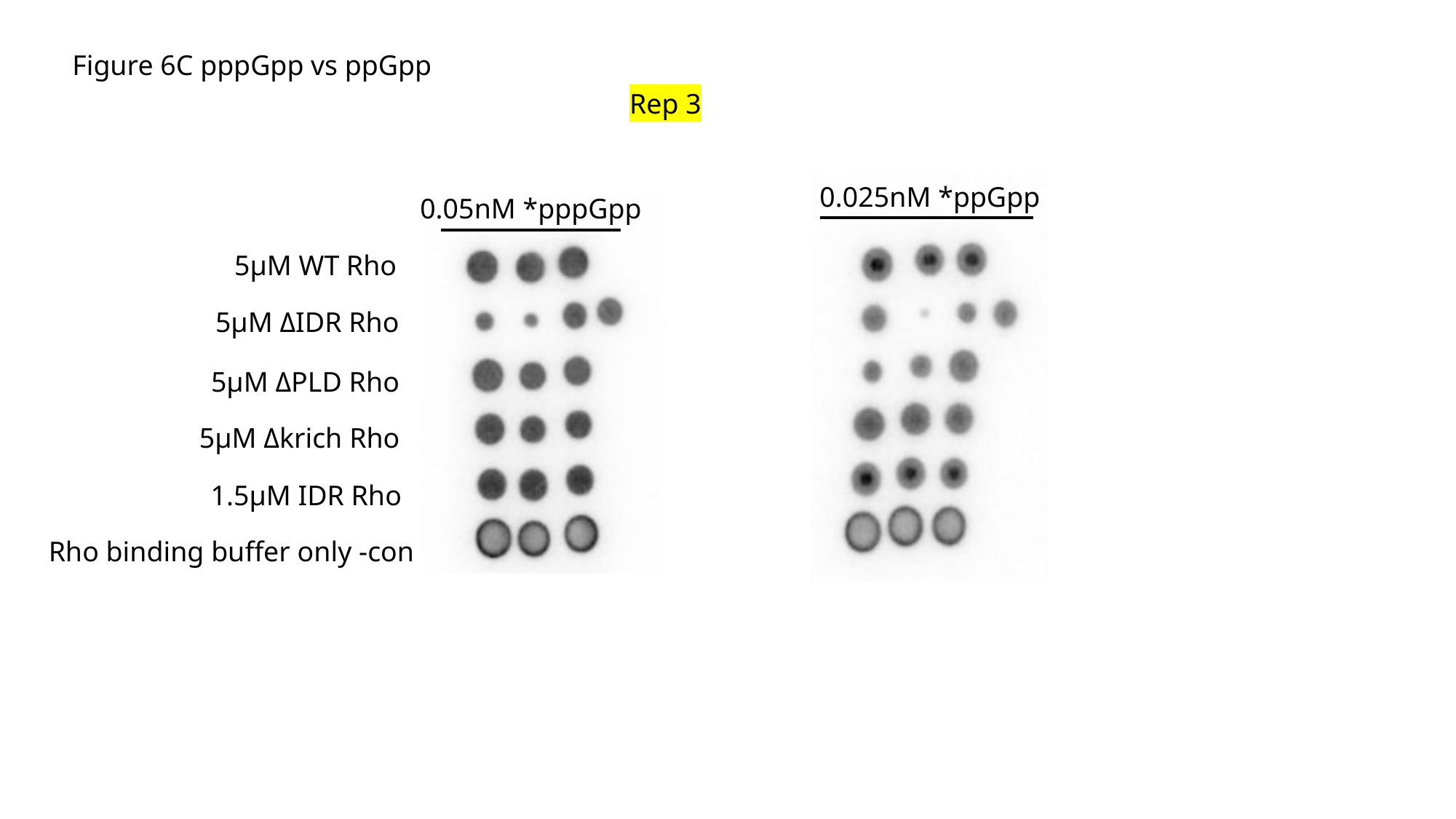

Figure 6C pppGpp vs ppGpp
Rep 3
0.025nM *ppGpp
0.05nM *pppGpp
5μM WT Rho
5μM ΔIDR Rho
5μM ΔPLD Rho
5μM Δkrich Rho
1.5μM IDR Rho
Rho binding buffer only -con
