# Supplementary material for: Short autoinhibitory sequences control phase separation of an essential bacterial transcription termination factor
Source: EMBO J. 2026 May 11;45(12):4124–52. doi: 10.1038/s44318-026-00793-1 (PMC13269538; doi:10.1038/s44318-026-00793-1)
Supplement: Supplementary file 9 — Expanded View Figures [file 44318_2026_793_MOESM9_ESM.pdf]

Expanded View Figures

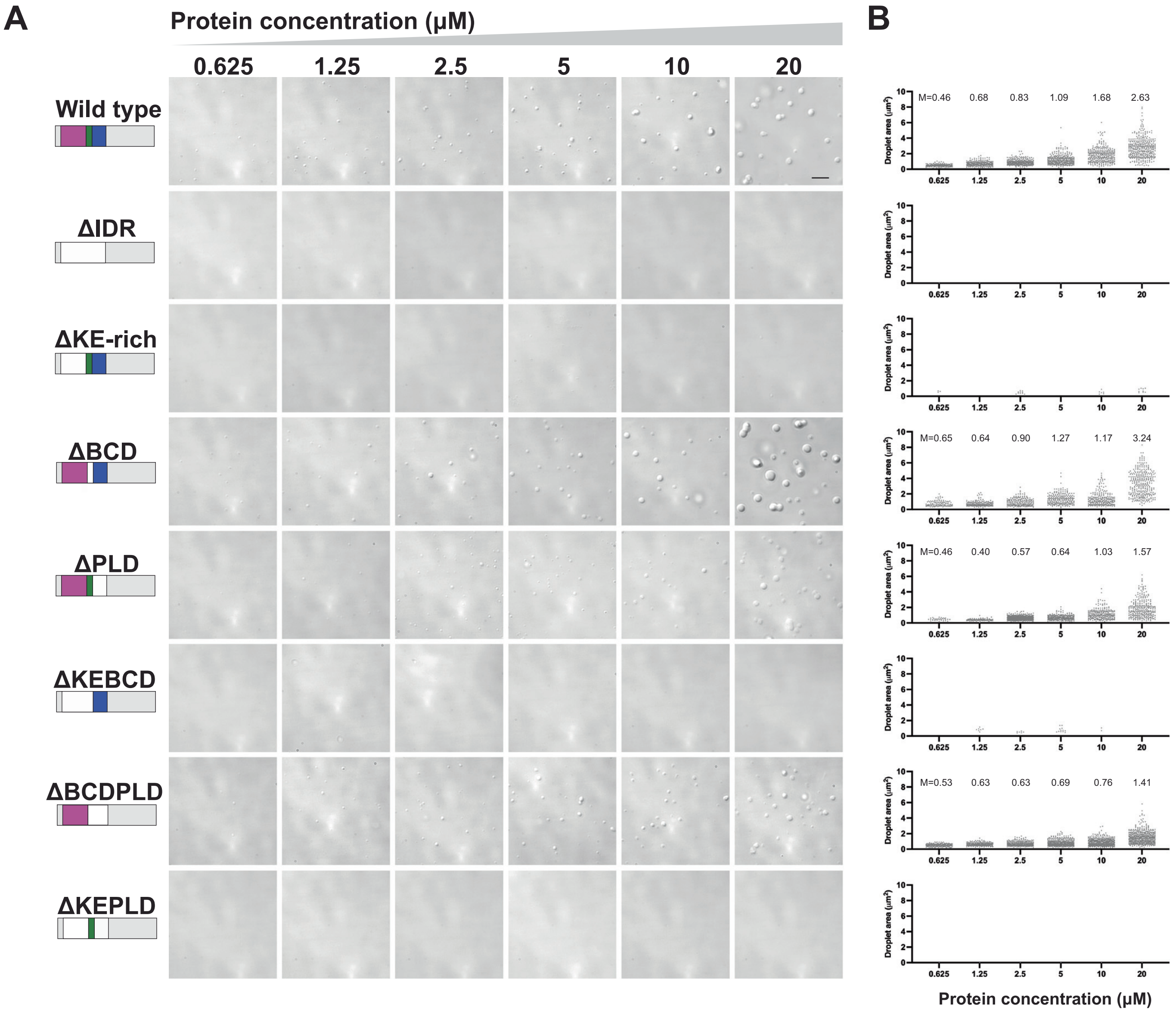

Figure EV1. *Bt*Rho variants harboring the KE-rich or PLD subdomains drive condensation.

(**A**) DIC microscopy of in vitro condensation assays of wild-type *Bt*Rho and variants at the indicated protein concentrations. The results corresponding to protein concentrations of 5, 10, and 20 μM are also shown in Fig. 2A. Three independent experiments were performed, and a representative experiment is shown. Scale bar: 5 μm. (**B**) Quantification of droplet size formed in (**A**) for each *Bt*Rho variant. For each condition, the counted droplets were from three different fields of view of the same sample. The median (M) value is also indicated for each condition.

 © The Author(s)

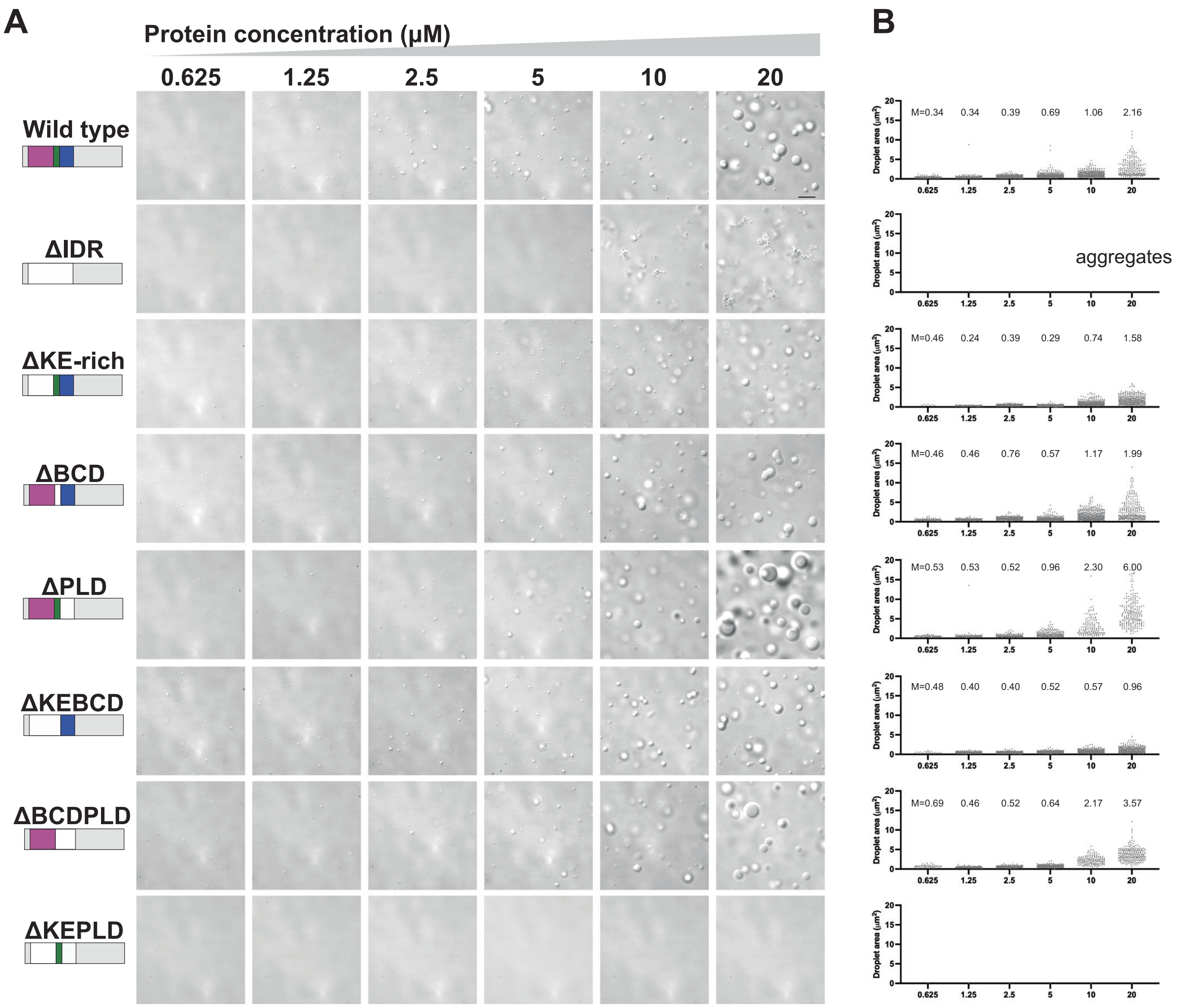

Figure EV2. *Bt*Rho variants harboring the KE-rich or PLD subdomains form large condensates in the presence of the crowding factor dextran.

(**A**) DIC microscopy of in vitro condensation assays of wild-type *Bt*Rho and variants at the indicated protein concentration and in the presence of 10% w/v dextran. The results corresponding to protein concentration 20 µM are also shown in Fig. 2A. Three independent experiments were performed, and a representative experiment is shown. Scale bar: 5µm. (**B**) Size quantification of droplets formed in (**A**) for each protein variant. For each condition the droplets counted were from 3 different fields of view of the same sample. The median (M) value is also indicated for each condition.

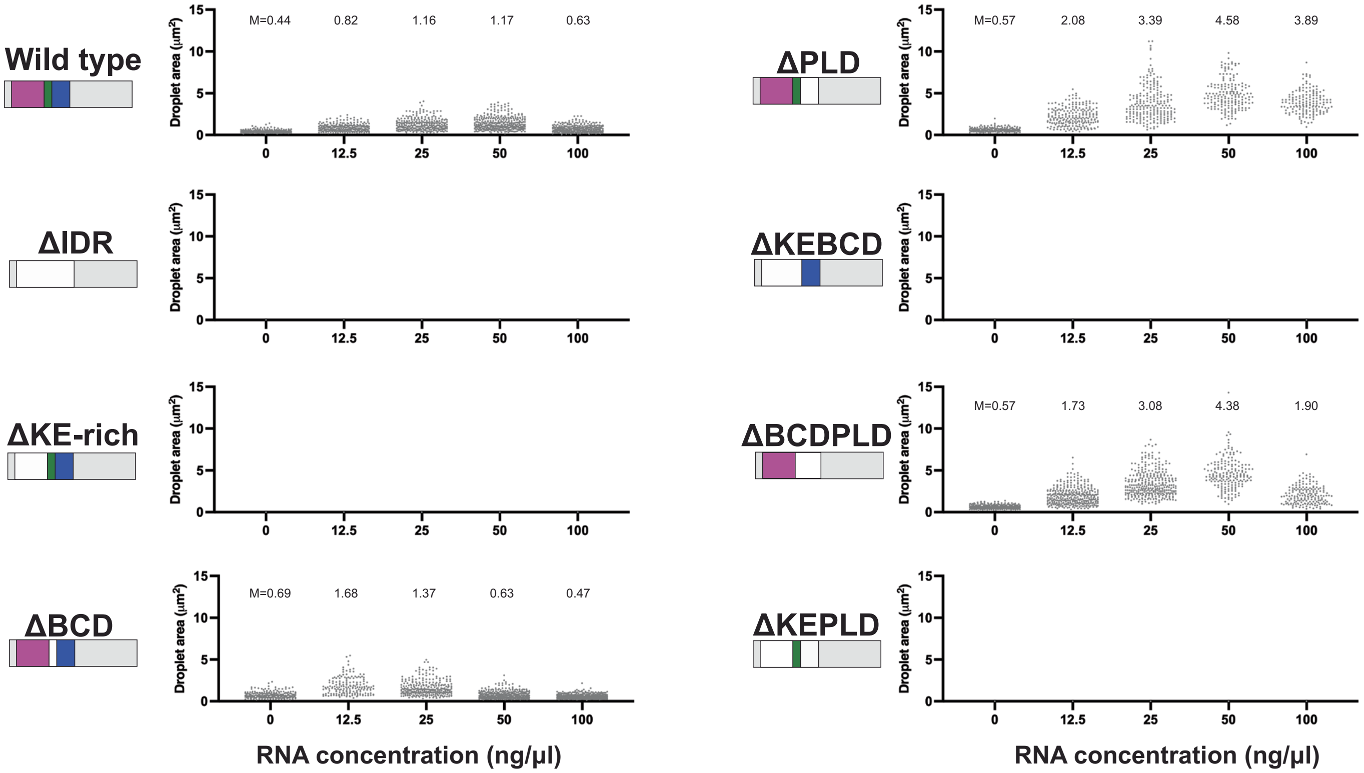

Figure EV3. The KE-rich subdomain is required for RNA-dependent *Bt*Rho condensation in vitro.

Size quantification of droplets formed in Fig. 2B for each investigated protein. For each condition the droplets counted were from three different fields of view of the same sample. The median (M) value is also indicated for each condition.

 © The Author(s)

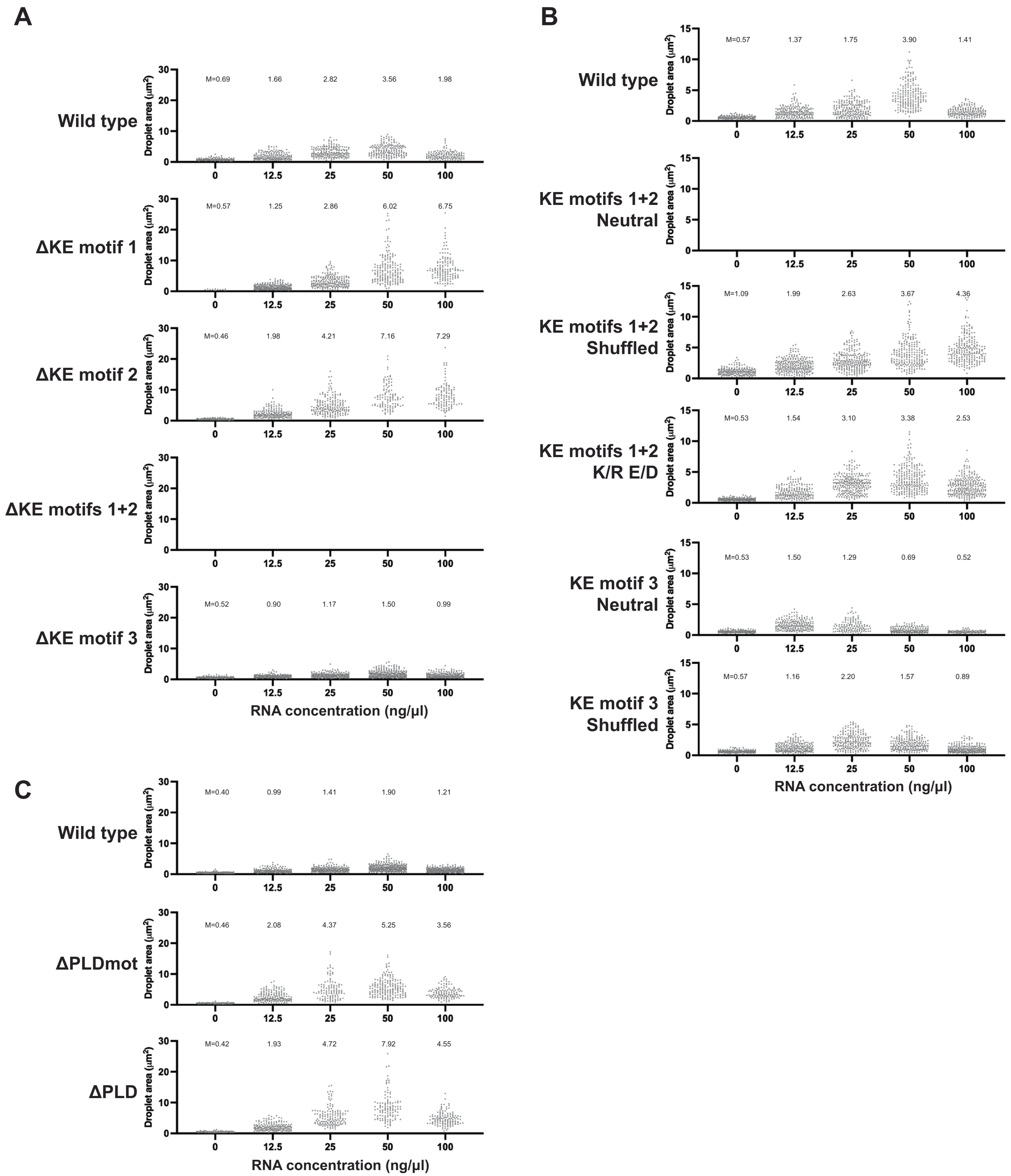

Figure EV4. Sequence motifs within the KE-rich and PLD subdomains control RNA-dependent *Bt*Rho condensation in vitro.

Size quantification of droplets formed in Fig. 3D (**A**), Fig. 3F (**B**) and Fig. 4C (**C**) for the indicated variants. For each condition, the droplets counted were from three different fields of view of the same sample. The median (M) value is also indicated for each condition.

© The Author(s)

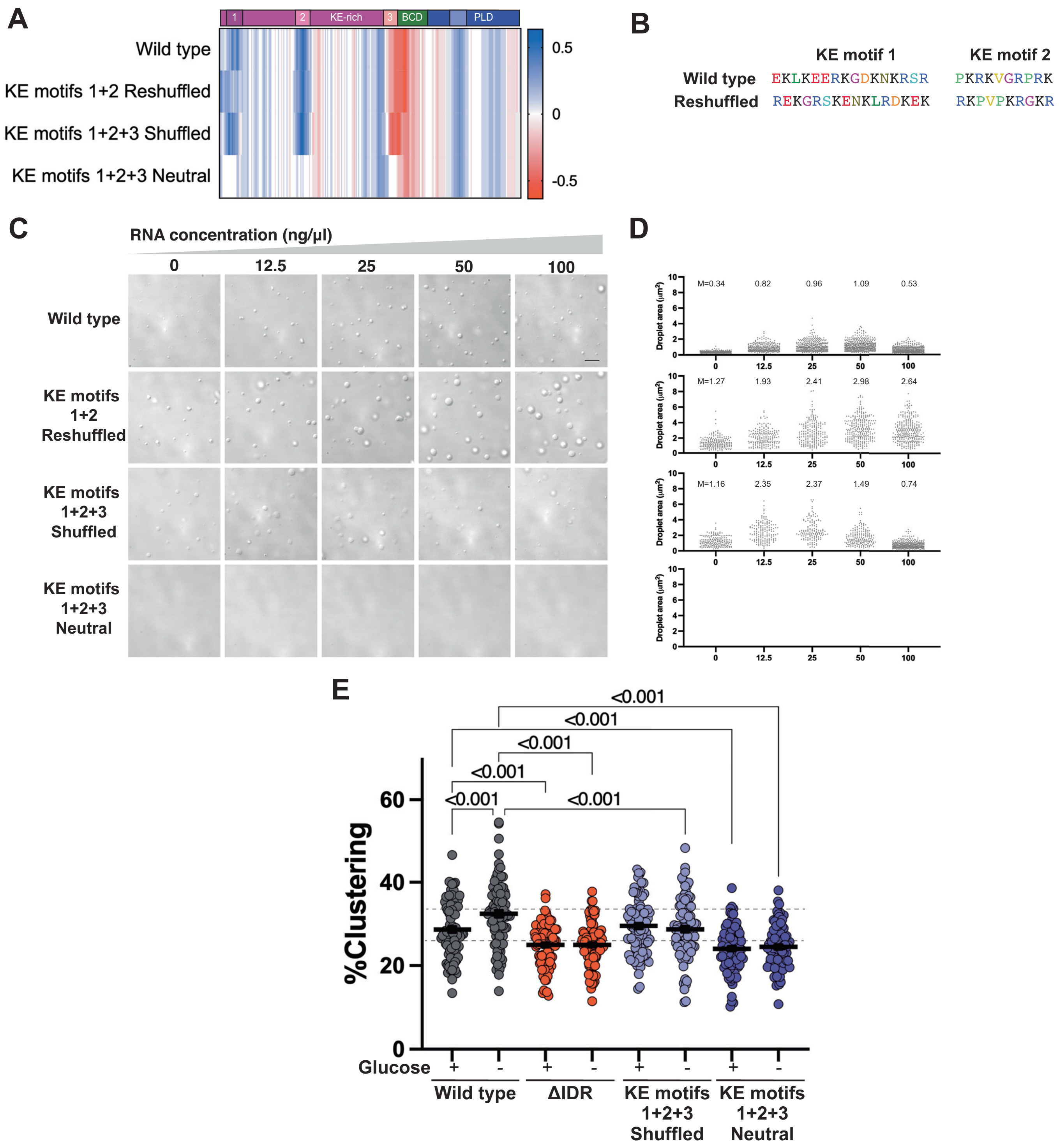
A
1
2
KE-rich
3
BCD
PLD
Wild type
KE motifs 1+2 Reshuffled
KE motifs 1+2+3 Shuffled
KE motifs 1+2+3 Neutral
0.5
0
-0.5
B
KE motif 1
KE motif 2
Wild type EKLKEERKGDKNKRSR PKRKVGRPRK
Reshuffled REKGRSKENKLRDKEK RKPVPKRGKR
C
RNA concentration (ng/µl)
0
12.5
25
50
100
Wild type
KE motifs 1+2 Reshuffled
KE motifs 1+2+3 Shuffled
KE motifs 1+2+3 Neutral
D
Droplet area (µm²)
M=0.34 0.82 0.96 1.09 0.53
M=1.27 1.93 2.41 2.98 2.64
M=1.16 2.35 2.37 1.49 0.74
E
%Clustering
<0.001
Glucose
+
-
Wild type
ΔIDR
KE motifs 1+2+3 Shuffled
KE motifs 1+2+3 Neutral

 © The Author(s)

Figure EV5. KE motifs 1, 2, and 3 control *Bt*Rho condensation.

(**A**) Heatmap of the amino acid charge per residue (EMBOSS (Rice et al, 2000), sliding window 11 amino acids) for wild-type *Bt*Rho and the indicated variants. (**B**) Amino acid sequence of wild-type KE motifs 1 and 2 and of the indicated variants. (**C**) DIC microscopy of in vitro condensation assays of wild-type *Bt*Rho and variants (2.5 µM) in the presence of increasing RNA amounts corresponding to total RNA extract from *B. thetaiotaomicron*. Three independent experiments were performed, and a representative experiment is shown. Scale bar: 5 µm. (**D**) Size quantification of droplets formed in (**C**) for each variant. For each condition, the droplets counted were from three different fields of view of the same sample. The median (M) value is also indicated for each condition. (**E**) In vivo *Bt*Rho condensation calculated as % Clustering in *B. thetaiotaomicron* strains expressing HA-tagged versions of the indicated proteins (wild type: AK600, ΔIDR: AK602, KEmotifs 1 + 2 + 3 Shuffled: AK660, and KEmotifs 1 + 2 + 3 Neutral: AK658). Bacteria were grown in glucose (+) until mid-exponential phase and then shifted to a media without any carbon source for 30 min (−). Data points represent clustering values of individual cells from three independent experiments ($n = 90$), black bars are mean values and error bars represent SEM. Dashed lines are used as visual aids to indicate the levels of clustering for wild-type *Bt*Rho in carbon starvation and for ΔIDR *Bt*Rho. One-way ANOVA was performed, followed by pairwise comparisons between wild-type and the mutant variants for the same growth condition or between the two conditions for the same strain. P values < 0.05 are shown. Šidak's test was used to correct for multiple comparisons.

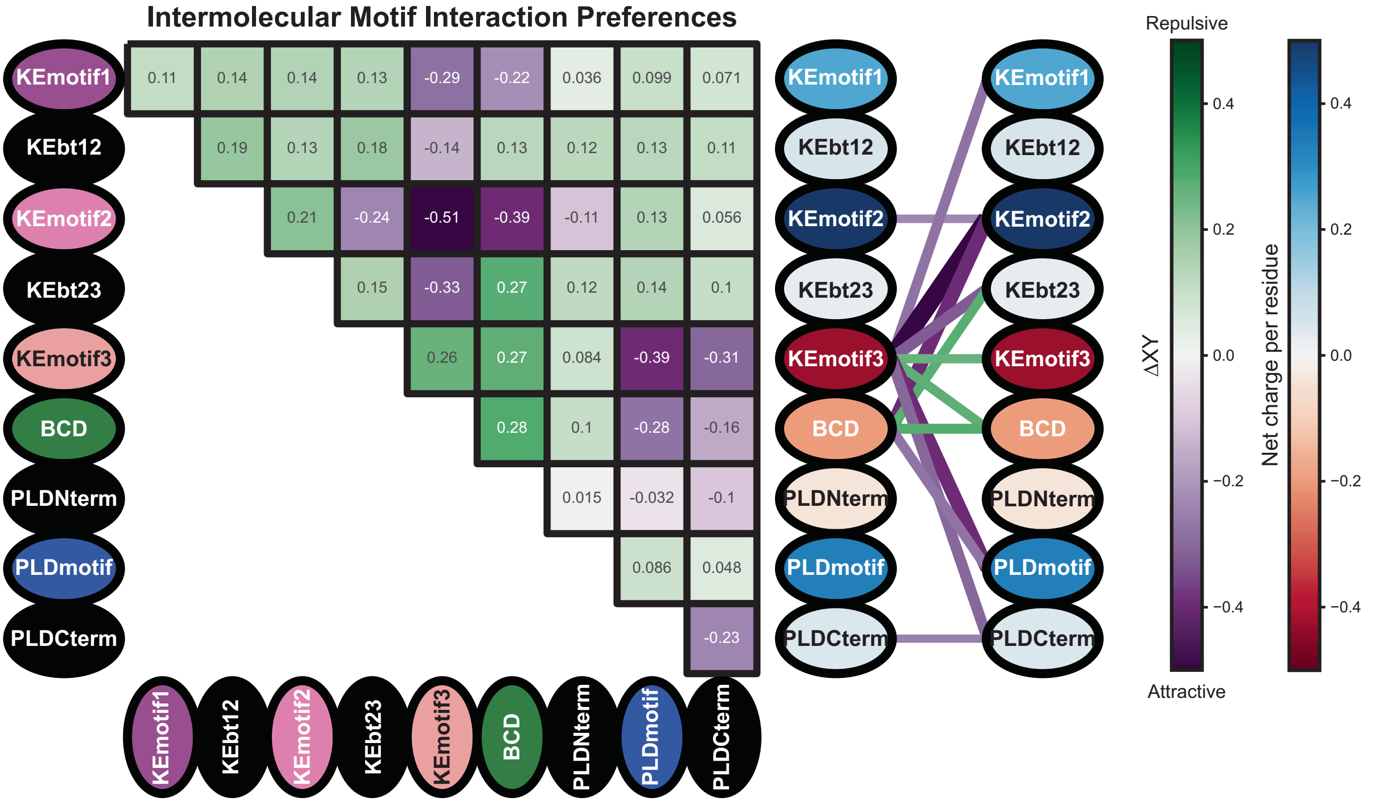

Figure EV6. Two molecule simulations reveal intermolecular interactions between the identified motifs in *Bt*Rho.

Two molecule simulations were run by splitting up the *Bt*Rho IDR into nine pieces (KE motif 1, KEbt12 (region between motifs 1 + 2), KE motif 2, KEbt23 (region between motifs 2 and 3), KE motif 3, BCD, PLD Nterm, PLDmotif, PLD Cterm) and then determining the effective interaction between each pair of pieces (ΔXY). Negative values imply attraction and positive values imply repulsion. Lines are drawn between two pieces if |ΔXY| ≥ 0.23 and weighted by their interaction strength.

© The Author(s)

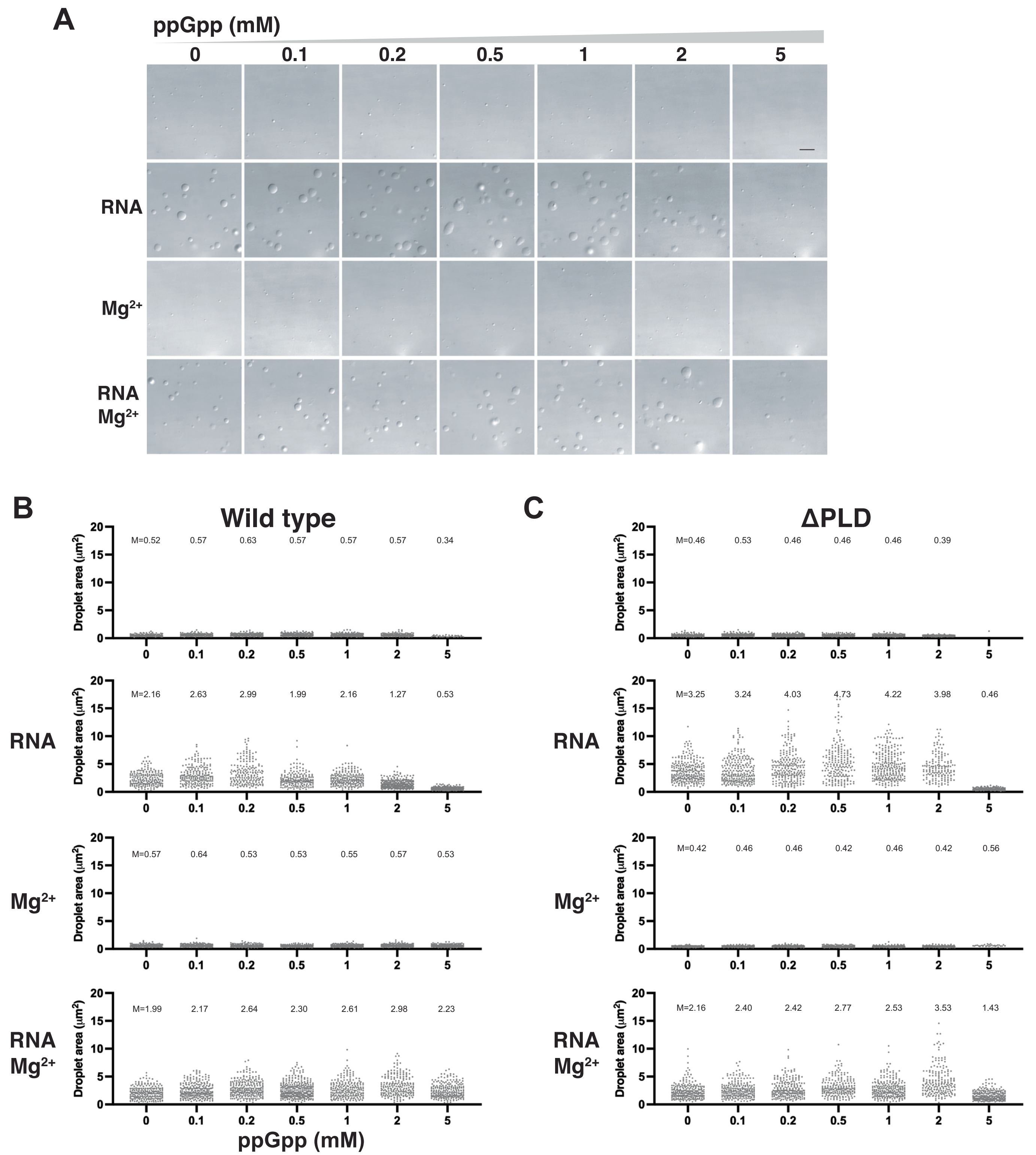

Figure EV7. ppGpp promotes RNA-dependent *Bt*Rho phase separation in vitro.

(**A**) DIC microscopy of in vitro condensation of the *Bt*Rho ΔPLD protein (2.5 μM) in the presence of increasing ppGpp concentration, RNA (12.5 ng/μl), and/or $MgCl_2$ (2.5 mM). Three independent experiments were performed, and a representative experiment is shown. Scale bar: 5 μm. (**B**) Size quantification of droplets formed in Fig. 6F for each condition. (**C**) Size quantification of droplets formed in (**A**) for each condition. For each graph, the droplets counted were from three different fields of view of the same sample. The median (M) value is also indicated for each condition.

© The Author(s)
