# Supplementary material for: Short autoinhibitory sequences control phase separation of an essential bacterial transcription termination factor
Source: EMBO J. 2026 May 11;45(12):4124–52. doi: 10.1038/s44318-026-00793-1 (PMC13269538; doi:10.1038/s44318-026-00793-1)
Supplement: Supplementary file 2 — Peer Review File [file 44318_2026_793_MOESM2_ESM.pdf]

THE EMBO JOURNAL

Short autoinhibitory sequences control phase separation of an essential bacterial transcription termination factor

Emilia Krypotou, Kiersten Ruff, Leah McKinney, Guy Townsend, Jue Wang, Rohit Pappu, and Eduardo Groisman *Corresponding author(s): Eduardo Groisman (eduardo.groisman@yale.edu) , Rohit Pappu (pappu@wustl.edu)*

Review Timeline:

Submission Date:	28th Aug 25
Editorial Decision:	23rd Oct 25
Revision Received:	23rd Jan 26
Editorial Decision:	17th Mar 26
Revision Received:	24th Mar 26
Accepted:	8th Apr 26

Editor: Cornelius Schneider

Transaction Report:

(Note: With the exception of the correction of typographical or spelling errors that could be a source of ambiguity, letters and reports are not edited. Depending on transfer agreements, referee reports obtained elsewhere may or may not be included in this compilation. Referee reports are anonymous unless the Referee chooses to sign their reports.)

1st Editorial Decision **23rd Oct 2025**

Dear Prof. Groisman,

Thank you for submitting your manuscript for consideration by the EMBO Journal. It has now been seen by three referees whose comments are shown below.

Thank you also for our constructive discussions on possible revision experiments. Given the referees' positive recommendations, I would like to invite you to submit a revised version of the manuscript, addressing the comments of all three reviewers as discussed during our meeting. I should add that it is EMBO Journal policy to allow only a single round of revision, and acceptance of your manuscript will therefore depend on the completeness of your responses in this revised version.

When preparing your letter of response to the referees' comments, please bear in mind that this will form part of the Review Process File, and will therefore be available online to the community. For more details on our Transparent Editorial Process, please visit our website: https://www.embopress.org/page/journal/14602075/authorguide#transparentprocess

We generally allow three months as standard revision time. As a matter of policy, competing manuscripts published during this period will not negatively impact on our assessment of the conceptual advance presented by your study. However, we request that you contact the editor as soon as possible upon publication of any related work, to discuss how to proceed. Should you foresee a problem in meeting this three-month deadline, please let us know in advance and we may be able to grant an extension.

Thank you for the opportunity to consider your work for publication. I look forward to your revision.

Yours sincerely,

Cornelius Schneider, PhD
Editor
The EMBO Journal
c.schneider@embojournal.org

Instructions for preparing your revised manuscript:

Please make sure you upload a letter of response to the referees' comments together with the revised manuscript.

Please also check that the title and abstract of the manuscript are brief, yet explicit, even to non-specialists.

When assembling figures, please refer to our figure preparation guideline in order to ensure proper formatting and readability in print as well as on screen:
https://bit.ly/EMBOPressFigurePreparationGuideline
See also guidelines for figure legends: https://www.embopress.org/page/journal/14602075/authorguide#figureformat

At EMBO Press we ask authors to provide source data for the main manuscript figures. You will receive a separate email with instructions for providing source data with your revised manuscript, including how to upload and organize the files.

IMPORTANT: When you send the revision we will require
- a point-by-point response to the referees' comments, with a detailed description of the changes made (as a word file).
- a word file of the manuscript text.
- individual production quality figure files (one file per figure)
- a complete author checklist, which you can download from our author guidelines (https://www.embopress.org/page/journal/14602075/authorguide).
- Expanded View files (replacing Supplementary Information)
Please see out instructions to authors
https://www.embopress.org/page/journal/14602075/authorguide#expandedview
- a Reagents and Tools Table as part of the Methods section, which can be downloaded from our author guidelines (https://www.embopress.org/page/journal/14602075/authorguide#structuredmethods)

Please remember: Digital image enhancement is acceptable practice, as long as it accurately represents the original data and conforms to community standards. If a figure has been subjected to significant electronic manipulation, this must be noted in the figure legend or in the 'Materials and Methods' section. The editors reserve the right to request original versions of figures and

the original images that were used to assemble the figure.

Further information is available in our Guide For Authors: https://www.embopress.org/page/journal/14602075/authorguide

We realize that it is difficult to revise to a specific deadline. In the interest of protecting the conceptual advance provided by the work, we recommend a revision within 3 months (21st Jan 2026). Please discuss the revision progress ahead of this time with the editor if you require more time to complete the revisions. Use the link below to submit your revision:

https://emboj.msubmit.net/cgi-bin/main.plex

Referee #1:

No language model was used to draft or edit the reviewer's opinion below.
Krypotou et al. study the transcription factor Rho from Bacteroides thetaitaomicron (BtRho) and dissect the sequence determinants of condensate formation in vitro and in vivo. It is a careful and thorough work of testing many deletion and substitution mutations in the IDR region of the protein. The authors conclude, that short charged conserved motifs control the condensation of BtRho. The 9 aa region (PLD motif) and KE motif 3 inhibit it, while KE motif 1 and KE motif 2 promote it and interact with RNA which is driven by their positive charges. Additionally, they test direct binding to the alarmon ppGpp and detect IDR-dependent binding. I find the work interesting, it confirms and agrees with finding regarding other phase separating systems, while providing mechanistic insights about this specific transcription factor, and how ppGpp can trigger its condensation upon glucose starvation.

Major comments:
While I was trying to follow the interpretation and arguments of the authors of their in vitro experiments, I was often puzzled eyeballing the images. Due to the lack of quantification, statements like "smaller in size but higher in number", "smaller than those formed by WT BtRho but larger than those formed by the variant...", "... resulted in a moderate increase in the size of condensates..." are difficult to follow. This is true for all in vitro figures.
The in vivo data is presented with greater rigor and the %clustering is shown for many cells backed up by statistical tests. However, it is not explained in the text (not in methods) what is %clustering, how was that quantified. Also, the authors refer to change in condensate size, which is not possible to see from these plots at all.
I am not convinced that KE motif 3 regulates condensation (page 11). In fact, Fig 3G shows that it does not in vivo. And even the authors say at the end of the section: RNA interactions with KE motif 1 abd KE motif 2 are the main drivers of BtRho condensation in vivo. So is there a consistent discrepancy between the in vivo and in vitro conclusions?
I am also puzzled by the shuffling variants increasing condensation. Wouldn't that mean that there is no sequence specificity required for the KE 1 and KE 2 motifs?
Why do the mutants loose the ability to respond to glucose in Figure 3?

Minor comments:
Abstract: I would recommend revising the first two sentences. The first one is too long and the second one is quite repetitive.
Introduction: the relationship between condensates and membraneless bodies is not defined.
Please indicate in Figure 1 the residue numbers of the different regions and indicate whenever only a fragment is shown. The alignment is panel C is misleading that it starts with residue number 1. A supplementary table with all fragments and mutants' regions and sequences would be helpful. Also, I would use a color that represents the amino acid type in panel C.
Consider splitting the second chapter of the Results into two, since it is very long.
Figure 5F. I am not sure what better, similar or worse than WT means here for each of the experiments. I would revise and make it clearer. Having a summary figure is a good idea, but I would add it to Figure 7 instead since it is not only information about simulations, but almost all experiments. Actually, the pppGpp experiments could be added to it as well.
The schematics in Figure 7. The pink colors look the same when printed. Consider a stronger color gradient to distinguish the KE motifs. It is not intuitive what interactions would drive condensation in nutrient conditions since no inter-chain interactions are indicated? Could simulations help suggesting what would drive condensation?
I suggest to add a few sentences on the alarmon (p)ppGpp and its relevance to stress response and btRho activation as an introduction at the beginning of the section.
DRaCALA experiments: why not showing all three replicates? The captions state, that three independent experiments were performed and a representative image is shown. There would be enough space to show all three in Figures 6CD and EV4.

Non-essential suggestions:
Would it be possible to perform simulations including RNA? The fact that the FINCHES predictions suggest that the positively charged motifs should bind RNA and the negatively charged ones agrees with purely electrostatic considerations. Would that look different for poly-rG vs. poly-rU? Since the alarmon sequence and GTP binding was shown to be important. Or there is no expectation on sequence-specificity?

Referee #2:

In this manuscript, Krypotou and colleagues expand on their groundbreaking discovery that the transcription termination factor Rho from Bacteroides thetaiotaomicron (BtRho) forms biomolecular liquid-liquid phase-separated (LLPS) condensates, which are critical for the fitness of the bacterium in the mammalian gut (Science 2023, PMID: 36927025). This study presents the only well-characterized example of Rho activity -critical across many bacteria- being regulated through LLPS condensation, though a recent preprint suggests a potentially similar mechanism in mycobacterial Rho (PMID: 40909676), albeit with less comprehensive evidence.
Through a rigorous, multidisciplinary approach-combining phylogenetic analyses, molecular dynamics simulations, and extensive in vitro/in vivo characterization of BtRho domain mutants-the authors elucidate the molecular mechanisms underlying BtRho condensation. They identify conserved domains within an intrinsically disordered region (IDR) of BtRho that both mediate and regulate LLPS condensation in an RNA-dependent manner.
Given that BtRho condensation is enhanced under carbon starvation in vivo (Science 2023), the authors further hypothesized that the alarmone ppGpp, which accumulates under such conditions, may also promote BtRho condensation. While they provide some indirect support for this idea, this remains the least substantiated (yet potentially most exciting) conclusion in an otherwise thorough and well-designed study. This hypothesis is particularly intriguing in light of recent findings in E. coli, where ppGpp inactivates Rho through a distinct 'hyper-oligomerization' mechanism (PMID: 39952913). If confirmed, the present work would not only generalize the role of ppGpp in regulating Rho activity but also demonstrate that such stress-related regulation can manifest through diverse molecular strategies.
Given the broad, potential impact of these findings, I recommend the authors address the following points in a revised manuscript:
Main Points:
1) ppGpp stimulation: The proposed role of ppGpp in BtRho condensation is based on two observations: first, carbon starvation (absence of glucose) fails to stimulate BtRho condensate formation in a ppGpp^0 strain; second, BtRho can bind ppGpp in vitro. However, direct evidence that ppGpp stimulates BtRho condensation is lacking. The in vivo effects could arise from any of the numerous metabolic alterations in the ppGpp^0 background, while ppGpp binding may not be specific -GTP, for example, effectively competes with ppGpp (Fig. EV4B) - nor responsible for condensate formation. Notably, the authors extensively characterized BtRho condensation in vitro using DIC microscopy, yet they did not test ppGpp's effect under these controlled conditions. A direct demonstration of ppGpp's stimulatory effect on BtRho condensation (with or without RNA) would significantly strengthen their conclusion, linking a critical bacterial alarmone to a novel regulatory mechanism.
2) Additional controls, such as testing other nucleotides (particularly ATP) via DIC microscopy and DRaCALA blotting, or the effect of ppGpp on Rho-dependent transcription termination (e.g., using the in vitro assay from their original work: PMID: 36927025) would further clarify the specificity and functional relevance of ppGpp binding to BtRho's IDR.

3) Role of Rho Oligomerization: What are the contributions of Rho's C-ter domain and Rho oligomerization (e.g., hexamer formation) to condensate formation? Can the IDR alone (or with RNA) form condensates or is the high local concentration/proximity of IDRs in Rho hexamers critical for LLPS condensation?

Minor points:
4) Phenotypic Heterogeneity: The extensive in vivo work-both here and in the previous study-reveals significant heterogeneity among Bacteroides cells under any given condition. How were cells selected for imaging? Is this heterogeneity linked to experimental limitations, or does it reflect genuine phenotypic diversity? If the latter, how do the authors interpret this variability? Could it provide some advantage at the population level?
5) Figure 2C: The figure legend mentions error bars representing SEM, but none are visible in Figure 2C. This discrepancy should be clarified.

Referee #3:

The Groisman group has previously shown that the transcription termination factor Rho from Bacteroides thetaiotaomicron (BtRho) phase separates in response to carbon starvation, and that the 303-residue IDR at its N-terminus, which is absent in Rho from most studied model bacteria, is necessary for condensation both in vivo and in vitro. The group has further shown that phase separation of BtRho increases its transcription termination activity and promotes fitness of Bt in the mammalian gut.
In the current manuscript, the Groisman group and collaborators set out to reveal the specific protein sequences and the small molecules that control BtRho condensation. They identified short, charged or conserved motifs within the IDR that govern BtRho condensation, established specific interactions that these motifs mediate, both within the IDR itself and with RNA, suggest that the alarmone ppGpp is essential for the carbon starvation-induced condensation of BtRho, and showed that ppGpp has the capacity to bind directly to BtRho in a manner that depends on the IDR.
The manuscript reports novel and original findings, is well written and should be suitable for publication in EMBO J after addressing the following points below. Since the manuscript contains a substantial body of work, in addition to suggesting experimental approaches to address the different points, I leave it up to the authors to state the limitations in the text, instead of

addressing them experimentally.
Major comments:
1. Revealing specific protein sequences that control condensation is of importance, since they may provide insights on the mechanism that drives phase separation in general. However, the authors declare that they revealed also a molecule that control the process and are necessary for it, that is, ppGpp. In my opinion, the latter finding is the "cherry" that enhances the attraction of this manuscript. Therefore, I find the lack of in vitro studies to characterize the involvement of ppGpp in condensation as a drawback. This lack stands out, since the balance between the in vivo and in vitro results is one of the strengths of the manuscript, and the exception is the claim regarding the essentiality of ppGpp for condensation.
Indeed, binding assays of ppGpp to BtRho and its derivatives (Figure 6C) indirectly supports the notion that the alarmone is involved in condensation. However, whereas RNA is shown to drive BtRho condensation in vitro without ppGpp (Figures 2B, 3D, 3F, 4C), ppGpp is shown to be absolutely required for condensation in vivo (Figure 6A). A discrepancy between in vitro and in vivo observations regarding ppGpp effect was documented (e.g., Carmona et al., J. Bact. 2000), suggesting that the cellular environment, other regulatory networks and the cytoplasmic crowding influences regulation by ppGpp in ways that may not be fully mimiced by in vitro experiments (the latter is supported by the enhancement of BtRho condensation by the crowding agent dextran). Yet, in other studies, in vitro assessment of ppGpp effect supported the in vivo results, although not exactly mirroring them (e.g., Vrentas et al, Genes Dev. 2005), suggesting that such studies are worth trying.
This gap in the manuscript can be addressed by the authors experimentally either by measuring the binding affinity between BtRho and an RNA ligand in the absence and presence of ppGpp by a quantitative method, such as ITC or MST. This will provide a direct mechanistic link between RNA and ppGpp and resolve the apparent contradiction between the in vitro and in vivo findings. Alternatively, the authors can perform in vitro condensation assays with BtRho WT and variants lacking the KE or PRD domains in the presence of physiological concentrations of ppGpp. This approach will directly test if ppGpp is required for or enhances RNA-driven condensation also in vitro. If the authors decide not to resolve the discrepancy experimentally, they should clearly acknowledge this limitation in the Discussion. If the authors attempt/ed to test the effect of ppGpp in vitro and could not reproduce the in vivo results, reporting this unsuccessful attempt is important for other investigators.
Additionally, since the authors suggest a novel role for ppGpp, they should mention that the molecular mechanism by which ppGpp binding leads to condensation remains to be elucidated.
2. The authors focus on very specific protein motifs that mediate RNA-driven phase separation. In sharp contrast, they used total RNA extract from Bt for the in vitro experiments (Figures 2B, 3D, 3F, 4C). The problem of using total RNA mix is that rRNAs and tRNAs are the most abundant transcripts and, hence, may take over and lead to conclusions that are not physiologically relevant.
Also the use of total RNA extract precludes the possibility to draw conclusions about the interactions between BtRho motifs and RNA at a sequence level. In light of studies that highlighted the importance of specific motifs in both proteins and RNAs for co-phase separation, I suggest that the authors perform control in vitro condensation assays using defined ligands, such as poly A or poly U, or, better, the typical BtRho binding sites, assuming that some are known or can be identified by a relatively simple assay. Again, the least that the authors should do is to acknowledge this limitation by stating that their RNA mixture is dominated by interactions with abundant rRNA species.
Minor comments:
1. The authors do not provide direct evidence that the KE-rich and PLD subdomains alone are sufficient for condensation. Reconstituting the system with the synthesized subdomains or with minimal components (subdomains, synthetic RNA, potential cofactors) will address this limitation. Addressing this limitation in the text will require replacing "sufficient for condensation" with "play critical role in (or are important for) condensation".
2. Still about the in vitro system: The authors should explain why they chose certain protein concentrations and buffer conditions and how they relate to the cellular environment. My suggestion is that the authors also perform in vitro condensation assays with cell lysates to better imitate physiological conditions. This is relevant also for Major Point 1 (re ppGpp).
3. Temporal information is missing: Figure 2C shows the results 30 minutes post-starvation, neglecting possible differences in assembly or disassembly kinetics between the variants. It would be good to add a time-course analysis of condensate formation by fixing samples at various timepoints.
4. While the data in Figures 3D and 3E strongly suggest that the two KE motifs mediate RNA-dependent condensation, evidence for direct interaction with the RNA, such as EMSA, is lacking. A test that compares WT to variants with the KE charge neutralized would provide evidence for direct binding of the KE motifs to RNA. Otherwise, acknowledging in the text that direct binding remains to be biochemically confirmed is needed.

Yale SCHOOL OF MEDICINE

Department of Microbial Pathogenesis

Boyer Center for
Molecular Medicine

295 Congress Ave, Room 354D
New Haven CT 06519
203-737-7940

January 23, 2025

Cornelius Schneider, PhD
Editor
The EMBO Journal

Re: EMBOJ-2025-122310

Dear Cornelius,

Attached please find our revised manuscript titled "Short autoinhibitory sequences control phase separation of an essential bacterial transcription factor" in which we incorporated the valuable suggestions made by you and the three reviewers.

Thank you for submitting your manuscript for consideration by the EMBO Journal. Please find enclosed the comments of all three referees whom we had asked to evaluate the manuscript. As you can see from the reports all referees think that the topic is interesting and that the overall quality of the manuscript is high. However, referees #2 and #3 also think that the characterization of the role of ppGpp in the formation of Rho condensates is premature and think that the manuscript would benefit from a detailed characterization. We agree with the referees and think that these points should be addressed experimentally and that textual edits alone would not be sufficient.
All together I think that a revised manuscript addressing all the concerns raised by the three referees would be a very good fit for the EMBO Journal but I am concerned that the requested revisions are rather extensive and might go beyond the single round of revision of 3-6 month which usually allow for at The EMBO Journal. I think it might be best to discuss potential revisions via e-mail or videoconferencing whichever you would prefer.
Yours sincerely,
Cornelius Schneider, PhD
Editor
The EMBO Journal
c.schneider@embojournal.org

As discussed, our manuscript provides a most exhaustive analysis of biocondensate formation by a protein harboring an unusually large intrinsically disordered region (IDR). By carrying out a phylogenetic analysis and by performing *in vivo* and *in vitro* analysis of a large number of mutants, we unraveled the role that different regions of the unusually large IDR play in condensate formation. Our research identifies several short sequence motifs that govern the carbon starvation-triggered condensation of the *Bt*Rho protein. Most importantly, we identified short sequences that play distinct roles in *Bt*Rho condensation in the natural context of the full-length protein (i.e., as opposed to investigating the role of the short sequences in the isolated IDR). This analysis is complemented with our *in silico* analysis defining homotypic interactions between the identified sequences as well as heterotypic ones with RNA. Thus, our manuscript establishes how short specific amino acid sequences within an IDR can drive the formation of biological condensates.

The significance of our findings is highlighted by the referees. For example, referee #2 states "**Through a rigorous, multidisciplinary approach-combining phylogenetic analyses, molecular dynamics simulations, and extensive in vitro/in vivo characterization of BtRho domain mutants-the authors elucidate the molecular mechanisms underlying BtRho condensation.**". And referee #3 states "T**he manuscript reports novel and original findings, is well written and should be suitable for publication in EMBO J after addressing the following points below. Since the manuscript contains a substantial body of work, in addition to suggesting experimental approaches to address the different points, I leave it up to the authors to state the limitations in the text, instead of addressing them experimentally.**".

As discussed in detail below, our revised manuscript includes new data (generated since the original submission) that directly addresses the role played by ppGpp on *Bt*Rho condensation and how this role is impacted by RNA and Mg^{2+}. These data complement the results provided in the original submission demonstrating that (p)ppGpp is required for carbon starvation-promoted condensation of BtRho in an IDR-dependent manner *in vivo* in the natural bacterial host, and that ppGpp binds to the purified *Bt*Rho in an IDR-dependent manner. The new *in vitro* results establish that ppGpp directly impacts condensate formation by *Bt*Rho. Of course, ppGpp and/or pppGpp might play additional roles in the *in vivo* formation of *Bt*Rho condensates via RNAs, proteins, and/or metabolites the abundance and/or activity of which is controlled by ppGpp and/or pppGpp.

We very much appreciate the comments made by the three referees, which helped clarify the manuscript and resulted in the incorporation of new data. We plan to pursue the intriguing points raised by the referees, such as defining the RNA specificity of condensate formation by *Bt*Rho, and to report such findings in subsequent publications.

Referee #1 (Report for Author)

No language model was used to draft or edit the referee's opinion below.
Krypotou et al. study the transcription factor Rho from Bacteroides thetaitaomicron (BtRho) and dissect the sequence determinants of condensate formation in vitro and in vivo. It is a careful and thorough work of testing

many deletion and substitution mutations in the IDR region of the protein. The authors conclude, that short charged conserved motifs control the condensation of BtRho. The 9 aa region (PLD motif) and KE motif 3 inhibit it, while KE motif 1 and KE motif 2 promote it and interact with RNA which is driven by their positive charges. Additionally, they test direct binding to the alarmon ppGpp and detect IDR-dependent binding. I find the work interesting, it confirms and agrees with finding regarding other phase separating systems, while providing mechanistic insights about this specific transcription factor, and how ppGpp can trigger its condensation upon glucose starvation.

Major comments:
While I was trying to follow the interpretation and arguments of the authors of their in vitro experiments, I was often puzzled eyeballing the images. Due to the lack of quantification, statements like "smaller in size but higher in number", "smaller than those formed by WT BtRho but larger than those formed by the variant...", "... resulted in a moderate increase in the size of condensates..." are difficult to follow. This is true for all in vitro figures.
As suggested by the referee, the revised manuscript provides quantification of the *in vitro* condensate formation data.

The in vivo data is presented with greater rigor and the %clustering is shown for many cells backed up by statistical tests. However, it is not explained in the text (not in methods) what is %clustering, how was that quantified.
We thank the referee for the comment. Please note that the requested information was presented in the original submission in the Methods section under "Microscopy image analysis" and described in detail in our previous publication (*Science* 379: 1149-1156), cited in the original submission and the current one.

Also, the authors refer to change in condensate size, which is not possible to see from these plots at all.
As suggested by the referee, the revised manuscript makes clear what is meant by condensate size.

I am not convinced that KE motif 3 regulates condensation (page 11). In fact, Fig 3G shows that it does not in vivo.
Contrary to what the referee states, the data in Fig 3F and Fig 3G establish that the charge of the KE motif 3 plays a role in *Bt*Rho condensation both *in vivo* and *in vitro*.

And even the authors say at the end of the section: RNA interactions with KE motif 1 abd KE motif 2 are the main drivers of BtRho condensation in vivo. So is there a consistent discrepancy between the in vivo and in vitro conclusions?
We thank the referee for the comment. Please note that there is no discrepancy between the *in vivo* and *in vitro* results because the phenotypes of the KE motif 1 and KE motif 2 mutants unequivocally demonstrate that the KE motif 1 and KE motif 2 are key to condensate formation by *Bt*Rho.

I am also puzzled by the shuffling variants increasing condensation. Wouldn't that mean that there is no sequence specificity required for the KE 1 and KE 2 motifs?
We thank the referee for the comment. Please note that there our results do establish that specific sequences in KE motif 1 and KE motif 2 are necessary for phase separation taking place in response to carbon starvation. This is because the regulated condensation of wild-type *Bt*Rho is lost when the sequences corresponding to KE motif 1 and KE motif 2 are substituted by other sequences. That is, *Bt*Rho condensation takes place to the same extent in the presence of glucose as under carbon starvation. (If the shuffled variants of KE motif 1 and KE motif 2 had behaved like wild-type *Bt*Rho, one would have concluded that the sequence specificity of the KE motifs 1 and 2 is not important.)

Why do the mutants loose the ability to respond to glucose in Figure 3?
We thank the referee for the comment. As stated above, the results in Figure 3 demonstrate that specific sequences in KE motif 1 and KE motif 2 are required for carbon starvation-induced condensate formation by *Bt*Rho.

Minor comments:
Abstract: I would recommend revising the first two sentences. The first one is too long and the second one is quite repetitive.
We thank the referee for the comment and appreciate the suggestion to modify the sentences. However, we believe that the sentences are clear as written. Moreover, we believe that what may feel repetitive to an experienced reader, such as the referee, may actually help a naïve reader understand a complex topic.

Introduction: the relationship between condensates and membraneless bodies is not defined.
Contrary to what the referee writes, our manuscript states that "membraneless bodies" are the outcome of "responsive condensation".

Please indicate in Figure 1 the residue numbers of the different regions and indicate whenever only a fragment is shown.
As suggested by the referee, the revised manuscript includes a revised Figure 1 (panels A, D and E) indicating the residue number for the start/finish of each subdomain.

The alignment is panel C is misleading that it starts with residue number 1.
We thank the referee for the comment. Please note that the figure indicates the actual amino acid number of the sequences shown right next to the species name. The numbering on top of the alignment is necessary to help the reader locate the sequences within the large IDR of *Bt*Rho.

A supplementary table with all fragments and mutants' regions and sequences would be helpful.

As suggested by the referee, the revised manuscript includes a new table with the amino acid sequences of the various *Bt*Rho variants discussed on our manuscript (Appendix Table S3).

Also, I would use a color that represents the amino acid type in panel C.
We thank the referee for the comment. However, please note that the goal of panel C is to highlight the amino acid sequence conservation (or lack thereof) between the IDR from *Bt*Rho and the IDR from the Rho protein from other *Bacteroides* species. Please note that the amino acid types are indicated by colors in panels D and E of the same figure.

Consider splitting the second chapter of the Results into two, since it is very long.
As suggested by the referee, the revised manuscript includes a new version of the second chapter of the Results section split into two: one discussing the results of the *in vitro* assays and one with the results of the *in vivo* assays.

Figure 5F. I am not sure what better, similar or worse than WT means here for each of the experiments. I would revise and make it clearer.
As suggested by the referee, the revised manuscript makes clear what "similar or worse than WT" means.

Having a summary figure is a good idea, but I would add it to Figure 7 instead since it is not only information about simulations, but almost all experiments. Actually, the pppGpp experiments could be added to it as well.
As suggested by the referee, the revised manuscript includes a revised Figure 7 that incorporates the role of ppGpp based on the results reported in the original submission and the new ones carried out since then.

The schematics in Figure 7. The pink colors look the same when printed. Consider a stronger color gradient to distinguish the KE motifs.
As suggested by the referee, the revised manuscript includes a revised Figure 7 using a different color gradient.

It is not intuitive what interactions would drive condensation in nutrient conditions since no inter-chain interactions are indicated? Could simulations help suggesting what would drive condensation?
As suggested by the referee, the revised manuscript includes a revised Figure 7 and modified text to indicate the interactions taking place under different nutritional conditions that impact *Bt*Rho condensate formation. This is in addition to a new figure (EV6) showing simulations of interchain interactions. Specifically, the new figure (EV6) reports the use of simulations to quantify the extent two *Bt*Rho IDR regions can interact intermolecularly. We find that the positive charged regions of KE motif 1 and KE motif 2 can make intermolecular interactions with the negatively charged regions of KE motif 3 and BCD.

I suggest to add a few sentences on the alarmon (p)ppGpp and its relevance to stress response and btRho activation as an introduction at the beginning of the section.

As suggested by the referee, the revised manuscript includes an introductory sentence describing (p)ppGpp's functions in bacteria, its relevance in the context of stress responses, and why it was relevant to investigate its role in condensate formation of *Bt*Rho in *Bacteroides thetaiotaomicron*.

DRaCALA experiments: why not showing all three replicates? The captions state, that three independent experiments were performed and a representative image is shown. There would be enough space to show all three in Figures 6CD and EV4.

As suggested by the referee, the revised manuscript includes the DRaCALA repeats in Appendix Figure S1.

Non-essential suggestions:

Would it be possible to perform simulations including RNA? The fact that the FINCHES predictions suggest that the positively charged motifs should bind RNA and the negatively charged ones agrees with purely electrostatic considerations. Would that look different for poly-rG vs. poly-rU? Since the alarmon sequence and GTP binding was shown to be important. Or there is no expectation on sequence-specificity?

We thank the referee for the comment. Unfortunately, it is not possible to include RNA in the simulations with currently available techniques. Please note that our previous publication on *Bt*Rho (*Science* 379: 1149-1156) indicated the existence of specificity in terms of the ability of an RNA to promote condensate formation by *Bt*Rho. A detailed analysis to define the specific RNA features that are necessary to promote condensate formation by *Bt*Rho is beyond the scope of this manuscript, which focuses on the role of specific domains and motifs on condensate formation.

Referee #2 (Report for Author)

In this manuscript, Krypotou and colleagues expand on their groundbreaking discovery that the transcription termination factor Rho from Bacteroides thetaiotaomicron (BtRho) forms biomolecular liquid-liquid phase-separated (LLPS) condensates, which are critical for the fitness of the bacterium in the mammalian gut (Science 2023, PMID: 36927025). This study presents the only well-characterized example of Rho activity -critical across many bacteria- being regulated through LLPS condensation, though a recent preprint suggests a potentially similar mechanism in mycobacterial Rho (PMID: 40909676), albeit with less comprehensive evidence.

Through a rigorous, multidisciplinary approach-combining phylogenetic analyses, molecular dynamics simulations, and extensive in vitro/in vivo characterization of BtRho domain mutants-the authors elucidate the molecular mechanisms underlying BtRho condensation. They identify conserved domains within an intrinsically disordered region (IDR) of BtRho that both mediate and regulate LLPS condensation in an RNA-

dependent manner.
Given that BtRho condensation is enhanced under carbon starvation in vivo (Science 2023), the authors further hypothesized that the alarmone ppGpp, which accumulates under such conditions, may also promote BtRho condensation. While they provide some indirect support for this idea, this remains the least substantiated (yet potentially most exciting) conclusion in an otherwise thorough and well-designed study. This hypothesis is particularly intriguing in light of recent findings in E. coli, where ppGpp inactivates Rho through a distinct 'hyper-oligomerization' mechanism (PMID: 39952913). If confirmed, the present work would not only generalize the role of ppGpp in regulating Rho activity but also demonstrate that such stress-related regulation can manifest through diverse molecular strategies.
We thank the referee for the positive comments. Below please find how we incorporated the useful suggestions of the referee.

Given the broad, potential impact of these findings, I recommend the authors address the following points in a revised manuscript:
Main Points:
1) ppGpp stimulation: The proposed role of ppGpp in BtRho condensation is based on two observations: first, carbon starvation (absence of glucose) fails to stimulate BtRho condensate formation in a ppGpp^0 strain; second, BtRho can bind ppGpp in vitro. However, direct evidence that ppGpp stimulates BtRho condensation is lacking. The in vivo effects could arise from any of the numerous metabolic alterations in the ppGpp^0 background, while ppGpp binding may not be specific -GTP, for example, effectively competes with ppGpp (Fig. EV4B) - nor responsible for condensate formation. Notably, the authors extensively characterized BtRho condensation in vitro using DIC microscopy, yet they did not test ppGpp's effect under these controlled conditions. A direct demonstration of ppGpp's stimulatory effect on BtRho condensation (with or without RNA) would significantly strengthen their conclusion, linking a critical bacterial alarmone to a novel regulatory mechanism.
As suggested by the referee, the revised manuscript includes new data showing how ppGpp impacts condensation formation by *Bt*Rho *in vitro*, and how this process is influenced by RNA and the divalent cation Mg^{2+}.

2) Additional controls, such as testing other nucleotides (particularly ATP) via DIC microscopy and DRaCALA blotting, or the effect of ppGpp on Rho-dependent transcription termination (e.g., using the in vitro assay from their original work: PMID: 36927025) would further clarify the specificity and functional relevance of ppGpp binding to BtRho's IDR.
We thank the referee for the comment. Our manuscript demonstrates the specificity of *Bt*Rho by showing that it binds to ppGpp but not to pppGpp, arguably the closest nucleotide to ppGpp; and that the binding is dependent on the IDR of *Bt*Rho. Investigating *Bt*Rho binding to additional nucleotides is beyond the scope of this manuscript. For example, the results of experiments exploring ATP binding to full-length *Bt*Rho would be hard to interpret given that *Bt*Rho binds ATP *independently* of its IDR, which is in contrast to ppGpp, as established in our original submission.

3) Role of Rho Oligomerization: What are the contributions of Rho's C-ter domain and Rho oligomerization (e.g., hexamer formation) to condensate formation? Can the IDR alone (or with RNA) form condensates or is the high local concentration/proximity of IDRs in Rho hexamers critical for LLPS condensation?

We thank the referee for the comment. Our Science paper on condensate formation by *Bt*Rho established that the IDR alone (thus, lacking the sequences in the conserved portion of *Bt*Rho involved in oligomerization) can form condensates *in vivo* and *in vitro* (*Science* 379: 1149-1156). However, when the *Bt*Rho IDR is expressed by itself, it loses the regulation by carbon starvation *in vivo*, suggesting that being within the full *Bt*Rho protein matters. Our manuscript focuses on the role played by specific sequences and domains within the IDR on condensate formation by *Bt*Rho. Exploring the role of Rho oligomerization is beyond the scope of this manuscript.

Minor points:

4) Phenotypic Heterogeneity: The extensive in vivo work-both here and in the previous study-reveals significant heterogeneity among Bacteroides cells under any given condition. How were cells selected for imaging?

We thank the referee for the comment. Our revised manuscript makes clear that cells that were fully stained were selected, with >50 cells being analyzed per experiment.

Is this heterogeneity linked to experimental limitations, or does it reflect genuine phenotypic diversity?

We thank the referee for the comment. The heterogeneity could be due to both technical limitations of staining and phenotypic diversity. Please note that bacteria expressing the condensate-defective ΔIDR and ΔKErich variants of *Bt*Rho, which are missing critical sequences required for condensate formation, display less variation than bacteria expressing the wild-type *Bt*Rho.

If the latter, how do the authors interpret this variability? Could it provide some advantage at the population level?

The referee raises the intriguing possibility of heterogeneity in *Bt*Rho condensate formation potentially providing an advantage at the population level. We are interested in this possibility and the results of these investigations will be reported elsewhere.

5) Figure 2C: The figure legend mentions error bars representing SEM, but none are visible in Figure 2C. This discrepancy should be clarified.

Contrary to what the referee writes, error bars are present in Figure 2C.

Referee #3 (Report for Author)

The Groisman group has previously shown that the transcription termination factor Rho from Bacteroides thetaiotaomicron (BtRho) phase separates in response to carbon starvation, and that the 303-residue IDR at its N-terminus, which is absent in Rho from most studied model bacteria, is necessary for condensation both in

vivo and in vitro. The group has further shown that phase separation of BtRho increases its transcription termination activity and promotes fitness of Bt in the mammalian gut.
In the current manuscript, the Groisman group and collaborators set out to reveal the specific protein sequences and the small molecules that control BtRho condensation. They identified short, charged or conserved motifs within the IDR that govern BtRho condensation, established specific interactions that these motifs mediate, both within the IDR itself and with RNA, suggest that the alarmone ppGpp is essential for the carbon starvation-induced condensation of BtRho, and showed that ppGpp has the capacity to bind directly to BtRho in a manner that depends on the IDR.
The manuscript reports novel and original findings, is well written and should be suitable for publication in EMBO J after addressing the following points below. Since the manuscript contains a substantial body of work, in addition to suggesting experimental approaches to address the different points, I leave it up to the authors to state the limitations in the text, instead of addressing them experimentally.
We thank the referee for the positive comments.

Major comments:
1. Revealing specific protein sequences that control condensation is of importance, since they may provide insights on the mechanism that drives phase separation in general. However, the authors declare that they revealed also a molecule that control the process and are necessary for it, that is, ppGpp. In my opinion, the latter finding is the "cherry" that enhances the attraction of this manuscript. Therefore, I find the lack of in vitro studies to characterize the involvement of ppGpp in condensation as a drawback. This lack stands out, since the balance between the in vivo and in vitro results is one of the strengths of the manuscript, and the exception is the claim regarding the essentiality of ppGpp for condensation.
As suggested by the referee, the revised manuscript includes new data showing how ppGpp impacts condensation formation by *Bt*Rho *in vitro*, and how this process is influenced by RNA and the divalent cation Mg^{2+}.

Indeed, binding assays of ppGpp to BtRho and its derivatives (Figure 6C) indirectly supports the notion that the alarmone is involved in condensation. However, whereas RNA is shown to drive BtRho condensation in vitro without ppGpp (Figures 2B, 3D, 3F, 4C), ppGpp is shown to be absolutely required for condensation in vivo (Figure 6A). A discrepancy between in vitro and in vivo observations regarding ppGpp effect was documented (e.g., Carmona et al., J. Bact. 2000), suggesting that the cellular environment, other regulatory networks and the cytoplasmic crowding influences regulation by ppGpp in ways that may not be fully mimiced by in vitro experiments (the latter is supported by the enhancement of BtRho condensation by the crowding agent dextran). Yet, in other studies, in vitro assessment of ppGpp effect supported the in vivo results, although not exactly mirroring them (e.g., Vrentas et al, Genes Dev. 2005), suggesting that such studies are worth trying.
As suggested by the referee, the revised manuscript includes new data showing how ppGpp impacts condensation formation by *Bt*Rho *in vitro*, and how this process is influenced by RNA and the divalent cation Mg^{2+}. The new *in vitro* results establish that ppGpp *directly* impacts condensate

formation by *Bt*Rho. Of course, ppGpp and/or pppGpp might play additional roles in the *in vivo* formation of *Bt*Rho condensates via RNAs, proteins, and/or metabolites the abundance and/or activity of which is controlled by ppGpp and/or pppGpp.

This gap in the manuscript can be addressed by the authors experimentally either by measuring the binding affinity between BtRho and an RNA ligand in the absence and presence of ppGpp by a quantitative method, such as ITC or MST. This will provide a direct mechanistic link between RNA and ppGpp and resolve the apparent contradiction between the in vitro and in vivo findings.
We thank the referee for the comment. As stated above, the revised manuscript includes new data showing how ppGpp *directly* impacts condensation formation by *Bt*Rho *in vitro*, and how this process is influenced by RNA and the divalent cation Mg^{2+}. We believe that further exploration of RNA binding to *Bt*Rho is beyond the scope of this manuscript.

Alternatively, the authors can perform in vitro condensation assays with BtRho WT and variants lacking the KE or PRD domains in the presence of physiological concentrations of ppGpp.
As suggested by the referee, the revised manuscript includes new data showing how ppGpp impacts condensation formation by *Bt*Rho *in vitro*, and how this process is influenced by RNA and the divalent cation Mg^{2+}. Please note that *Bt*Rho derivates lacking the KE region do not form condensates and those lacking the PLD form condensates even in the absence of the carbon starvation signal.

This approach will directly test if ppGpp is required for or enhances RNA-driven condensation also in vitro. If the authors decide not to resolve the discrepancy experimentally, they should clearly acknowledge this limitation in the Discussion. If the authors attempt/ed to test the effect of ppGpp in vitro and could not reproduce the in vivo results, reporting this unsuccessful attempt is important for other investigators.
As suggested by the referee, the revised manuscript includes new data showing how ppGpp impacts condensation formation by *Bt*Rho *in vitro*, and how this process is influenced by RNA and the divalent cation Mg^{2+}. The new *in vitro* results establish that ppGpp directly impacts condensate formation by *Bt*Rho. Of course, ppGpp and/or pppGpp might play additional roles in the *in vivo* formation of *Bt*Rho condensates via RNAs, proteins, and/or metabolites the abundance and/or activity of which is controlled by ppGpp and/or pppGpp.

Additionally, since the authors suggest a novel role for ppGpp, they should mention that the molecular mechanism by which ppGpp binding leads to condensation remains to be elucidated.
As suggested by the referee, the revised manuscript includes new data showing the impact that ppGpp has on condensate formation by BtRho, and how it is influenced by RNA and the divalent cation Mg^{2+}. The biological meansing of the results of these experiments are discussed in the Discussion section of the revised manuscript.

2. The authors focus on very specific protein motifs that mediate RNA-driven phase separation. In sharp

contrast, they used total RNA extract from Bt for the in vitro experiments (Figures 2B, 3D, 3F, 4C). The problem of using total RNA mix is that rRNAs and tRNAs are the most abundant transcripts and, hence, may take over and lead to conclusions that are not physiologically relevant.
We thank the referee for the comment. Our manuscript focuses on the role played by specific sequences and domains within the IDR in on condensate formation by BtRho. Investigating the RNA specificity is of interest and will be addressed in a future publication as it is beyond the scope of this manuscript.

Also the use of total RNA extract precludes the possibility to draw conclusions about the interactions between BtRho motifs and RNA at a sequence level. In light of studies that highlighted the importance of specific motifs in both proteins and RNAs for co-phase separation, I suggest that the authors perform control in vitro condensation assays using defined ligands, such as poly A or poly U, or, better, the typical BtRho binding sites, assuming that some are known or can be identified by a relatively simple assay. Again, the least that the authors should do is to acknowledge this limitation by stating that their RNA mixture is dominated by interactions with abundant rRNA species.
As suggested by the referee, we established that polyA promotes condensate formation of *Bt*Rho. Investigating the RNA specificity is of interest and will be addressed in a future publication as it is beyond the scope of this manuscript.

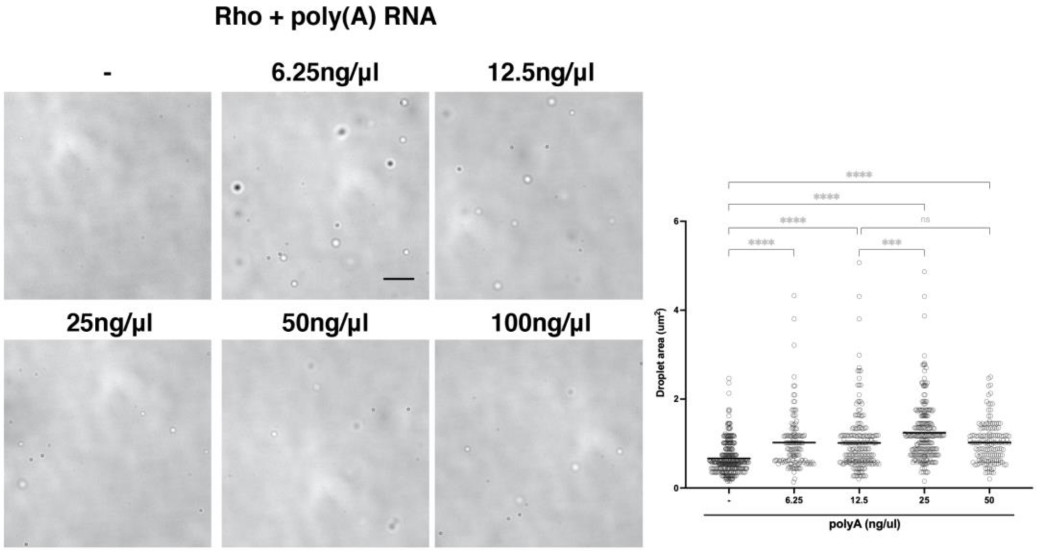

Figure 1. The effect of polyA on BtRho condensation in vitro.

Minor comments:
1. The authors do not provide direct evidence that the KE-rich and PLD subdomains alone are sufficient for condensation. Reconstituting the system with the synthesized subdomains or with minimal components (subdomains, synthetic RNA, potential cofactors) will address this limitation. Addressing this limitation in the

text will require replacing "sufficient for condensation" with "play critical role in (or are important for) condensation".
As suggested by the referee, the revised manuscript has a modified text to indicate that the identified motifs “play a critical role in condensation” instead of being “sufficient for condensation”. Synthesizing artificial IDRs with minimal domains and testing the effects of specific RNAs is beyond the scope of the present manuscript.

2. Still about the in vitro system: The authors should explain why they chose certain protein concentrations and buffer conditions and how they relate to the cellular environment.
As suggested by the referee, the revised manuscript makes clear that we used physiological concentrations of *Bt*Rho and salts.

My suggestion is that the authors also perform in vitro condensation assays with cell lysates to better imitate physiological conditions. This is relevant also for Major Point 1 (re ppGpp).
We thank the referee for the comment. However, please note that whatever the outcome of the proposed experiment will not change the conclusions obtained with the results already available. Moreover, the additional results obtained since the original submission provide further support to the conclusions of the manuscript.

3. Temporal information is missing: Figure 2C shows the results 30 minutes post-starvation, neglecting possible differences in assembly or disassembly kinetics between the variants. It would be good to add a time-course analysis of condensate formation by fixing samples at various timepoints.
As suggested by the referee, we determined that at 15 minutes there is less condensation that at 30 minutes. A detailed time course of this process is beyond the scope of this manuscript.

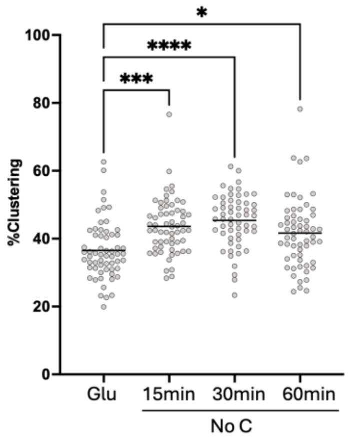

Figure 2. Wild type BtRho clustering.

4. While the data in Figures 3D and 3E strongly suggest that the two KE motifs mediate RNA-dependent condensation, evidence for direct interaction with the RNA, such as EMSA, is lacking. A test that compares WT to variants with the KE charge neutralized would provide evidence for direct binding of the KE motifs to RNA. Otherwise, acknowledging in the text that direct binding remains to be biochemically confirmed is needed.

We thank the referee for the comment. As stated in our manuscript, RNA elicits condensate formation of *Bt*Rho in a manner dependent on the KE-rich region *in vitro*. Given that *Bt*Rho protein and RNA were the only components in the assay, the simplest interpretation of these results is the RNA elicits *Bt*Rho condensation directly. These data are supported by our *Science* paper demonstrating colocalization of RNA with *Bt*Rho droplets (*Science* 379: 1149-1156). Furthermore, the revised manuscript includes new data showing how ppGpp impacts condensation formation by *Bt*Rho *in vitro*, and how this process is influenced by RNA and the divalent cation Mg^{2+}. We believe that an in-depth exploration of the types of RNAs that elicit *Bt*Rho condensation is beyond the scope of this manuscript.

I hope that you will find the revised manuscript suitable for publication in *The EMBO Journal*.

With best wishes,

Eduardo A. Groisman, Ph.D.
Waldemar Von Zedtwitz Professor of Microbial Pathogenesis

1st Revision - Editorial Decision **17th Mar 2026**

Dear Prof. Groisman,

Thank you for submitting a revised version of your manuscript. Your study has now been seen by all original referees, who find that their previous concerns have been mostly addressed and only request minor revisions which I would ask you to incorporate into the final version of the manuscript. In addition there are a few mainly editorial points that have to be addressed before I can extend formal acceptance of the manuscript:

- As we are switching from a free-text author contribution statement towards a more formal statement based on Contributor Role Taxonomy (CRediT) terms, please remove the present Author Contribution section and instead specify each author's contribution(s) directly in the Author Information page of our submission system during upload of the final manuscript. See https://casrai.org/credit/ for more information.

- APPENDIX FILE WITH ToC: Table of Contents on the title page needs to have each item listed (figures, tables) with its page number

- Please provide suggestions for a short 'blurb' text prefacing and summing up the conceptual aspect of the study in two sentences (max. 250 characters), followed by 3-5 one-sentence 'bullet points' with brief factual statements of key results of the paper; they will form the basis of an editor-written 'Synopsis' accompanying the online version of the article. Please also provide an altered synopsis image, making sure that the aspect ratio conforms to our website's format - it should be exactly 550 pixels wide and between 300-600 pixels high.

- The manuscript sections should be in the following order: Title page - Abstract & Keywords - Introduction - Results - Discussion - Methods - Data Availability - Acknowledgments - Disclosure and Competing Interests Statement - References - Figure Legends - (Main Tables with legends if applicable) - Expanded View Figure Legends.

- Please note that the legends for figures 3 is not provided in the sequential manner (legend for figure F is provided before legend of figure E).

- Please note that the exact p values are not provided in the legends of figures 2C, 3E, G; 4D, 6A, B

Please let me know if you have any questions regarding any of these points. You can use the link below to upload the revised files.
Thank you again for giving us the chance to consider your manuscript for The EMBO Journal. I look forward to receiving the final version.

With best regards,

Cornelius Schneider

____

Cornelius Schneider, PhD
Editor | The EMBO Journal
c.schneider@embojournal.org

Please refer to our figure preparation guideline in order to ensure proper formatting and readability in print as well as on screen:

https://link.springer.com/journal/44318/submission-guidelines#cms-Figure-and-data-presentation

Use the link below to submit your revision:

https://emboj.msubmit.net/cgi-bin/main.plex

Referee #1:

Referee #2:

Krypotou and colleagues have substantially revised and improved their manuscript, effectively addressing most of my initial concerns. While they now provide in vitro evidence demonstrating the (modest) effect of ppGpp on BtRho condensation, they do not directly show that ppGpp enhances BtRho's transcription termination activity. As such, it remains possible that ppGpp-induced BtRho condensates possess distinct, moonlighting functions-highlighting the important distinction between correlation and causality.
That said, not every question can be resolved within a single study. The depth of work and the novel insights presented in this revised manuscript are, in my view, more than sufficient to justify publication in the EMBO Journal.

Referee #3:

As I commented on the original manuscript, before revision, the manuscript reports novel and original findings and, in principle, should be suitable for publication in EMBO J after revision.

I made only two major comments: 1. The need to characterize the involvement of ppGpp in BtRho condensation, and 2. The problem in using total RNA extract from Bt, composed mainly of rRNAs and tRNAs, for the in vitro experiments, rather than more defined RNAs.

I response to point 1, the authors included new data, which show how ppGpp impacts condensation by BtRho in vitro, and the effect of RNA and magnesium on this process. Although I suggested a more quantitative approach than the authors took, I think that the new data can be accepted as sufficient.

The response of the authors to point 2 is that "Investigating the RNA specificity is of interest and will be addressed in a future publication as it is beyond the scope of this manuscript".
In light of the reports that co-phase separation depends on specific motifs in both proteins and RNAs, I regard basic characterization of the RNA as complementary to protein characterization.
Moreover, the authors included two figures in the rebuttal letters, which are not included in the manuscript. According to the rebuttal letter, in Fig. 1 they "established that polyA promotes condensate formation of BtRho". I am not surprised that this figure is not included in the revised manuscript, as it does not provide the proof for their claim.
Hence, I leave it up to the editor to decide whether point 2 can be waived.

Yale SCHOOL OF MEDICINE

Department of Microbial Pathogenesis

Boyer Center for Molecular Medicine

295 Congress Ave, Room 354D
New Haven CT 06519
203-737-7940

January 23, 2025

Cornelius Schneider, PhD
Editor
The EMBO Journal

Re: EMBOJ-2025-122310R

Dear Cornelius,

Attached please find the final version of our revised manuscript titled "Short autoinhibitory sequences control phase separation of essential bacterial transcription factor" in which we incorporated the requests made by you on your message of March 17, 2026.

Dear Prof. Groisman,

Thank you for submitting a revised version of your manuscript. Your study has now been seen by all original referees, who find that their previous concerns have been mostly addressed and only request minor revisions which I would ask you to incorporate into the final version of the manuscript. In addition there are a few mainly editorial points that have to be addressed before I can extend formal acceptance of the manuscript:

- As we are switching from a free-text author contribution statement towards a more formal statement based on Contributor Role Taxonomy (CRediT) terms, please remove the present Author Contribution section and instead specify each author's contribution(s) directly in the Author Information page of our submission system during upload of the final manuscript. See https://casrai.org/credit/ for more information.

As requested, the re-revised manuscript no longer includes the Contribution section.

- APPENDIX FILE WITH ToC: Table of Contents on the title page needs to have each item listed (figures, tables) with its page number

As requested, the re-revised manuscript includes the missing information on the ToC page in the Appendix.

- Please provide suggestions for a short 'blurb' text prefacing and summing up the conceptual aspect of the study in two sentences (max. 250 characters), followed by 3-5 one-sentence 'bullet points' with brief factual statements of key results of the paper; they will form the basis of an editor-written 'Synopsis' accompanying the online version of the article. Please also provide an altered synopsis image, making sure that the aspect ratio conforms to our website's format - it should be exactly 550 pixels wide and between 300-600 pixels high.

As requested, the resubmission includes a blurb, bullet points, and synopsis image.

- The manuscript sections should be in the following order: Title page - Abstract & Keywords - Introduction - Results - Discussion - Methods - Data Availability - Acknowledgments - Disclosure and Competing Interests Statement - References - Figure Legends - (Main Tables with legends if applicable) - Expanded View Figure Legends.

As requested, the re-revised manuscript was verified to have the correct order of the sections.

- Please note that the legends for figures 3 is not provided in the sequential manner (legend for figure F is provided before legend of figure E).

As requested, the re-revised manuscript has a revised figure legend with the correct sequential order.

- Please note that the exact p values are not provided in the legends of figures 2C, 3E, G; 4D, 6A, B

We thank the Editor for the comment. Please note that figures 2C, 3E, 3G, and 4D show the *p* values for $p<0.05$ for the indicated comparisons in the figure itself. This is why they are not shown again in the figure legend. We believe that also including *p* values >0.05 would hinder interpretation of the figure. Please note that in figures 6A, 6B, all the *p* values for the indicated comparisons are shown.

Referee #1

Thank you, this makes the data representation and the conclusions a lot clearer. However, it would be necessary to add effect sizes/p-values to the statements when comparisons are made, particularly that refer to Figure 2 and 3 and Figure EV 1-7. I do not want to list all statements that would need to be backed up by statistical analysis. I just give one example where the figure itself does not help to support the claim. For instance, the statement below needs a quantitative statement and p-value (show the median is different and the difference is significant):
"Variants lacking KE motif 1 or KE motif 2 formed condensates that were larger in size when compared to those formed by wild-type BtRho (Figs. 3D and EV4A)."

As suggested by the reviewer, the re-revised manuscript now includes the median value for each condition in all the graphs showing condensate quantification. Please note that the *in vitro* condensation assays provide multiple information including number of droplets, size of droplets, total surface area of all the droplets, and biophysical properties of the droplets. Therefore, drawing

conclusions based on a single property could lead to wrong conclusions. This is the reason why statistical analysis between median values is not provided as it could be confusing to the reader.

I am sorry for overlooking this. Perhaps it is worth to add a sentence about this metric in the results section, since this is not a standardly used measure of condensate formation?

As suggested by the reviewer, the re-revised manuscript now includes a sentence about the quantification method in the Results section.

All the above discussion and disagreement may stem from the fact what we mean by a motif, is it the exact sequence? That would be my definition. If a motif is important for condensation, then shuffling it should reduce %clustering. The fact that clustering actually increases condensation, shows that the exact motif is not important. Together with the fact that the neutral version of the motif has lower %clustering, one can conclude that only the charges matter. Thus, condensation is not dependent on the exact sequence motif, only on charge composition. On the other hand, the condensation becomes independent of glucose. So glucose-dependent condensation does depend on the sequence.
Please clarify these statements in the text and also in Figure 7 which states that the KE motifs are essential for condensation and change to essential for glucose-dependent condensation or glucose-response etc.

We thank the reviewer for the comment. As discussed in our manuscript, condensation depends on a complex network of interactions including intra-domain, inter-domain, heterotypic, and homotypic by the IDR. That sequence shuffling of a conserved motif within the IDR increases condensation actually argues that the shuffled variant has lost the ability to suppress interactions that the wild-type *Bt*Rho protein normally performs. This result does establish that the specific sequence of the motif (not just its amino acid composition) is necessary for wild-type *Bt*Rho condensation. Finally, as correctly summarized in Figure 7, KE motifs 1 and 2 are essential for *Bt*Rho condensation. This is because *Bt*Rho variants lacking KE motifs 1 and 2 fail to form condensates *in vivo* and *in vitro*.

I believe that many synonyms and unclear terms are used across publications. I am not familiar with the term 'responsive condensation' = membraneless bodies. Are membraneless bodies a subset of membraneless organelles, which is a frequently used term in the literature? Are membraneless organelles a subset or equivalent to biomolecular condensates? Please define the usage of these terms here because they are not yet fixed definitions by the community.

We thank the referee for the comment. To avoid confusion, we changed the "membraneless bodies" to "biomolecular condensates" in the re-revised manuscript.

I could not find this information in the caption: (F) Summary of the relative change for the type of interactions that modulate condensation for all variants examined both experimentally and computationally compared to wild-type BtRho.

We thank the reviewer for the comment. Please note that a detailed description of each of the comparisons is provided in the main text of the manuscript. To avoid unnecessary redundancy, the manuscript does not repeat the same information in the figure caption.

Referee #2

Krypotou and colleagues have substantially revised and improved their manuscript, effectively addressing most of my initial concerns. While they now provide in vitro evidence demonstrating the (modest) effect of ppGpp on BtRho condensation, they do not directly show that ppGpp enhances BtRho's transcription termination activity. As such, it remains possible that ppGpp-induced BtRho condensates possess distinct, moonlighting functions-highlighting the important distinction between correlation and causality.
That said, not every question can be resolved within a single study. The depth of work and the novel insights presented in this revised manuscript are, in my view, more than sufficient to justify publication in the EMBO Journal.

We thank the reviewer for the comment. We agree that further studies are required to investigate whether stimulation of *Bt*Rho condensation by ppGpp also enhances *Bt*Rho's transcription termination activity. This is an area of interest that will be addressed in a future publication(s).

Referee #3

As I commented on the original manuscript, before revision, the manuscript reports novel and original findings and, in principle, should be suitable for publication in EMBO J after revision.
I made only two major comments: 1. The need to characterize the involvement of ppGpp in BtRho condensation, and 2. The problem in using total RNA extract from Bt, composed mainly of rRNAs and tRNAs, for the in vitro experiments, rather than more defined RNAs.
I response to point 1, the authors included new data, which show how ppGpp impacts condensation by BtRho in vitro, and the effect of RNA and magnesium on this process. Although I suggested a more quantitative approach than the authors took, I think that the new data can be accepted as sufficient.
The response of the authors to point 2 is that "Investigating the RNA specificity is of interest and will be addressed in a future publication as it is beyond the scope of this manuscript".
In light of the reports that co-phase separation depends on specific motifs in both proteins and RNAs, I regard basic characterization of the RNA as complementary to protein characterization.
Moreover, the authors included two figures in the rebuttal letters, which are not included in the manuscript. According to the rebuttal letter, in Fig. 1 they "established that polyA promotes condensate formation of BtRho". I am not surprised that this figure is not included in the revised manuscript, as it does not provide the proof for their claim.
Hence, I leave it up to the editor to decide whether point 2 can be waived.

We thank the reviewer for the comment. We are also interested in defining the effect that specific RNAs have on *Bt*Rho condensation. Our Science 2023 paper reported the identification of two RNAs that differ in their ability to elicit *Bt*Rho phase separation despite *Bt*Rho acting on both RNAs to

terminate transcription. Our current manuscript dissects the distinct roles of different regions of the complex BtRho IDR, as well as specific motifs within these regions, play on *Bt*Rho phase separation. Moreover, the manuscript reveals a role for the alarmone ppGpp in *Bt*Rho condensation *in vivo* and *in vitro*. We believe that an investigation of the effect and specificity that additional RNAs have on *Bt*Rho condensation is beyond the scope of this manuscript.

I hope that you will find the revised manuscript suitable for publication in *The EMBO Journal*.

With best wishes,

Eduardo A. Groisman, Ph.D.
Waldemar Von Zedtwitz Professor of Microbial Pathogenesis

2nd Revision - Editorial Decision 8th Apr 2026

Dear Prof. Groisman,

I am pleased to inform you that your manuscript has been accepted for publication in the EMBO Journal.

Your manuscript will be processed for publication by EMBO Press. It will be copy edited and you will receive page proofs prior to publication. Please note that you will be contacted by Springer Nature Author Services to complete licensing and payment information.

You may qualify for financial assistance for your publication charges - either via a Springer Nature fully open access agreement or an EMBO initiative. Check your eligibility: https://link.springer.com/journal/44318/how-to-publish-with-us

Should you be planning a Press Release on your article, please get in contact with embo_production@springernature.com as early as possible in order to coordinate publication and release dates.

If you have any questions, please do not hesitate to contact the Editorial Office. Thank you for your contribution to The EMBO Journal.

Yours sincerely,

Cornelius Schneider, PhD
Editor
The EMBO Journal
c.schneider@embojournal.org

*****
Please note that it is The EMBO Journal policy for the transcript of the editorial process (containing referee reports and your response letters) to be published as an online supplement to each paper. If you should prefer removal of any referee-only figures included in the point-by-point response(s), e.g. because they may still be used for future publication or because they have been reproduced from published work by others, please do let us know immediately via response email.
More information is available here: https://link.springer.com/partners/embo-press/editorial-policies#Peer%20review

** Click here to be directed to your login page: https://emboj.msubmit.net

EMBO Press Author Checklist

Corresponding Author Name: Eduardo A. Groisman, Rohit V. Pappu
Journal Submitted to: The EMBO Journal
Manuscript Number: EMBOJ-2025-122310

USEFUL LINKS FOR COMPLETING THIS FORM
The EMBO Journal - Author Guidelines
EMBO Reports - Author Guidelines
Molecular Systems Biology - Author Guidelines
EMBO Molecular Medicine - Author Guidelines

Reporting Checklist for Life Science Articles (updated January
This checklist is adapted from Materials Design Analysis Reporting (MDAR) Checklist for Authors. MDAR establishes a minimum set of requirements in transparent reporting in the life sciences (see Statement of Task: 10.31222/osf.io/9sm4x). Please follow the journal's guidelines in preparing your
Please note that a copy of this checklist will be published alongside your article.

Abridged guidelines for figures
1. Data
The data shown in figures should satisfy the following conditions:
- the data were obtained and processed according to the field's best practice and are presented to reflect the results of the experiments in an accurate and unbiased manner.
- ideally, figure panels should include only measurements that are directly comparable to each other and obtained with the same assay.
- plots include clearly labeled error bars for independent experiments and sample sizes. Unless justified, error bars should not be shown for technical
- if n<5, the individual data points from each experiment should be plotted. Any statistical test employed should be justified.
- Source Data should be included to report the data underlying figures according to the guidelines set out in the authorship guidelines on Data

2. Captions
Each figure caption should contain the following information, for each panel where they are relevant:
- a specification of the experimental system investigated (eg cell line, species name).
- the assay(s) and method(s) used to carry out the reported observations and measurements.
- an explicit mention of the biological and chemical entity(ies) that are being measured.
- an explicit mention of the biological and chemical entity(ies) that are altered/varied/perturbed in a controlled manner.
- the exact sample size (n) for each experimental group/condition, given as a number, not a range;
- a description of the sample collection allowing the reader to understand whether the samples represent technical or biological replicates (including how many animals, litters, cultures, etc.).
- a statement of how many times the experiment shown was independently replicated in the laboratory.
- definitions of statistical methods and measures:
  - common tests, such as t-test (please specify whether paired vs. unpaired), simple $\chi 2$ tests, Wilcoxon and Mann-Whitney tests, can be unambiguously identified by name only, but more complex techniques should be described in the methods section;
  - are tests one-sided or two-sided?
  - are there adjustments for multiple comparisons?
  - exact statistical test results, e.g., P values = x but not P values < x;
  - definition of 'center values' as median or average;
  - definition of error bars as s.d. or s.e.m.

Please complete ALL of the questions below.
Select "Not Applicable" only when the requested information is not relevant for your study.

Materials

Newly Created Materials	Information included in the manuscript?	In which section is the information available? (Reagents and Tools Table, Materials and Methods, Figures, Data Availability Section)
New materials and reagents need to be available; do any restrictions apply?	Yes	Data Availability Section

Antibodies	Information included in the manuscript?	In which section is the information available? (Reagents and Tools Table, Materials and Methods, Figures, Data Availability Section)
For **antibodies** provide the following information:<br>- Commercial antibodies: RRID (if possible) or supplier name, catalogue number and or/clone number<br>- Non-commercial: RRID or citation	Yes	Reagens and Tools Table

DNA and RNA sequences	Information included in the manuscript?	In which section is the information available? (Reagents and Tools Table, Materials and Methods, Figures, Data Availability Section)
**Short novel DNA or RNA including primers, probes**: provide the sequences.	Yes	Table EV2

Cell materials	Information included in the manuscript?	In which section is the information available? (Reagents and Tools Table, Materials and Methods, Figures, Data Availability Section)
**Cell lines:** Provide species information, strain. Provide accession number in repository **OR** supplier name, catalog number, clone number, and/**OR** RRID.	Not Applicable	
**Primary cultures:** Provide species, strain, sex of origin, genetic modification status.	Not Applicable	
Report if the cell lines were recently **authenticated** (e.g., by STR profiling) and tested for mycoplasma contamination.	Not Applicable	

Experimental animals	Information included in the manuscript?	In which section is the information available? (Reagents and Tools Table, Materials and Methods, Figures, Data Availability Section)
**Laboratory animals or Model organisms:** Provide species, strain, sex, age, genetic modification status. Provide accession number in repository **OR** supplier name, catalog number, clone number, **OR** RRID.	Not Applicable	
**Animal observed in or captured from the field:** Provide species, sex, and age where possible.	Not Applicable	
Please detail **housing and husbandry conditions**.	Not Applicable	

Plants and microbes	Information included in the manuscript?	In which section is the information available? (Reagents and Tools Table, Materials and Methods, Figures, Data Availability Section)
**Plants:** provide species and strain, ecotype and cultivar where relevant, unique accession number if available, and source (including location for collected wild specimens).	Not Applicable	
**Microbes:** provide species and strain, unique accession number if available, and source.	Yes	Table EV1

Human research participants	Information included in the manuscript?	In which section is the information available? (Reagents and Tools Table, Materials and Methods, Figures, Data Availability Section)
If collected and within the bounds of privacy constraints report on age, sex and gender or ethnicity for all study participants.	Not Applicable	

Core facilities	Information included in the manuscript?	In which section is the information available? (Reagents and Tools Table, Materials and Methods, Figures, Data Availability Section)
If your work benefited from core facilities, was their service mentioned in the acknowledgments section?	Yes	Acknowledgements

Design

Study protocol	Information included in the manuscript?	In which section is the information available? (Reagents and Tools Table, Materials and Methods, Figures, Data Availability Section)
If study protocol has been **pre-registered, provide DOI in the manuscript**. For clinical trials, provide the trial registration number **OR** cite DOI.	Not Applicable	
Report the **clinical trial registration number** (at ClinicalTrials.gov or equivalent), where applicable.	Not Applicable	

Laboratory protocol	Information included in the manuscript?	In which section is the information available? (Reagents and Tools Table, Materials and Methods, Figures, Data Availability Section)
Provide DOI OR other citation details if **external detailed step-by-step protocols** are available.	Not Applicable	

Experimental study design and statistics	Information included in the manuscript?	In which section is the information available? (Reagents and Tools Table, Materials and Methods, Figures, Data Availability Section)
Include a statement about **sample size** estimate even if no statistical methods were used.	Yes	Figure legends and Materials and Methods
Were any steps taken to minimize the effects of subjective bias when allocating animals/samples to treatment (e.g. **randomization procedure**)? If yes, have they been described?	Not Applicable	
Include a statement about **blinding** even if no blinding was done.	Not Applicable	
Describe **inclusion/exclusion criteria** if samples or animals were excluded from the analysis. Were the criteria pre-established? If sample or data points were omitted from analysis, report if this was due to attrition or intentional exclusion and provide justification.	Not Applicable	
For every figure, are **statistical tests** justified as appropriate? Do the data meet the assumptions of the tests (e.g., normal distribution)? Describe any methods used to assess it. Is there an estimate of variation within each group of data? Is the variance similar between the groups that are being statistically compared?	Yes	Figure legends and Materials and Methods

Sample definition and in-laboratory replication	Information included in the manuscript?	In which section is the information available? (Reagents and Tools Table, Materials and Methods, Figures, Data Availability Section)
In the figure legends: state number of times the experiment was **replicated** in laboratory.	Yes	Figure legends
In the figure legends: define whether data describe **technical or biological replicates**.	Yes	Figure legends

Ethics

Ethics	Information included in the manuscript?	In which section is the information available? (Reagents and Tools Table, Materials and Methods, Figures, Data Availability Section)
Studies involving **human participants**: State details of **authority granting ethics approval** (IRB or equivalent committee(s), provide reference number for approval.	Not Applicable	
Studies involving **human participants**: Include a statement confirming that **informed consent** was obtained from all subjects and that the experiments conformed to the principles set out in the WMA Declaration of Helsinki and the Department of Health and Human Services Belmont Report.	Not Applicable	
Studies involving **human participants:** For publication of **patient photos**, include a statement confirming that consent to publish was obtained.	Not Applicable	
Studies involving experimental **animals**: State details of **authority granting ethics approval** (IRB or equivalent committee(s), provide reference number for approval. Include a statement of compliance with ethical regulations.	Not Applicable	
Studies involving **specimen and field samples:** State if relevant **permits** obtained, provide details of authority approving study; if none were required, explain why.	Not Applicable	

Dual Use Research of Concern (DURC)	Information included in the manuscript?	In which section is the information available? (Reagents and Tools Table, Materials and Methods, Figures, Data Availability Section)
Could your study fall under dual use research restrictions? Please check biosecurity documents and list of **select agents and toxins** (CDC): https://www.selectagents.gov/sat/list.htm	Not Applicable	
If you used a select agent, is the security level of the lab appropriate and reported in the manuscript?	Not Applicable	
If a study is subject to dual use research of concern regulations, is the name of the **authority granting approval and reference number** for the regulatory approval provided in the manuscript?	Not Applicable	

Reporting

The MDAR framework recommends adoption of discipline-specific guidelines, established and endorsed through community initiatives. Journals have their own policy about requiring specific guidelines and recommendations to complement MDAR.

Adherence to community standards	Information included in the manuscript?	In which section is the information available? (Reagents and Tools Table, Materials and Methods, Figures, Data Availability Section)
State if relevant guidelines or checklists (e.g., **ICMJE, MIBBI, ARRIVE, PRISMA**) have been followed or provided.	Not Applicable	
For **tumor marker prognostic studies,** we recommend that you follow the **REMARK** reporting guidelines (see link list at top right). See author guidelines, under 'Reporting Guidelines'. Please confirm you have followed these guidelines.	Not Applicable	
For **phase II and III randomized controlled trials**, please refer to the **CONSORT** flow diagram (see link list at top right) and submit the CONSORT checklist (see link list at top right) with your submission. See author guidelines, under 'Reporting Guidelines'. Please confirm you have submitted this list.	Not Applicable	

Data Availability

Data availability	Information included in the manuscript?	In which section is the information available? (Reagents and Tools Table, Materials and Methods, Figures, Data Availability Section)
Have **primary datasets** been deposited according to the journal's guidelines (see 'Data Deposition' section) and the respective accession numbers provided in the Data Availability Section?	Yes	Data Availability
Were **human clinical and genomic datasets** deposited in a public access-controlled repository in accordance to ethical obligations to the patients and to the applicable consent agreement?	Not Applicable	
Are **computational models** that are central and integral to a study available without restrictions in a machine-readable form? Were the relevant accession numbers or links provided?	Yes	Materials and Methods
If publicly available data were reused, provide the respective **data citations in the reference list**.	Not Applicable	
